# Clinical Manifestations and Associated Disease States with *Mycoplasma genitalium* Infection: Narrative Review and State of the Literature, 2015–2025

**DOI:** 10.1093/ofid/ofaf799

**Published:** 2026-03-30

**Authors:** Caroline E Mullis, Eric A Meyerowitz

**Affiliations:** Division of Infectious Diseases, Department of Medicine, Albert Einstein College of Medicine, Bronx, New York, USA; Division of Infectious Diseases, Department of Internal Medicine, Montefiore Medical Center, Bronx, New York, USA; Division of Infectious Diseases, Department of Medicine, Albert Einstein College of Medicine, Bronx, New York, USA; Division of Infectious Diseases, Department of Internal Medicine, Montefiore Medical Center, Bronx, New York, USA

**Keywords:** cervicitis, *Mycoplasma genitalium*, non-gonococcal urethritis, sexually transmitted infection

## Abstract

*Mycoplasma genitalium* (MG) can be asymptomatic or cause a symptomatic infection. Prevalence is associated with demographic factors as well as risk factors for other sexually transmitted infections, such as increasing number of sex partners. MG is common at genitourinary and rectal sites, and unusual in the pharynx. Coinfections are common. Cervicitis, urethritis, and pelvic inflammatory disease are the most common associated disease states, though many other less common clinical manifestations have been described in individuals with MG infection. Here we review the extensive literature from 2015 through 2025 on these topics and provide an overview for interested clinicians and researchers.

*Mycoplasma genitalium* (MG) is a sexually transmitted infection (STI) that presents with or without symptoms and is associated with a variety of clinical syndromes and disease states. There has been increased clinical interest in MG in the last 10 years, particularly since MG nucleic acid amplification testing has become accessible after United States (US) Food and Drug Administration approval in 2019. Here we review the latest research shedding new light on the prevalence, clinical manifestations, and associated disease states of MG infection.

## METHODS

To identify any relevant studies providing data regarding prevalence and clinical manifestations of MG published between 1 January 2015 and 3 September 2025, we used the following search string in PubMed on 3 September 2025: (“mycoplasma genitalium”) AND ((“2015/01/01”[Date-Publication]: “3000”[Date-Publication])). Relevant studies were cataloged in Microscoft Excel software and a single reviewer (either C. E. M. or E. A. M.) manually reviewed titles, abstracts, and full text (as needed). Articles were included if they provided primary data on prevalence or risk factors, clinical manifestations, and associated disease states. Studies were excluded if they were outside the scope of this review, text was not available, or the manuscript was not in English. We then manually extracted data from the included papers.

## RESULTS

Our search performed on 3 September 2025 identified 1194 manuscripts indexed to PubMed between 1 January 2015 and 3 September 2025. We reviewed titles and abstracts and excluded 767 that were deemed outside the scope of the review. The chief reasons for exclusion included because studies were primarily about diagnosis, treatment, resistance, or the molecular biology of MG infection, or because the manuscripts were reviews or viewpoints that did not provide primary data on the clinical epidemiology of MG infection.

## UPDATES IN TRANSMISSION DYNAMICS

While sexual transmission of MG is well documented [1], we identified some studies in our search window that provide new details into transmission dynamics. A cross-sectional analysis of patients attending an STI clinic in Australia found a lower rate of genitourinary (GU) MG positivity among heterosexual male compared to female contacts of index MG cases: 31.0% (95% confidence interval [CI], 23.0%–39.8%) vs 48.2% (95% CI, 39.7%–56.8%) (*P* = .004) [2]. Among men who have sex with male partners (MSM) contacts of index cases, the rate of positivity was 26.8% (95% CI, 18.9%–36.0%); however, less than half (42.9%) were tested at GU and rectal sites, so the true overall positivity might be expected to be higher.

Male circumcision may be protective against acquisition of urethral MG. While 1 study of national survey participants from the United Kingdom (UK) found no difference in urine positivity between circumcised and uncircumcised men [3], a study from South Africa designed to assess the impact of male circumcision on a variety of STIs found that it was associated with reduced MG risk among men (adjusted odds ratio [aOR], 0.53 [95% CI, .32–.88]) [4].

A study looking at MG positivity among pregnant women in Israel found that 2.3% (5/214) were positive, and documented mother-to-child transmission in 1 of 5 newborns [5]. In this study, maternal samples were vaginal swabs and newborn samples were pharyngeal swabs. Another study in Angola used endocervical swabs on pregnant women and conjunctival swabs on newborns and found that 2 of 19 newborns born to women with MG were positive [6]. These studies underscore the need for further research into the factors associated with transmission after exposure to support the development of preventive strategies.

Several studies have examined persistence of MG infections. In a US-based cohort study, the median duration of MG infection in the absence of curative therapy (ie, for individuals with macrolide resistance treated with azithromycin) was 143 days (range, 21–228 days) [7]. Another US-based cohort including transgender and MSM youth found high rates of persistent positivity of MG at 12 months after initial positive test, including 62 of 140 (44.3%) and 40 of 57 (70%) still positive at rectal and urine sites, respectively [8]. Analysis of samples from a Belgian preexposure prophylaxis study found that the median duration of untreated MG infection was 84 days (interquartile range [IQR], 1–92 days); however, some infections persisted for more than a year [9]. Future research is needed to understand the natural history of MG infection and elucidate factors associated with persistence of infection. Furthering the understanding of factors associated with clearance of MG infection could support the development and implementation of novel treatment strategies.

## PREVALENCE AND RISK FACTORS FOR INFECTION

We identified 241 studies that provided information about MG prevalence in numerous countries and a variety of settings (Supplementary Table 1). Some studies included both symptomatic and asymptomatic individuals, and some only symptomatic or asymptomatic individuals.

Few studies shed light on overall population prevalence of MG. One study using aggregated cervical specimens from women undergoing cervical cancer screening in the US found an overall prevalence of 1.95% (Table 1) [26]. US national survey participants aged 14–59 years had an overall prevalence in the urine of 1.7% [25]. Among national survey participants aged 16–74 years in the UK, positivity in the urine was 1.2% and 1.3% for men and women, respectively [13].

**Table 1.** Selected Studies With Prevalence and Risk Factors for *Mycoplasma genitalium* Infection, 2015–2025

First Author, Year	Country	Setting	Population	Prevalence, % (95% CI), MG Positive (no.)/Total (No.)
Studies including both symptomatic and asymptomatic individuals				
Napierala Mavedzenge, 2015 [10]	Zimbabwe	Multiple centers for reproductive and general healthcare	Nonpregnant women with recent HIV seroconversion	Endocervical: 10.7%, 14/131
Chen, 2015 [11]	China	Population survey with outreach in 13 cities	Males with HIV	Urine: 20.1%, 310/1541
				Among PWH with CD4 <350 vs >350 cells/μL: 24.0% vs 15.3%, *P* < .001
Deguchi, 2015 [12]	Japan	STI clinic	Female sex workers	Vaginal: 14.1%, 21/149
				Throat washing: 0.7%, 1/149
Homfray, 2015 [3]	UK	National survey participants	Men aged 16–74 y	Urine: 1.9% (.7%–.51%), 5/284 (circumcised)
				Urine: 1.0% (.6%–1.7%), 16/1566 (uncircumcised)
				Not significant
Sonnenberg, 2015 [13]	UK	National survey participants	Men and women aged 16–74 y	Urine: 1.2%*^a^* (.7%–1.8%), 24/1875 (men)
				Urine: 1.3%^a^ (.9%–1.9%), 48/2632 (women)
				Black men: aOR, 12.05 (3.68–39.44)
Munson, 2016 [14]	United States	Aggregation of test results from a central lab	Females undergoing chlamydia/gonorrhea testing	GU: 10.3%, 106/1032 (outpatient OB)
				GU: 15.2%, 7/46 (inpatient OB)
				GU: 6.9%, 18/261 (suburban family care)
				GU: 10.7%, 41/384 (urban family care)
				GU: 14.6%, 110/755 (ED)
Munson, 2017 [15]	United States	STI clinic	Men receiving care at study site	Urine: 6.6%, 97/1465
				Pharyngeal: 0.9%, 13/1493
				Rectal: 5.8%, 48/823
Hakre, 2017 [16]	United States	HIV clinic	US Air Force personnel living with HIV undergoing annual medical exam	All sites: 18.6%, 19/102 (overall)
				Urine: 9.3% (4.3%–16.9%), 9/97
				Rectal: 11.1% (5.7%–19.0%), 11/99
				Pharyngeal: 0.98% (.0%–5.3%), 1/102
Unemo, 2018 [17]	Denmark, Norway, Sweden	Multiple STI clinics	Consecutive males and females	Vaginal: 8.2%, 138/1683
				Cervical: 8.2%, 23/280
				Urethral: 9.0%, 26/290 (females)
				Rectal: 8.1%, 3/37 (females)
				Pharyngeal: 0%, 0/74 (females)
				Urine: 6.0%, 165/2740 (males)
				Urethral: 7.4%, 56/754 (males)
				Rectal: 6.3%, 15/237 (males)
				Pharyngeal: 1.5%, 4/260 (males)
Dionne-Odom, 2018 [18]	United States	HIV primary care clinic	MSM >18 y with receptive anal intercourse in last 30 d	All sites: 17.2%, 27/157
				GU: 10.8%, 17/157
				Rectal: 6.4%, 10/157
				No sig difference in organism load by anatomic site
Upton, 2018 [19]	New Zealand	Outpatient clinics of Aukland and Northland regions (population ∼1.6 million)	All samples tested for CT/NG in individuals ≥13 y	All sites: 5.1%, 134/2643
				No sig difference between men and women (aOR, 1.35, *P* = .19); sig more prevalent in those aged <25 y vs older (aOR, 3.04, *P* < .001) and Māori/Pacific vs European (aOR, 1.82, *P* = .002) or Asian (aOR, 3.02, *P* = .002)
Lillis, 2019 [20]	United States	STI clinic	Nonpregnant women	GU: 17.5%, 70/400
				MG independently associated with age <25 y (*P* < .03) and with reporting ≥2 partners in last 12 mo
Ferré, 2019 [21]	Togo	National, population-based survey	MSM ≥18 y, transgender and gender diverse excluded	Rectal: 15.0% (10.1%–19.9%), 31/207
Manhart, 2020 [22]	United States	Multiple sites including STI clinics (AMES)	Females and males aged ≥14 y	GU: 10.1%, 176/1737 (females)
				GU: 10.6%, 165/1563 (males)
				MG risk sig higher in women aged 15–24 y vs aged 35–49 y (aOR, 5.05 [3.01–8.46]); men aged 15–24 y vs 35–49 y (aOR, 1.91 [1.20–3.02]); Black vs White race (aOR, 1.88 [1.30–2.72] for women and 2.02 [1.38–2.96] for men); non-Hispanic vs Hispanic ethnicity (aOR, 1.97 [1.25–3.10] for women and 1.80 [1.14–2.85] for men); and STI clinics, family planning clinics, public health clinics, and EDs vs clinical research centers (aOR, 2.71 [1.03–6.34])
Rowlinson, 2021 [23]	United States	STI clinic	Cisgender male STI clinic patients ≥16 y reporting sex exclusively with female partners, PWH excluded	Urine: 11.1%, 34/307
				Urine: 5.0%, 10/200 (non-NGU)
				Urine: 22.4%, 24/107 (NGU)
				Sig higher prevalence among men with NGU, *P* < .01
Han, 2021 [24]	China	Multiple STI clinics	Patients ≥18 y	All sites: 7.2%, 97/1374
				Rectal: 2.2%, 30/1377
				GU: 5.6%, 77/1374
				Sig increased risk for rectal MG among MSM (aOR, 40.4 [3.9–421.2]), those with genital symptoms (aOR, 3.5 [1.2–10.5]), and having infection with urogenital MG (aOR, 7.2 [2.9–18.0]); decreased risk for males vs females (aOR, 0.2 [.1–.7])
Torrone, 2021 [25]	United States	National survey participants (NHANES)	People aged 14–59 y	Urine: 1.7%, 67/3945
				MG prevalence sig higher among non-Hispanic Blacks vs non-Hispanic Whites (PR, 2.5 [1.0–6.4]), higher among persons reporting ≥10 lifetime partners vs 0–4 (PR, 3.0 [1.2–7.4])
Hammer, 2021 [26]	United States	Aggregated specimens from cervical cancer screening covering 70% of state of New Mexico	Women aged 21–64 y receiving cervical cancer screening	Vaginal: 1.95%^a^, 1651/84 686
Begnis, 2021 [27]	France	STI clinic	All patients screening for CT/MG/NG	All sites: 4.88%, 101/2969
				GU: 4.38% (3.51%–5.40%), 87/1987
				Rectal: 3.06% (1.47%–5.62%), 10/327
				Pharyngeal: 0.61% (.25%–1.27%), 69/1136
Latimer, 2022 [28]	Australia	STI clinic	Women ≥18 y	GU: 6% (5%–8%), 83/1303
				No sig difference between asymptomatic (5% [2%–9%] and symptomatic women (7% [5%–8%]), *P* = .506
Shipitsyna, 2023 [29]	Guatemala, Malta, Morocco, Peru, South Africa	Multiple STI clinics, part of WHO initiative global project on point-of-care tests for STIs	Consecutive patients receiving STI care at study sites	Urine: 4.3%, 60/1384 (MSM)
				Rectal: 12.4%, 173/1397 (MSM)
				Vaginal: 19.1%, 259/1359 (women)
				MG sig more prevalent in rectal than urine samples among MSM, *P* < .0001
Bjartling, 2024 [30]	Sweden	STI clinic	Consecutive MSM receiving care who received CT/NG testing	Overall: 10.5%, 64/609
				Urine: 3.9%, 24/609
				Rectal: 7.6%, 46/609
				Sig more likely to be positive from rectal swab, *P* = .007
Proudmore, 2024 [31]	Australia	Multiple sites, including STI clinics	Individuals undergoing STI screening	All sites: 12.6%, 1540/12178
				Correctional facilities: 21.3%, 660/3103
				Antenatal: 7.2%, 127/1767
				ID clinics: 7.2%, 193/2695

Abbreviations: AMES, Aptima *Mycoplasma genitalium* Evaluation Study; aOR, adjusted odds ratio; CI, confidence interval; CT, *Chlamydia trachomatis*; ED, emergency department; GU, genitourinary; HIV, human immunodeficiency virus; ID, infectious diseases; MG, *Mycoplasma genitalium*; MSM, men who have sex with male partners; NG, *Neisseria gonorrhoeae*; NGU, nongonococcal urethritis; NHANES, National Health and Nutrition Examination Survey; OB, obstetrics; PWH, people with human immunodeficiency virus; PR, prevalence ratio; sig, significant; STI, sexually transmitted infection; UK, United Kingdom; WHO, World Health Organization.

^a^weighted prevalence

On the other hand, targeted testing of certain marginalized groups, such as MSM, will find much higher rates of MG infection. A study including MSM (that excluded transgender and gender-diverse individuals) in Togo found a rectal positivity rate of 15% [21]. A study of nonpregnant women with recent human immunodeficiency virus type 1 (HIV) seroconversion in Zimbabwe found that endocervical MG positivity was 10.7% [10].

Several studies have shown that MG prevalence is associated with certain demographic factors (Table 1). For instance, the Aptima *Mycoplasma genitalium* Evaluation Study (AMES) conducted at 21 US STI clinics found an overall MG prevalence of 10.3%, with an age-determined risk for both males and females [22]. Among females, compared to the reference age group (35–49 years) with a prevalence of 4.7% (95% CI, 3.0%–7.2%), the prevalence was 19.8% (95% CI, 16.4%–23.8%; odds ratio [OR], 5.05 [95% CI, 3.01–8.46]) for age 15–24 years; 9.1% (95% CI, 7.2%–11.3%; OR, 2.03 [95% CI, 1.20–3.43]) for age 25–34 years; and 0.7% (95% CI, .1%–4.1%; OR, 0.15 [95% CI, .00–.98]) for age ≥50 years. Among males in the same study, prevalence was 9.4% (95% CI, 6.9%–12.7%) for the reference age group 35–49 years vs 16.5% (95% CI, 12.6%–21.2%; OR, 1.91 [95% CI, 1.20–3.02]), 12.8% (10.3%–15.7%; OR, 1.41 [95% CI, .93–2.14]), and 2.3% (95% CI, 1.1%–4.7%; OR, 0.23 [95% CI, .08–.53]) for ages 15–24, 25–34, and ≥50 years, respectively. A study of 400 women aged ≥18 years attending an STI clinic in New Orleans found that MG was more likely in those <25 years versus the reference of >30 years, further evidence that younger, reproductive-aged individuals may be more likely to have an MG infection [20].

In the US (AMES), Black individuals had a significantly increased risk of testing positive compared to White individuals (OR, 1.88 [95% CI, 1.30–2.72] and 2.02 [95% CI, 1.38–2.96] for females and males, respectively) [22]. Similarly, a study of national survey participants in the US among people aged 14–59 years found a significantly higher MG prevalence among non-Hispanic Black vs non-Hispanic White individuals (prevalence ratio, 2.5 [95% CI, 1.0–6.4]). Among national survey participants in the UK, Black race was associated with increased risk for MG (aOR, 12.05 [95% CI, 3.68–39.44]). In a population-based study including many outpatient clinics in New Zealand, Māori/Pacific and Asian people were more likely to test positive for MG than Whites (aOR, 1.82, *P* = .002 and aOR, 3.02, *P* = .002, respectively) [19].

MG has also been associated with increased number of sexual partners. A study at an STI clinic that included nonpregnant women found that risk for MG was elevated for those with ≥2 partners in the last 12 months [20]. Among national survey participants in the US, risk was increased for those reporting ≥10 lifetime partners [25]. A study in China found that MG was more likely among people with HIV (PWH) with CD4 count <350 cells/μL versus PWH with CD4 count >350 cells/μL [11].

The prevalence of MG also depends on the nature of the location where testing occurs. A multicenter study in the US found that positivity rates were higher in STI clinics, family planning centers, public health clinics, and emergency departments than in clinical research centers (aOR, 2.71 [95% CI, 1.03–6.34]) [22]. A multicenter study from the US found that the positivity rate of urogenital specimens was 6.9% in a suburban family care office and 14.6% in an emergency department setting [14]. A multicenter study in Australia found an overall prevalence of MG of 12.6%, but a site-specific prevalence of 21.3% at correctional settings and 7.2% at antenatal clinics and infectious diseases clinics [31].

Highlighted studies reveal how certain populations may be more likely to have MG infection. MG prevalence was noted to be higher in individuals with younger age, Black race, or more sexual partners and those accessing care in certain clinical care settings such as correctional facilities and emergency departments, underscoring the importance of considering MG infection especially in these populations.

## ANATOMIC SITES OF INFECTION

The most frequently tested anatomic sites for MG are GU (urine, penile urethra, vagina, and cervix), rectal, and pharyngeal. A study among MSM at an HIV clinic in the US found no difference in organism load between genitourinary and rectal sites [18]. Infection rates may vary greatly by anatomic site tested, with MG only rarely recovered from the pharynx. Among patients at an STI clinic in France, positivity was 4.4%, 3.1%, and 0.6% for GU, rectal, and pharyngeal MG, respectively [27]. Among men receiving care at an STI clinic in the US, positivity was 6.6%, 5.8%, and 0.9% from urine, rectal, and pharyngeal sites, respectively [15]. A study of female sex workers from Japan found 14.1% positivity of MG on vaginal swabs, but only a 0.7% positivity from oral throat washings [12]. A study of PWH in the US Air Force found 9.3% positivity from urine, 11.1% positivity from rectal swabs, and 0.1% positivity from pharyngeal swabs [16]. A multicenter, multicountry study from Europe found positivity of 8.2%, 8.1%, and 0% for vaginal, rectal, and pharyngeal swabs from women, respectively, and 6.0%, 6.3%, and 1.5% positive from urine, rectal, and pharyngeal swabs for men, respectively [17]. Among MSM at an HIV primary care clinic in the US, positivity was 10.8% and 6.4% from GU and rectal sites, respectively [18].

A study in China including multiple STI clinics found that rectal MG was most likely among MSM (aOR, 40.4 [95%CI 3.9–421.2]) [24]. In a multicenter study including STI clinics in 5 countries, rectal MG positivity was higher than urine for among MSM [29]. Similarly, among consecutive MSM receiving care at an STI clinic in Sweden tested for *Chlamydia trachomatis* (CT) and/or *Neisseria gonorrhoeae* (NG), MG was significantly more likely to be positive from the rectal swab than urine (7.6% vs 3.9%, *P* = .007) [30]. Clinicians should consider the presence of rectal MG infection especially in MSM.

Rates of MG positivity may vary by the presence or absence of symptoms. Among cisgender males reporting exclusively cisgender female partners at a US STI clinic, MG positivity in the urine was significantly higher for individuals with nongonococcal urethritis (NGU) than for those without (22.4% vs 5.0%, *P* < .01) [23]. Another study among women at an STI clinic in Australia found no significant difference among those with and without genital symptoms for GU MG infection (5% [95% CI, 2%–9%] vs 7% [95% CI, 5%–8%], for asymptomatic and symptomatic women, respectively; *P* = .506) [28].

While rates of GU and rectal MG infections were often similar in studies, pharyngeal MG infections were rarely reported. Rectal MG may be more common than urethral MG among MSM, and rates of MG infection are higher among those with than without symptoms.

## CLINICAL SYNDROMES AND PRESENTATIONS

The most common clinical syndromes associated with MG infection were urethritis, vaginitis, cervicitis, and pelvic inflammatory disease (PID) (Supplementary Table 2) [32]. Evaluation of patients who were tested for CT and NG at a sexual health clinic in the US detected MG in 26.8% of males with urethritis, 21.1% of females with vaginitis, 11.8% with cervicitis, and 15.4% with PID [32]. However, our review also found literature describing MG infections causing prostatitis, proctitis, and other clinical syndromes (Table 2). Additionally, the literature evaluated potential complications of MG infection such as infertility and preterm birth.

**Table 2.** Select Studies on Clinical Syndromes and Complications Associated With *Mycoplasma genitalium*, 2015–2025

First Author, Year	Country	Population	Findings
Clinical syndromes			
Alamon-Reig, 2022 [33]	Spain	Patients diagnosed with MG	73/196 had symptoms (37.3%), 119/196 (60.7%) symptoms absent, 4/196 (2%) missing data
Johnson, 2025 [34]	United Kingdom	Patients with MG	Urethritis was the most common clinical indication for testing (63.8% [217/340]), followed by cervicitis (3.8% [13/340]), PID (4.1% [14/340]), and proctitis (0.3% [1/340]). Other indications (16.8% [57/340]). Contacts of MG infections made up 11.2% (38/340).
le Roux, 2017 [35]	South Africa	A group of men with symptoms and/or signs of urethritis and a group of men without urethritis signs and symptoms	MG was detected more frequently in the symptomatic group than the asymptomatic group (13.7% [41/300] vs 5.3% [4/75], *P* = .002). Among the symptomatic patients, MG was detected in 18.0% (17/94) of patients with visible urethral discharge and in 11.7% (24/206) of patients with burning on urination. There were significant differences between median bacterial loads of the discharge, burning on urination, and asymptomatic groups (*P* < .01) and between the symptomatic and asymptomatic groups (*P* = .002).
Lee, 2022 [36]	Hong Kong	Males presenting with NGU	Higher prevalence of MG (vs MG negative) in patients presenting with urethral discharge (12% vs 6%; OR, 2.16 [95% CI, 1.10–4.23]; *P* = .02) and patients with symptom duration >2 wk (14% vs 6%; OR, 2.34 [95% CI, 1.10–4.97]; *P* = .03)
Latimer, 2022 [28]	Australia	Women	In women without BV, MG was strongly associated with cervicitis (aOR, 4.38 [95% CI, 1.69–11.33]; *P* = .002), but this association was not found in women with BV. MG was not associated with any other clinical signs, including vaginal polymorphonuclear lymphocyte count.
Chow, 2021 [37]	Australia	Two groups of MSM: (1) men presenting with symptomatic proctitis and (2) asymptomatic men not reporting symptoms of proctitis	MG was more commonly detected among men with proctitis (9.4% [95% CI, 7.0%–12.3%]; 47/499) vs asymptomatic men (5.1% [95% CI, 3.4%–7.4%]; 26/506; *P* = .010). The most common symptoms reported were anorectal pain (n = 38 [81%]), anal bleeding (n = 18 [38%]), anal discharge (n = 13 [28%]), and tenesmus (n = 11 [23%]).
Complications of MG infection			
Li, 2020 [38]	China	Male partners from infertile couples (n = 30 094)	MG prevalence: 749/30 094, 2.49% (95% CI, 2.31%–2.66%), nearly all asymptomatic. Semen concentration and total sperm count were lower in males with infection than in males without infection. After antibiotic treatment, the mean values of the semen parameters increased from those measured before treatment, especially semen concentration, except for leukocyte concentration.
Hu, 2023 [39]	United States	Pregnant women seen in OB/GYN clinics	MG was detected at a higher frequency in patients with an outcome of preterm birth (11.4% vs 7.8%), small for gestational age (14.8% vs 7.7%), or fetal growth restriction (15.4% vs 8.2%) but was not significantly associated with these outcomes.
Kayem, 2018 [40]	France	Women with a singleton pregnancy who underwent amniocentesis from 16 to 20 wk for Down syndrome screening	1.3% (13/1016) of amniotic fluid was positive for MG. Bacterial colonization of amniotic fluid was rare in second trimester.

Abbreviations: aOR, adjusted odds ratio; BV, bacterial vaginosis; CI, confidence interval; MG, *Mycoplasma genitalium*; MSM, men who have sex with male partners; NGU, nongonococcal urethritis; OB/GYN, obstetrics and gynecology; OR, odds ratio; PID, pelvic inflammatory disease.

### Asymptomatic MG

Patients with MG can be asymptomatic at presentation. The percentage of patients without symptoms at the time of presentation varied from 0% in 8 patients with MG in Morocco [41] to 90.6% of pregnant females with MG in Zambia [42]. In a descriptive study of 196 cases of MG in Spain, 37% (73/196) had symptoms, 61% (119/196) had no symptoms, and 2% (4/196) had missing data [33]. Symptoms may depend on the anatomic site of the MG infection. A study in males seeking care at an STI clinic in Poland found that while 22% of GU MG was symptomatic, all extragenital (rectal and pharyngeal) MG was asymptomatic [43]. The percentage of MG with an asymptomatic presentation may also differ between males and females. One study found that all males with MG were symptomatic while 78.8% of females with MG were symptomatic [44].

In a study of 340 patients diagnosed with MG in the UK, urethritis (63.8%) was the most common clinical indication for testing, followed by other indications (16.8%), contact with MG infection (11.2%), cervicitis (3.8%), PID (4.1%), and proctitis (0.3%) [34]. A study of 502 patients diagnosed with MG in the US found that most were asymptomatic (36.1%), followed by urethritis (29.5%), vaginal symptoms (28.3%), PID (4.6%), and other presentations (4.2%) [45].

Current US Centers for Disease Control and Prevention guidelines do not recommend MG testing in asymptomatic individuals [46]. However, asymptomatic MG is prevalent in cross-sectional studies. Further research is needed to understand the clinical implications, role in ongoing transmission, and potential benefits of diagnosing and treating asymptomatic MG.

### Urethritis

Urethritis is well established as a clinical presentation of MG infection, especially in males. In men with urethritis, MG was detected in 18% (17/94) of those with visible urethral discharge and 12% (24/206) of those with burning on urination [35]. In another study, MG was associated with serous urethral discharge (aOR, 2.99 [95% CI, 1.41–6.34]; *P* = .004), purulent urethral discharge (aOR, 2.88 [95% CI, 1.20–6.91]; *P* = .018), and urethral burning or irritation (aOR, 1.92 [95% CI, 1.08–3.43]; *P* = .027) [47]. In patients with NGU, MG prevalence ranged from 6.5% (11/170) in males in Brazil [48] to 23% (35/155) in males in the US [49], with most additional studies reporting MG in around 20% of patients with NGU [23, 50–52].

MG should also be considered in patients with chronic urethritis. In a study of males in Sweden, MG was associated with both acute NGU (aOR, 33 [95% CI, 7.6–144]; *P* < .001) and chronic NGU, defined as >30 days of symptoms (OR, 91 [95% CI, 14–589]; *P* < .001) [53]. Higher prevalence of MG (vs MG negative) was also found in patients with urethritis symptoms for >2 weeks (14% vs 6%; OR, 2.34 [95% CI, 1.10–4.97]; *P* = .03) [36]. In individuals with urethritis, the burden of bacteria may be related to the presence or absence of symptoms. Two studies found that the bacterial load was higher in those symptomatic with urethritis compared to those without symptoms [35, 53].

While most studies evaluating the clinical syndrome of urethritis focused on males, 1 study in females diagnosed with chronic papillary urethritis found that 25% (37/147) of patients were positive for MG [54]. In 90 women with urethral polyps, 9% tested positive for MG [55]. These 2 studies underscore the need to consider MG as an etiology in females with urethritis.

### Cervicitis

Cervicitis is a frequently described clinical syndrome associated with MG in females. In a meta-analysis, MG infection was associated with increased risk of cervicitis (pooled OR, 1.66 [95% CI, 1.35–2.04]) [56]. Up to 25% of patients with cervicitis could have a MG infection [57]. However, the presentation of cervicitis in females can be confounded by concomitant infections along with MG. A study of women from Australia illustrates this: In women without bacterial vaginosis (BV), MG was strongly associated with cervicitis (aOR, 4.38 [95% CI, 1.69–11.33]; *P* = .002), but this association was not found in women with BV [28]. A study from China evaluated the effect of MG and CT infections on leukorrhea and found that in 37% (34/92) of MG only (CT negative), 23% (114/500) of those negative for MG and CT, and 74% (20/27) of those with MG and CT had >15 white blood cells per high-power field, suggesting that while MG alone increased leukorrhea, combined MG and CT infections can even further increase likelihood of leukorrhea [58]. MG infection should be considered along with other bacterial STIs in females with a clinical syndrome of cervicitis.

### Pelvic Inflammatory Disease

PID is inflammation and infection of the upper genital tract in women, including endometritis, salpingitis, tubo-ovarian abscess, and pelvic peritonitis. PID is frequently associated with CT and NG infections; however, other organisms are increasingly being implicated in PID. A 2024 systematic review and meta-analysis evaluating the association between MG and PID found that MG was significantly associated with PID (pooled OR, 1.67 [95% CI, 1.24–2.24]) and estimated a pooled positivity of MG in PID as 10.3% (95% CI, 5.63%–15.99%), with highest positivity of MG in PID in the Americas [59]. PID often manifests as lower abdominal pain but can present with a variety of signs and symptoms, making diagnosis challenging. In an Australian study, women with MG-related PID were more likely to have lower abdominal tenderness (aOR, 2.29 [95% CI,, 1.14–4.60]), but less likely to have a modest elevation in vaginal polymorphonuclear neutrophil counts, compared to women with CT-related PID [60]. In a study that included 56 females who received a bimanual pelvic exam, a higher proportion of those with cervical motion/adnexal tenderness had MG (2/5 [40%]) versus those without cervical motion/adnexal tenderness (4/51 [7.8%]) (*P* = .027) [61]. However, both participants in this study with cervical motion/adnexal tenderness and MG were coinfected with another STI pathogen. Endometritis associated with MG has also been described. In women with mucopurulent cervicitis and likely CT infection, 48.7% of MG-positive women had endometrial infection that was diagnosed on biopsy [62]. In females with PID, diagnostic testing for MG infection should be considered.

### Proctitis

Anorectal MG infections were described in both males with symptoms (ie, proctitis) or in males without any symptoms. In one study in the US, only 6.3% of patients with a rectal swab positive for MG had any rectal symptoms [63]. MG was more commonly identified in MSM with symptomatic proctitis (9.4% [95% CI, 7.0%–12.3%]; 47/499) compared to asymptomatic MSM (5.1% [95% CI, 3.4%–7.4%]; 26/506) (*P* = .01) [37]. In the same study, the most common symptoms of proctitis were anorectal pain (n = 38 [81%]), anal bleeding (n = 18 [38%]), anal discharge (n = 13 [28%]), and tenesmus (n = 11 [23%]). In MSM with anoproctitis in France, MG was less likely if the patient presented with an anal fissure (11% [36/319] vs 2% [1/46]; *P* = .01) [64]. In a study of gay and bisexual men and transgender women with documented proctitis on proctoscopy, 6.8% (10/147) had MG as the only identified organism, again demonstrating the importance of considering MG in patients with proctitis [65]. Testing for MG should be considered in persons presenting with symptoms of proctitis such as anorectal pain, anal bleeding, and anal fissure, particularly if testing for common etiologies (eg, CT, NG, herpes simplex viruses, mpox, and syphilis) is negative.

### Other Clinical Syndromes

In addition to the most common clinical syndromes of urethritis, cervicitis, and PID described above, our review of the literature found less common presentations. One study found an association between MG and prostatitis with an MG prevalence of 10% in those with prostatitis compared to 3% in the control group (*P* = .005) [66]. Three case reports were identified in our literature review of unique MG presentations: 1 patient with Fitz-Hugh–Curtis syndrome associated with MG [67], 1 with prosthetic valve endocarditis with MG identified on 16s sequencing [68], and 1 with reactive arthritis after an anogenital MG infection [69]. While uncommon, consideration should be made for MG infection in atypical presentations when a pathogen has not otherwise been identified.

## COMPLICATIONS FROM MG INFECTION

### Infertility

Two studies from India highlight potential associations of MG with female infertility. The first found tubal occlusion in 5 (33%) women with MG and in none of the women without MG (*P* < .001) [70]. The second study found that MG was more common in women with unexplained infertility (13.3%) versus those with an identified, underlying cause (4.5%) (*P* < .05) [71]. However, a study in Cameroon found no significant different in the percentage with MG in those with secondary infertility and pregnant women (9/151 [6%] women with secondary infertility vs 17/265 [7%] pregnant women; *P* = .68) [72]. In a 2017 systematic review and meta-analysis, while the risk of infertility was elevated in women with MG infection, it was not statistically significant (pooled OR, 2.43 [95% CI, .93–6.34]) [56].

Studies also had mixed results in males. While most studies found no MG infections in males with infertility [73–75], one noted an association between MG and male infertility among males in Iran (9.7% of infertile men with MG vs 1.2% of control group; *P* = .001) [76]. In 1 study, semen concentration and total sperm count were lower in males with MG infection than in males without infection [38]. After antibiotic treatment, the mean values of the semen parameters increased from those measured before treatment, especially semen concentration, but not leukocyte concentration. While some studies are suggestive of an association between MG and both female and male infertility, data are inconclusive. Further studies are needed to evaluate the impact of MG on infertility.

### Pregnancy Complications

Preterm birth (pooled OR, 1.89 [95% CI, 1.25–2.85]) and spontaneous abortion (pooled OR, 1.82 [95% CI, 1.10–3.03]) were associated with MG infection in a meta-analysis [56]. Studies from our review also noted the association between preterm birth and MG infection [39, 77]. One study of women in the US found that MG was detected at a higher frequency in patients with an outcome of preterm birth (11.4% vs 7.8%), small for gestational age (14.8% vs 7.7%), or fetal growth restriction (15.4% vs 8.2%) but was not significantly associated with these outcomes [39]. MG infection was associated with lower birth weight in 2 separate studies [78, 79]. MG infection was associated with a lower mean birthweight of 166.9 g lower (95% CI, 324.2 g lower to 9.7 g lower; *P* = .04) compared to those without MG infection [79]. MG was also associated with ectopic pregnancy in 2 studies [80, 81].

In women undergoing amniocentesis, 1 study found 0% (0/344) with MG [82] and another found 1.3% (13/1016) with MG [40], suggesting that bacterial colonization of amniotic fluid with MG is not common. Additional research is needed to understand the effects of MG infection on pregnant people.

## COINFECTIONS ASSOCIATED WITH MG

Overall, we identified 90 studies that described coinfections in patients with MG (Supplementary Table 3). Most commonly, studies reported on other bacterial STIs such as CT, NG, and *Trichomonas vaginalis* (TV) (Table 3). However, studies describing coinfection with HIV, human papillomavirus (HPV), BV, and other infections were noted.

**Table 3.** Select Studies on Infections Reported With *Mycoplasma genitalium*, 2015–2025

First Author, Year	Country	Population	Findings
Manhart, 2023 [32]	United States	Persons tested for CT and NG at sexual health clinic with attempted equal enrollment in each of the 4 groups (symptomatic males, asymptomatic males, symptomatic females, asymptomatic females)	MG was detected in 28.2% of CT and 23.9% of NG infections. After adjusting for sampling criteria (site, birth sex, symptoms), MG was associated with CT (aPR, 1.7 [95% CI, 1.13–2.53]) but not NG.
Mullis, 2024 [45]	United States	Patients testing positive for MG	12.4% (57/460) with CT, 6.7% (31/460) with NG, 8.5% (32/376) with TV, 1.5% (6/397) with syphilis, 53.6% (45/84) with BV. Females with BV had a higher percentage of microbiologic cure of MG relative to both those testing negative for BV (77% vs 40%, *P* < .01) and all other females (those testing negative and those not tested for BV) (77% vs 6 43%, *P* < .01).
Wiesenfeld, 2021 [83]	United States	Women diagnosed with acute PID	At baseline, 41 (18%) of women had MG and BV was present in 127 women (55%). Even though metronidazole has no activity against MG, cervical infection following PID treatment was less frequent in women receiving metronidazole than in women in the placebo-containing group (4% vs 14%, *P* < .05). BV (20% vs 54%, *P* < .001) and TV (5% vs 12%, *P* = .10) were also less prevalent in women who were randomized to metronidazole.
Napierala Mavedzenge, 2015 [10]	Zimbabwe	Women with new HIV-1 diagnosis <6 mo	BV associated with increased prevalence of MG (aOR, 2.24 [95% CI, 1.03–4.85]) in multivariable model. Genital HIV-1 RNA was more likely in endocervical samples with MG compared to no MG (71.4% vs 58.3%; aOR, 2.81 [95% CI, 1.01–7.83])
Xie, 2021 [84]	China	Women undergoing gynecological exam	1.5% (10/668) prevalence (all in HPV-positive cases), The proportion of patients with MG infection was 4.13% (5/121) and 0.91% (5/547) in the CIN group and the control group, respectively, suggesting that the risk of CIN was higher in patients with MG infection (OR, 4.207 [95% CI, 1.160–15.260]; *P* = .029).
Rizzo, 2024 [85]	Italy	Sexually active and reported unprotected intercourse	Rectal HPV: Among those positive for MG and HPV, 34% (18/53) of hr-HPV, 35% (13/37) of probable/possible hr-HPV, and 31% (13/42) of low-risk HPV were positive for MG.

Abbreviations: aOR, adjusted odds ratio; aPR, adjusted prevalence ratio; BV, bacterial vaginosis; CI, confidence interval; CIN, cervical interepithelial neoplasia; CT, *Chlamydia trachomatis*; HIV-1, human immunodeficiency virus type 1; HPV, human papillomavirus; hr-HPV, high-risk human papillomavirus; MG, *Mycoplasma genitalium*; NG, *Neisseria gonorrhoeae*; OR, odds ratio; PID, pelvic inflammatory disease; TV, *Trichomonas vaginalis*.

### *Chlamydia trachomatis*, *Neisseria gonorrhoeae*, and *Trichomonas vaginalis*

Among those with MG infection, coinfection with other STIs including CT, NG, and TV was frequently reported. In a 2018 study, women aged 13–29 years in the US with MG were 3.4 times more likely to have coinfections compared with those with other STIs (OR, 3.4 [95% CI, 1.17–10.3]; *P* = .02) [86]. Among participants with MG, rates of CT coinfection were reported up to 40% in a study of MSM in Vietnam [87], and rates of NG coinfection were reported up to 36% (4/11) in a small study of symptomatic men in the US [88]. Importantly, in 1 study enrolling MSM, MG was detected in 13% (95% CI, 9%–18%; 27/212) of rectal CT-positive samples and 14% (95% CI, 9%–19%; 29/212) of rectal NG-positive samples [89] underscoring the potential need to co-test at nonurogenital sites. A study in France found that CT coinfection was more likely in women with MG than men (20/78 [26%] vs 41/319 [13%]; *P* = .006) and that NG coinfection was more likely in men (27/319 [8.5%]) versus women (0/78 [0%]) with MG (*P* = .008) [90].

In patients with MG at a single New York City health system, 12.4% (57/460) had a CT coinfection, 6.7% (31/460) had an NG coinfection, and 8.5% (32/376) had a TV coinfection [45]. In patients tested for CT or NG at STI clinics in the US, MG was detected in 28.2% of CT and 23.9% of NG infections [32]. MG was associated with CT (adjusted prevalence ratio, 1.7 [95% CI, 1.13–2.53]) but not NG in this study. Other studies similarly noted that CT infection is associated with a higher odds or prevalence of MG infection [91–93].

Only 1 study reported on MSM with lymphogranuloma venereum (LGV) proctitis and found that 1 of 26 patients with LGV had an MG coinfection [94].

While coinfections with CT, NG, and TV were prevalent, testing for MG in clinical settings is likely underperformed on patients being tested for other STIs. In a single health system in the US, only 1.7% (5572/332 619) of patient encounters with testing for CT, NG, or TV also had concurrent MG testing performed [95].

MG is frequently observed in the presence of CT, NG, and TV infections. Clinical consideration and testing for MG should be made in individuals with CT, NG, and TV infections, especially in cases where symptoms persist after directed treatment.

### Bacterial Vaginosis

Several studies noted associations between BV and MG infection. Four studies found that MG infections were more common in women with BV infection or BV-associated microbiota [10, 29, 96, 97]. Odds of MG in those with BV were found to be 2.24 (95% CI, 1.03–4.85) [10] in women with newly diagnosed HIV in Zimbabwe, 1.97 (95% CI, 1.14–3.39) in women with vaginitis symptoms in the US [96], 2.88 (95% CI, 1.19–7.16) in women seeking routine gynecologic care in Russia [29], and 1.98 (95% CI, 1.29–2.87) in women in the US [97].

An unanticipated finding in a randomized controlled trial evaluating treatment for PID (ceftriaxone intramuscularly and doxycycline), with or without metronidazole, found that cervical MG was reduced at 30 days in those treated with metronidazole relative to those with placebo (4% vs 14%, *P* < .05) as was BV (20% vs 54%, *P* < .001) [83]. While metronidazole is a common treatment for BV, it has no known direct activity against MG, suggesting that the drug's activity against anaerobes including organisms associated with BV may facilitate an individual's ability to clear an MG infection. A retrospective observational study of patients with MG in the US found an association between BV diagnosis and subsequent microbiologic cure (40% without BV, 77% with BV; *P* < .01) [45]. Although numbers were small in the subset with simultaneous BV and MG testing, receipt of metronidazole was not associated with microbiologic cure in this study. Additional studies are needed to understand how changes in the vaginal microbiome associated with BV affect clearance of MG infection.

### Human Immunodeficiency Virus

In many studies, MG was found to be associated with HIV, noting either a higher prevalence of MG among PWH [98–103] or that persons with MG infection were more likely to be living with HIV [104]. A systematic review and meta-analysis of studies evaluating MG and the association with HIV through June 2008 identified 19 studies, finding that participants with MG were more likely to be PWH (OR, 2.01 [95% CI, 1.44–2.79]) [105].

A few studies evaluated potential effects of coinfection with HIV and MG infection. A study in women newly diagnosed with HIV in Zimbabwe found that genital HIV- RNA was more likely to be detected in endocervical samples with MG (aOR, 2.81 [95% CI, 1.10–7.83]), suggesting a potential relationship between MG and HIV genital shedding [10]. In a study of young adult males in the US, MG was found to be a significant risk factor for HIV seroconversion in bivariate generalized estimating equation analysis [63]. However, the association was no longer statistically significant in multivariate analysis after controlling for demographic characteristics and sexual risk behavior (aOR, 1.82 [95% CI, .61–5.44]). Studies suggest an association between MG and HIV, but further evaluation of potential mechanisms is needed.

### Human Papillomavirus

In 2 studies of women with HPV, MG prevalence was 1.5% (10/668) [84] and 1.3% (3/231) [106]. Several studies further evaluated associations of MG infection with HPV infection, specifically high-risk HPV (hr-HPV) infection or cervical interepithelial neoplasia (CIN). Most studies found no statistically signification association between MG and HPV infections [107–109]. One study in pregnant women in Ethiopia found that MG was associated with HPV (6/257 [2.3%] vs 2/522 [0.4%]; *P* = .02) but not with hr-HPV [110]. All other studies also found that MG was not associated with hr-HPV [58, 107, 111, 112]. Two studies did not find an association between the severity of cervical lesions and MG infection, but both studies had relatively low numbers of MG infection, with only 16 and 3 coinfections noted [106, 113]. A study from China found that prevalence of MG in those with CIN and those without CIN was 4.1% (5/121) versus 0.9% (5/547) (OR, 4.207 [95% CI, 1.160–15.260]; *P* = .029), suggesting that the risk of CIN was higher in those with MG infection [84]. Few studies evaluated rectal HPV and MG. In a study of MSM in Togo, hr-HPV anal infection was associated with MG anal infection in a multivariate model (aOR, 9.61 [95% CI, 3.08–29.89]) [21]. Data are inconclusive about an association of MG infection with HPV and associated neoplasia, with some studies demonstrating a statistically significant association and others not finding an association. Further prospective studies are needed to evaluate whether MG infection is a factor in HPV susceptibility and progression.

### Other Infections Associated With MG

While most studies focused on the coinfections described in more detail above, a few other infections were noted. Two studies reported on MG prevalence (8.6% and 15.6%) in those with mpox [114, 115]. One study in female sex workers found an association between Epstein–Barr virus DNA and MG coinfection [116]. Another study found no association between genital schistosomiasis and MG coinfection in women [117]. Consideration of these coinfections should be made in the appropriate populations.

## DISCUSSION

The extensive MG literature published between 2015 and 2025 has greatly increased our understanding of the risk factors for MG infection and its common coinfections and associated clinical manifestations. We found that MG prevalence increases with factors associated with other STIs, including increasing number of sex partners. Societal factors like systemic racism were also associated with increased risk as MG prevalence was higher in individuals with Black race in the US and UK. Infection is common in GU and rectal sites, but uncommon in the pharynx. Coinfections were common. Asymptomatic presentations—particularly at extragenital sites—are ubiquitous. Urethritis, cervicitis, and PID are the most common presentations, but MG infection may be identified in a variety of other disease states and case-reportable events like Fitz-Hugh–Curtis syndrome, neonatal conjunctivitis, and even infective endocarditis have been described, though causation is not definitive in these cases. Further research is needed to understand its potential role in infertility and as a potential risk factor for HIV transmission.

While this narrative review highlighted the most relevant findings from >400 studies, there were limitations to our search strategy. Our search was limited only to PubMed and included only articles published in English. However, use of PubMed for the search likely captured most of the relevant articles given the narrative reviews’ focus on clinical epidemiology, presentations, and coinfections.

While our extensive review of the literature surrounding prevalence, clinical syndromes and presentations, coinfections, and complications of MG highlights many important findings in the last decade, it also brings to light several opportunities for further research to advance care provided to those diagnosed with MG.

First, we lack data on general population prevalence. Healthcare infrastructure and surveillance systems must be leveraged or created to understand the prevalence in diverse communities throughout the world. Second, there is a need for longitudinal, prospective, natural history studies of patients with MG infection, including those with asymptomatic colonization. Rates of asymptomatic MG were significant in our review and there is a need to understand potential complications or risks associated with a prolonged, asymptomatic MG colonization. Third, implementation work is needed to support provider testing for MG in the setting of an appropriate clinical syndrome. MG is likely underdiagnosed in clinical practice as, while co-occurrence with other STIs is common, testing in those with another STI is not. Extragenital MG is likely also vastly underdiagnosed and its clinical significance remains unclear. Systematic development of strategies to enhance provider knowledge and comfort with managing patients with MG is needed.

## Supplementary Material

ofaf799_Supplementary_Data
