# Supplementary material for: Clinical Manifestations and Associated Disease States with Mycoplasma genitalium Infection: Narrative Review and State of the Literature, 2015–2025
Source: Open Forum Infect Dis. 2026 Mar 30;13(Suppl 2):S1339–51. doi: 10.1093/ofid/ofaf799 (PMC13035034; doi:10.1093/ofid/ofaf799)
Supplement: ofaf799_Supplementary_Data [file ofaf799_supplementary_data.zip › Mgen clinical syndrome table_1016.docx]

| **Supplemental Table 2. Clinical Syndromes and Complications of *Mycoplasma Genitalium* Infection** | | | |
| --- | --- | --- | --- |
| **Author, year** | **Country** | **Population** | **Findings** |
| **Asymptomatic infection** | | | |
| Getman, 2016[1] | United States | Symptomatic and asymptomatic males and females | Patients with symptoms more likely to test positive for MG relative to asymptomatic, (OR 2.83, p<0.001). Symptomatic subjects had higher prevalence rates of MG infections (symptomatic versus asymptomatic: female, 21.1% versus 7.5%; male, 19.3% versus 15.4%) |
| Lefebvre, 2017[2] | France | Male and female patients attending the STI clinic | Positive MG test, associated with symptoms aOR 5.3 (95% CI 1.6 to 17.4, p=0.005) |
| Stewart, 2020[3] | Australia | Any patient being tested for CT/NG | Total 56 with MG, MG was detected in five men with epididyo-orchitis and one with urethritis, and in three women with pelvic inflammatory disease, all without other diagnosed sexually transmitted infections. |
| Manhart, 2020[4] | United State | Sexually active men and women | Women who reported an abnormal vaginal odor (OR = 1.82; 95% CI = 1.31 to 2.52) and an abnormal vaginal discharge (OR = 1.67; 95% CI = 1.22 to 2.28) were significantly more likely than women who did not report each symptom to have MG infection. Penile or urethral discharge was the only symptom significantly associated with MG infection among men (OR = 2.77; 95% CI = 1.94 to 3.94). Symptomatic persons were more likely to have MG infection than asymptomatic persons (for women, OR = 1.53 (95% CI = 1.09 to 2.14); for men, OR = 1.42 (95% CI = 1.02 to 1.99) |
| Crowell, 2020[5] | Nigeria | MSM and TGW | The prevalence of urogenital MG among participants reporting one or more symptoms was 11.8% (95% CI 4.1 to 19.4%) and among those reporting no symptoms was 12.6% (95% CI 9.0 to 16.1%, p=0.85). The prevalence of anorectal MG among participants reporting one or more symptoms was 35.4% (95% CI 23.8 to 47.0%) and among those reporting no symptoms was 37.0% (95% CI 31.9 to 42.1%, p=0.80). |
| Karim, 2021[6] | Morocco | Women presenting for routine OB/GYN exam | All 8 patients with MG had symptoms |
| Maina, 2021[7] | Kenya | Patients seeking care for STIs | All MG in males symptomatic, 22.2% of MG in females asymptomatic |
| Stafford, 2021[8] | United States | Pregnant women | Seven women with MG were symptomatic (18%) compared with 78 women who tested negative for M. genitalium infection (11.8%; p=0.31) |
| Alamon-Reig, 2022[9] | Spain | Patients diagnosed with MG | 73/196 had symptoms (37.3%), 119/196 (60.7%) symptoms absent, missing data 4/196 (2%) |
| Che, 2022[10] | China | Women seen at an academic medical center | 35.9% asymptomatic of the 119 with MG |
| Herms, 2022[11] | France | Patients tested for MG at an STI unit | 11% (3/27) with MG and clinical data available were asymptomatic. MG positive patients had significantly more urethral or vaginal discharge than MG negative patients in the univariate analysis (OR = 2.88, 95%CI = 1.25–6.63, P = 0.01). |
| Sandri, 2023[12] | Italy | MSM | 77.1% (74/96) subjects were asymptomatic while 14.6% (14/96) reported signs and symptoms potentially related to gonococcal, chlamydial or MG infection at genital and/or anorectal site |
| Kirkoyun Uysal, 2023[13] | Turkey | Patients seen at university hospital with urogenital symptoms- recruited so that gender was matched- equal females and males recruited | 14/28 with urethritis, 11/28 with vaginitis, 3/28 with cervicitis, p-value 0.32 |
| Sienkiewicz, 2023[14] | United States | University students seeking STI care | MG rates among asymptomatic (6.0%) and symptomatic (7.6%) females were not significantly different (p= 0.28). |
| Manhart, 2023[15] | United States | Persons tested for CT and NG at a sexual health clinic | MG was detected in over one-quarter (26.8%) of males with urethritis, and significantly associated with urethritis (aPR: 1.7; 1.22 to 2.50); MG was detected in 21.1% with vaginitis, 11.8% with cervicitis, 15.4% with PID, and 15.2% with no diagnosed syndrome. No clinical characteristics were associated with female MG infection in adjusted analyses |
| Kadylak, 2024[16] | Poland | Males seeking care at STI clinic | Only 22.2% of genital MG infections were symptomatic, while all extragenital infections were asymptomatic. No significant correlation between the presence of MG and a symptomatic presentation- 16.7% (2/12) with MG with symptoms and 15% (19/127) negative for MG with symptoms, p=0.88) |
| Schröder, 2025[17] | Zambia | Pregnant females | 9.4% (12/127) of those with MG had genitourinary symptoms, not statistically significantly different from those without MG (6.1%, 53/874), p=0.15 |
| Johnson, 2025[18] | United Kingdom | Patients with MG | Urethritis was the most common clinical indication for testing (63.8%, 217/340); cervicitis (3.8%, 13/340), PID (4.1%, 14/340) and proctitis (0.3%, 1/340). Other indications (16.8%, 57/340), Contacts of MG infections made up 11.2% (38/340) |
| **Urethritis** | | | |
| Guschin, 2015[19] | Russia | Male patients at an STI clinic | 85% (40/47) with only MG infection had urethritis symptoms |
| Frølund, 2016[20] | Sweden | Males reporting dysuria or observed urethral discharge with >10 pmns/hpf and controls if no urethritis symptoms and <5 pmns/hpf | MG was associated with acute and chronic NGU (both p < 0.0001) in univariate analysis. Patients with symptomatic NGU (acute and chronic) had higher bacterial loads (median 1.9 × 104 geq/ml in acute NGU and 1.6 × 104 geq/ml in chronic NGU) than the controls (median 44 geq/ml) (p = 0.007 and p = 0.04, Mann-Whitney) |
| You, 2016[21] | Japan | Symptomatic male patients with 5 or more PMNs/hpf | In a clone library of bacterial flora in urine of male patients with symptomatic urethritis, MG found in 5/38 samples analyzed successfully, and it was the predominant organism in 3 specimens |
| le Roux, 2017[22] | South Africa | A group of men with symptoms and/or signs of urethritis and a group of men without urethritis signs and symptoms | MG was detected more in the symptomatic group (13.7%, 41/300) than the asymptomatic group (5.3%, 4/75) ( p = 0.002). Among the symptomatic patients, MG was detected in 17 (18.0%) of the 94 patients with visible urethral discharge and in 24 (11.7%) of the 206 patients with burning on urination. There were significant differences between median bacterial loads of the discharge, burning on urination and asymptomatic groups (p < 0.01) and between the symptomatic and asymptomatic groups ( p = 0.002). |
| Moi H, 2017[23] | Norway | Men attending STI clinic in Oslo | Of all MG infections 488 of 584 (83.6%; 95% CI, 80.6–86.6) had moderate to severe urethritis. |
| Libois, 2018[24] | Belgium | Men with symptoms of urethritis (dysuria, urethral discomfort and/or urethral discharge) | 9% (17/187) tested positive for MG |
| Chra, 2018[25] | Greece | Symptomatic and asymptomatic adult males and females seeking to exclude an STD | Among the MG infected males, 83.3% (5/6) had microscopic findings, symptoms and signs of urethritis. Three of those were MG mono-infected (without coinfection). All of the MG infected females (4/4) were symptomatic, 75% (3/4) displayed symptoms and signs of cervicitis, and 25% (1/4) had symptoms, signs and microscopic findings of cervicitis. |
| Chambers, 2019[26] | United States | Patients at an STI clinic assigned male sex at birth with exclusively male or exclusively female sex partners in the past year. | 20% (49/244) with NGU positive for MG versus 3% (6/188) with no NGU tested positive for MG, p <0.0005 |
| Chambers, 2019[27] | United States | STI clinic patients with NGU | 22/103 = 21% had MG (without CT). 95% urethral symptoms, 57% urethral discharge, 76% dysuria, 91% visible clear urethral discharge. |
| Jordan, 2020[28] | United States | Men with NGU (n=155) and men without NGU (n=103) as controls | 23% (35/155) of those with NGU had MG infections (26/35 were infections with MG alone). Comparing cases and controls, infections with CT and MG alone and mixed infections were significantly associated with NGU (p<0.0001, p=0.0006 and p=0.021, respectively). |
| Jordan, 2020[29] | United States | Symptomatic men with NGU | 18% (39/220) had MG only, >90% had penile discharge, <20% had dysuria, <40% had burning/tingling, <10% had itching. >50% had small amount of discharge, and >70% with discharge reported clear discharge |
| Li, 2020[30] | China | 1816 male subjects were enrolled in a cohort study of gonococcal transmission | 19.7% (358/1816, 95% CI 17.9-19.7) prevalence. All 166 subjects with single MG infection had microscopic evidence of urethral inflammation, judged by PMN counts on Gram stains of urethral exudates (≥1 PMNs/HPF). |
| Rowlinson, 2021[31] | United States | Males | 5.0% (10/200) MG among non-NGU and 22.4% (24/107) MG with NGU p <0.01. |
| Xie, 2021[32] | China | Female patients diagnosed with chronic papillary urethritis who had undergone transuretheral electroresection | 25.2% of patients [37/147] with chronic papillary urethritis found to be positive for MG |
| de Souza, 2021[33] | Brazil | Males with urethral discharge | 6.5% (11/170) with MG among all with male urethritis in this study (5/11 were mono-infections) |
| Lee, 2022[34] | Hong Kong | Males presenting with NGU | Higher prevalence of MG (vs MG negative) in patients presenting with urethral discharge (12% vs 6%, OR 2.16, 95% CI 1.10–4.23; P=0.02) and patients with symptom duration >2 weeks (14% vs 6%, OR 2.34, 95% CI 1.10–4.97; P=0.03) |
| Llaca-Díaz, 2023[35] | Mexico | Males urology patients with symptoms of urethritis | 4/200 with urethritis with MG, no statistically significant association with MG infection and burning with urination (OR 0.26, 0.02-3.16), testicular inflammation (OR 1.29, 0.38-7.94), penile discharge (OR 0.99, 0.33-5.67), genital warts (OR 0.23, 0.03-5.92), blisters or sores (OR 0.78, 0.09-3.99) |
| Wang, 2024[36] | China | Individuals attending STI-related departments including dermatology, urology, gynecology, obstetrics | In adjusted models, MG was associated with uretheral serous discharge (aOR 2.99, 95% CI 1.41-6.34, p-value 0.004), uretheral purulent discharge (aOR 2.88, 95% CI 1.20-6.91, p-value 0.018), uretheral burning or irritation (aOR 1.92, 95% CI 1.08-3.43, p-value 0.027) |
| **Cervicitis** | | | |
| Lusk, 2016[37] | Australia | Women at STI clinic reporting symptoms (60%) or requesting vaginal exam | MG was associated with cervicitis (defined as microscopy with >30 pmns/hpf and yellow and/or mucopurulent discharge, adjusted prevalence ratio: MG aPR =2.21 (95% CI 1.33 to 3.69) p=0.002 |
| Dehon, 2016[38] | United States | Women living with HIV on antiretroviral treatment | Cytokines elevated among 3 women with chronic MG compared to 4 who never tested positive and normalized after treatment for MG. Women with chronic MG had higher leukocyte to epithelial cell ratios compared to those who never tested positive, which normalized after cure. |
| Chirenje, 2018[39] | Zimbabwe | 200 women aged 18–53 years presenting with vaginal discharge at the clinic | 14/200 = 7% tested positive for MG, 11 were MG alone |
| Taylor, 2018[40] | United States | Women aged 15-35 with mucopurulent cervicitis, diagnosis of chlamydial infection prior to treatment or reported sexual contact with an individual with a recent diagnosis of chlamydial urethritis or non-gonococcal urethritis, PID excluded. | Only 28% of women with MG infection reported symptoms. 48.7% of MG positive women had endometrial infection (diagnosed on biopsy). |
| Lillis, 2019[41] | United States | Non-pregnant women aged ≥18 | Among 117 women without CT, NG, TV, or BV, trend for MG predicting cervicitis (aOR 3.18, 0.99-10.2, p=0.05), but not vaginal discharge, itching, burning, or PID |
| Hoffman, 2019[42] | South Africa | Women (≥18 years) who attended the mobile clinic for any health services. | Vaginal discharge was significantly associated with MG (OR, 3.5; 95% CI, 1.3–9.3) |
| Lapii, 2019[43] | Russia | Women who had urethral polyps removed | 8.9% of 90 women with urethral polyps tested positive for MG (no control group) |
| Güralp, 2019[44] | Turkey | Women with or without symptoms | Among women with cervicitis, MG was significantly more frequent in women with cervicitis than in those without cervicitis (5.6% vs. 1.2% p<0.005) |
| Roy, 2021[45] | India | 150 patients with cervicitis (visible yellow mucous and/or contact bleeding or >=30 PMNLs/ 1000x filed on gram stain of cervical mucus) and 150 healthy controls (patients seeking contraception or routine follow-up after pregnancy) | 24.6% (37/150) of patients with cervicitis positive for MG, 0% (0/150) of healthy controls positive for MG. As a single organism MG was not statistically significantly associated with cervicitis (17/150, 11.3% of patients in cervicitis patients, 0/150, 0% of patients without cervicitis, p >0.05) |
| Che, 2022[10] | China | Females | 37% (34/92) with MG positive and CT negative (MG+/CT-) had >15 WBCs vs 22.8% (114/500) of those negative for MG and CT, p-value <0.05. In patients MG positive and CT positive 74.1% (20/27) with > 15 WBCs, p<0.05 when compared to all other groups suggesting co-infection of MG and CT can increase of WBCs, compared to MG or CT infection alone. |
| Wang, 2022[46] | United States | Adolescent and young adult women | MG infection not associated with vaginal WBCs being present, PR 1.19 (95% CI, 0.40-3.56). Cytokine, chemokine, or antimicrobial protein concentrations from those with MG (n=17) did not differ from participants who did not have MG infection (p>0.05). Among the 17 women with MG infection, the concentration of secretory leukocyte proteinase inhibitor was higher in women with genitourinary symptoms (p= 0.03) than women without genitourinary symptoms. There was no difference in cytokine concentration between women with MG who 1) did and did not have vaginal WBCs (p> 0.05), or 2) had at least one other STI or only had MG (p> 0.05). |
| Latimer, 2022[47] | Australia | Women | In women without BV, MG was strongly associated with cervicitis (AOR=4.38, 95% CI: 1.69 to 11.33, p=0.002), but this association was not found in women with BV. MG was not associated with any other clinical signs, including vaginal PMNL count; although all women with MG cervicitis had ≥5 PMNL/hpf detected. |
| **Pelvic Inflammatory Disease** | | | |
| Hay, 2016[48] | United Kingdom | Young women at universities | After adjustment for baseline chlamydia infection, PID was not associated with baseline MG (aRR 2.90, 95% CI 0.89-9.44) |
| Oliphant, 2016[49] | New Zealand | Women presenting for STI testing | No association with MG and PID found (OR 0.91; 95% CI 0.38-2.20) |
| Ong, 2017[50] | Australia | Matched male-female partner pairs attending clinic on the same day | Female sexual partners of men diagnosed with pathogen-negative NGU had an increased odds (AOR, 4.8; 95% CI, 2.1–11.3) of being diagnosed with PID |
| Latimer, 2019[51] | Australia | Women were included if they were 18 years or older, PID presumptively diagnosed using CDC criteria. | 149 records of women with MG-PID were identified, and MG accounted for 5.5% of PID cases. Women with MG-PID were more likely to have lower abdominal tenderness (adjusted OR, 2.29; 95% CI, 1.14–4.60), but less likely to have a modest elevation in vaginal PMN counts, compared to women with CT-PID. |
| Trent, 2021[52] | United States | Females 13-25 years of age with mild to moderate PID | MG positive in 45% (24/54) of women with PID |
| Sweeney, 2022[53] | Australia | Patients with PID seen at 5 family planning sites | 72 PID cases included in analysis, of those 16 were STI pathogen related and of those 6 were MG positive (38%), 5 MG alone and 1 MG and CT. |
| Menezes, 2023[54] | United States | Females aged 13-20 who were testing for CT and NG | Of MG positive participants, 13/17 (76%) were asymptomatic on the day of testing; Of the 56 participants who had a bimanual pelvic exam, a higher proportion,40% (2/5) with cervical motion/adnexal tenderness had MG vs.7.8% (4/51) without cervical motion/adnexal tenderness, p= 0.027. Both participants with cervical motion/adnexal tenderness and MG were co-infected with another STI pathogen. |
| **Proctitis** | | | |
| Bissessor, 2016[55] | Australia | Men with symptomatic proctitis | Symptom profile: anal discharge (7/18), rectal pain (4/18), or both (7/18). Significantly higher bacterial load compared to men with MG on rectal swab but no symptoms. |
| Ong, 2018[56] | Australia | MSM diagnosed with clinical diagnosis of proctitis | 31% (95% CI 25-38%, 66/212) positive for MG, including 17% (95% CI 12-24%, 29/166) with only MG infection |
| Foschi, 2018[57] | Italy | MSM | 4.8% (8/165) with MG at the rectal site. All cases were found to be asymptomatic and characterized by the presence of low bacterial DNA. |
| Mwasakifwa, 2020[58] | Australia | Gay, bisexual men, and transgender women diagnosed with proctitis | 67.3% (76/113) with MG among those with documented proctoscopy findings. 6.8% (10/147) with documented proctitis had MG has only identified organism |
| Chow, 2021[59] | Australia | Two groups of MSM 1) men presenting with symptomatic proctitis and 2) asymptomatic men not reporting symptoms of proctitis | MG was more commonly detected among men with proctitis (9.4%; 95% CI, 7.0%–12.3%; 47/499) compared with asymptomatic men (5.1%; 95% CI, 3.4%–7.4%; 26/506; P = .010), with a risk difference of 4.3% (95% CI, 1.1%–7.5%). the most common symptoms reported were anorectal pain (n = 38, 81%), anal bleeding (n = 18, 38%), anal discharge (n = 13, 28%), and tenesmus (n = 11, 23%). |
| Berti, 2023[60] | France | MSM with anoproctitis | Less likely MG (11% (36/319) vs 2% (1/46), p=0.01) if presented with anal fissure. No statistically significant difference between those presenting without MG and those with MG in % presenting with following clinical symptoms- ulcerations (37% (118/319) vs 28% (13/46), p=0.25), erythematous proctitis (39% (123/319) vs 43% (20/46), p=0.52), purulent proctitis (29% (93/319) vs 24% (11/46), p=0.46), fever (6% (19/319) vs 7% (3/46), p=0.75), abscess (11% (36/319) vs 11% (5/46), p=0.20). |
| Baiers, 2024[61] | United States | Young adult sexual and gender minorities assigned male at birth | 6.3% (n=79) patients with rectal swab positive for MG had any rectal symptoms. No differences in prevalence of individual symptoms by pattern of bacterial STI results (P > .05). |
| **Prostatitis** | | | |
| Mo, 2016[62] | China | Patients with prostatitis cared for by the same urologist | Prevalence of MG 10% in those with prostatitis vs 3% in the control group, (p=0.005). |
| Miyake, 2019[63] | Japan | Patients with prostate cancer who underwent robot-assisted laparoscopic radical prostatectomy (n=45) or with benign prostatic hyperplasia who underwent TURP (n=33). | 40% (18/45) with MG among those with prostate cancer cohort and 18% with MG (6/33) in those undergoing TURP, p=0.03 |
| **Other infections associated with *M. Genitalium*** | | | |
| Justel, 2015[64] | Angola | Pregnant women | Two cases of neonatal conjunctivitis identified from mother-to-child transmission |
| Oeser, 2020[65] | United Kingdom | Neonates <72 hours undergoing investigation for presumed sepsis | 0/208 with MG |
| Towns, 2021[66] | Australia | Woman in her 30s. | Case report of Fitz-Hugh-Curtis Syndrome associated with MG. |
| Ramakrishnan, 2023[67] | Switzerland | 42 year old woman | Case report of a 42 year old woman with history of systemic lupus erythematous (on azathioprine) and mechanical aortic valve diagnosed with prosthetic valve endocarditis with 16s sequencing on excised heart valve identifying MG with 100% match. |
| Basu, 2025[68] | India | 33 year old male patient | Case report of reactive arthritis after anogenital MG infection- associated with erythematous plaques on body, keratoderma of soles and fixed flexion deformities in multiple joints |
| **Infertility** | | | |
| *Female infertility* | | | |
| Rajkumari, 2015[69] | India | Women undergoing infertility work up | MG in 15/94 cases. Tubal occlusion was seen in 5 (33.3%) women with MG infection, and none of the women negative for MG infection had tubal occlusion (p < 0.001). |
| Rekha, 2019[70] | India | Infertile women in the age group 22–40 years (n=162) and controls presenting for c-section (n=162) | MG positive in 6.1% (10/162) among infertile group and 1/162 = 0.6% (1/162) among controls. MG was more common in women with unexplained infertility (13.3%) versus those with an underlying cause identified (4.5%), p<0.05. |
| Lokken, 2023[71] | Kenya | HIV negative women trying to conceive | MG infection at the visit prior to pregnancy testing was associated with a 19% lower fecundability in unadjusted analysis (FR 0.81, 95% CI 0.48–1.36); After adjustment for age, frequency of condomless sex in the last 4 weeks, and study site, MG was associated with a 27% lower fecundability, but confidence intervals remained wide (adjusted FR 0.73, 95% CI 0.44–1.23). |
| Ling, 2022[72] | China | Women aged 21-43 seeking surgical treatment for infertility | Total infection rate in women with infertility was 8.1% (18/223), MgG associated with tubal obstruction (OR 3.886, p=0.008) but not associated with tubal adhesion, endometriosis or endometrial polyps |
| Sameni, 2022[73] | Iran | 54 pregnant women and 65 infertile women | 11/65 (16.9%) MG positive in infertile group, 3/54 (5.5%) in pregnant women with MG |
| Mbah, 2022[74] | Cameroon | Cases were women with secondary infertility, controls were randomly selected pregnant women seen in prenatal clinic | 6% women with secondary infertility positive for MG (9/151) vs 7.1% (17/265) in controls (pregnant women), p= 0.68. compared to MG negative, MG positive with Odds of secondary infertility in those positive for MG relative to negative for MG was 0.84 (95% CI0.36–1.94, p=0.69). |
| Krotik, 2021[75] | Ukraine | Infertile women and those without history of infertility planning pregnancy | MG in infertile women 19.8% (19/96) vs 3.3% (1/30) of women planning pregnancy without history of infertility, p-value <0.01 |
| *Male infertility* | | | |
| Qing, 2017[76] | China | Males reporting infertility | MG found in 2% (51/2607). Patients positive for MG tended to have higher sperm DNA fragmentation index (DFI) than those without MG (25.29 ± 15.70 versus 19.01 ± 12.80, p = 0.03). |
| Kim, 2017[77] | South Korea | 400 men undergoing a fertility evaluation | 1.3% (5/381) positive for MG. Sperm quality for MG infected groups were not significantly different from those in the corresponding non-infected groups (but small numbers) |
| Ahmadi, 2018[78] | Iran | Asymptomatic male patients with abnormal semen analysis at baseline | 9.7% (16/165) of infertile men tested positive for MG, and just 2 (1.2%) of control group (p=0.001, OR 8.8, CI 1.9-38.7). There was a significantly higher organism load in the infertile group, and statistically significant improvement in semen parameters after antibiotic treatment |
| Akgul, 2018[79] | Turkey | Patients between 21 and 55 years that presented to infertility clinic. Cases had leucocyte count in sperm over 1 million/ml and the control group was asymptomatic with leucocyte count below 1 million/ml in semen analysis. | 0/25 in case group and 0/25 in control group had MG |
| Li, 2020[80] | China | Male partners from infertile couples (n=30,094) | MG prevalence: (749/30,094, 2.49%; 95% CI, 2.31–2.66%, nearly all asymptomatic. Semen concentration and total sperm count were lower in infected males than in uninfected males. After antibiotic treatment, the mean values of the semen parameters increased from those measured before treatment, especially semen concentration, except for leukocyte concentration. |
| Bai, 2021[81] | China | Males seeking fertility evaluation | 2.1% (4/195) of semen samples positive for MG overall, 4.5% (4/88) of those with MG with leukocytospermia vs 0/107 (0%), p=0.04 |
| Moridi, 2021[82] | Iran | Males seeking fertility evaluation | 0/100 positive for MG |
| Tjagur, 2021[83] | Estonia | Study group was 2000 males with fertility problems or desire for fertility check and control group was 258 male partners of pregnant women | 1.1% (22/2000) infertile men positive for MG vs 0% (0/248) of control group (fertile men) with MG, p=0.16. Semen neutrophils and IL-6 concentration both statistically higher in patients with MG compared to STI-negative patients. No statistically significant differences in volume of seminal fluid, concentration of spermatozoa, total count of spermatozoa, spermatozoa with normal morphology and spermatozoa with progressive motility. |
| Bai, 2022[84] | China | Males seeking fertility evaluation | 2.7% (2/73) men with primary infertility with MG, 1.7% (1/60) men with secondary infertility, p=0.99 |
| *Couples with infertility* | | | |
| Al-Masri, 2022[85] | Palestine | Patients with infertility | Found in 16 (7.3%) specimens- 2 (12.5%) vaginal swabs, 2 (5.4%) urines, 4 (7%) semen; overall prevalence MG (4.9%) |
| Tam Le, 2022[86] | Vietnam | Infertile couples | 3/380 MG positive in semen samples. Sperm motility and sperm vitality in the Mycoplasma-positive group were significantly lower than those in the Mycoplasma-negative group (sperm motility: 7.33 ± 12.70 vs. 29.11 ± 14.68, p = 0.02; and sperm vitality: 23.00 ± 39.84 vs. 71.75 ± 22.46, p < 0.001). The other indices were not significantly different between the two groups. The sperm concentration, mean sperm volume, and mean proportion of normal sperm morphology in the Mycoplasma-positive group were 11.33 ± 19.63, 1.50 ± 0.50 mL, and 1.00 ± 1.73%, respectively; these parameters in the Mycoplasma-negative group were higher than those in the positive group, at 30.70 ± 16.14, 1.73 ± 0.97 mL and 4.07 ± 5.22%, respectively, but the differences were not significant. |
| Ahmadi, 2023[87] | Iran | 50 infertile couples and 50 fertile couples | 0/50 with MG in semen specimens from infertile men and 0/50 positive for MG in fertile men. 4/50 positive for MG in infertile women's endocervical swabs, 0/50 in fertile women. As 0 MG infections in males unable to look at sperm parameters. |
| **Pregnancy Complications** | | | |
| Ashshi, 2015[88] | Saudia Arabia | Fallopian tube specimens from 84 women with ectopic pregnancy (cases) and 31 women during tubal ligation (controls) | MG was associated with ectopic pregnancy, MG (OR 2.3; 95 % CI: 1.1 – 8.6; P=0.03) |
| Cox, 2016[89] | United Kingdom | Placental specimens of women at or before 37 weeks gestation | 24/57 specimens with chorioamnionitis. MG not found in any specimens. |
| Refaat, 2016[90] | Saudi Arabia | 96 patients with ectopic pregnancy and 61 controls during total abdominal hysterectomy | MG was more likely to be found in ectopic pregnancy than total abdominal hysterectomy groups, crude OR = MG (COR 5.7, 95% CI 1.3–23.7; P=0.006) |
| Ramazanzadeh, 2016[91] | Iran | Women with spontaneous abortion (cases) and women with normal pregnancy between 20 and 37 weeks (controls) | The prevalence of MG was 0.9 % in those with spontaneous abortion and 2.8 % in those with normal pregnancy |
| Rowlands, 2017[92] | Australia | Women undergoing amniocentesis for genetic prenatal diagnosis between 15 and 22 weeks gestation | 0/344 with MG and no evidence of microbial invasion of healthy amniotic cavity |
| Contini, 2018[93] | Italy | Females with spontaneous abortion as cases (n = 100); and females who underwent voluntary interruption of pregnancy (n = 100) as controls. All with gestational age <12 weeks | MG found in 0/100 cases and 0/100 controls |
| Kayem, 2018[94] | France | Women with a singleton pregnancy who underwent amniocentesis from 16 to 20 weeks for Down syndrome screening. | 1.3% (13/1016) of amniotic fluid was positive for MG. Bacterial colonization of amniotic fluid rare in second trimester. |
| Perin, 2021[95] | United States | Pregnant women aged 13 to 29 years old | Preterm delivery was higher among women with MG compared to women without MG after adjusting for age, race, education, and risk of STIs (aOR 2.33, 95% CI 1.24 – 4.37). When excluding women with other STI co-diagnoses from those with MG, this association with preterm birth was not maintained (aOR 1.50, 0.73 – 3.10). Gestational age at delivery was similar for women with MG diagnoses compared to women with no STIs (38 weeks and 5 days vs 38 weeks and 4 days respectively). Average birth weight tended to be lower for women with MG than those with no STIs, with an average adjusted difference of 202g, 59 to 345g. The average adjusted reduction in birth weight for infants with maternal MG diagnosis compared to infants with no STI after excluding those with MG treatment was reduced from 169 to 34 grams (95% CI -118 to 186). |
| Juliana, 2022[96] | Tanzania | Pregnant females | Only 3/82 specimens (3.7%) of samples taken during pregnancy but after 20 weeks were positive for MG, 0/44 during pregnancy and < 20 weeks, 0/44 after delivery. All 3 positive were community state type III which is Lactobacillis iners dominant. |
| Hu, 2023[97] | United States | Pregnant women seen in OB/GYN clinics | MG was detected at a higher frequency in patients with an outcome of preterm birth (11.4% vs. 7.8%), small for gestational age (14.8% vs. 7.7%), or fetal growth restriction (15.4% vs. 8.2%) but was not significantly associated with these outcomes. |
| Govender, 2024[98] | South Africa | HIV negative pregnant women | Among those positive for MG at baseline: 81.3% (13/16) with term birth, 18.8% (3/16) with preterm birth relative to those negative for MG: 89.9% (338/377) with term birth, 10.1% (39/377) with preterm birth (p= 0.22). Low birth weight was significantly associated with testing positive for MG (OR 5.22; 95% CI: 1.10 to 15.98) |
| Scoullar, 2024[99] | Papau New Guinea | Pregnant women | MG infection was associated with a lower mean birthweight of 166.9 g (95% CI: 324.2 g lower to 9.7 g lower, p = 0.038) compared to uninfected women |
| Nandagopal, 2024[100] | India | Patients with abnormal bleeding or reproductive failure cases under treatment or confirmed diagnosis of chronic endometritis | MG present in 2/116 (1.7%) with abnormal bleeding, 9.4% (19/203) with recurrent implantation failure, and 5.5% (10/181) with recurrent pregnancy loss |

Abbreviations: MG: Mycoplasma genitalium; OR: Odds Ratio; aOR: adjusted Odds Ratio; CI: confidence interval; CT: Chlamydia Trachomatis; NG: Neisserria gonorrhea; MSM: Men who have sex with men; TGW: trans-gender women; OB/GYN: Obstetrics and gynecology; STI: sexually transmitted infection; PID: Pelvic Inflammatory Disease; pmns: polymorphonuclear neutrophils; hpf: high power field; NGU: non-gonococcal urethritis; vs: versus; PR; prevalence ratio; aPR: adjusted prevalence ratio; HIV: Human immunodeficiency virus; TV: Trichomonas vagnialis; BV: Bacterial vaginosis; PMNL: polymorphonuculear leukocyte; CDC: Centers for Disease Control; DNA: deoxyribonucleic acid; TURP: transurethral resection of the prostate; IL-6: interleukin 6

References:

1. Getman D, Jiang A, O’Donnell M, Cohen S. Mycoplasma genitalium Prevalence, Coinfection, and Macrolide Antibiotic Resistance Frequency in a Multicenter Clinical Study Cohort in the United States. J Clin Microbiol **2016**; 54:2278–2283.

2. Lefebvre M, Coutherut J, Gibaud S, et al. Prevalence of Mycoplasma genitalium Infection and Relationship with Symptoms Among Adults Attending a Sexual Health Centre. Acta Derm Venereol **2017**; 97:543–545.

3. Stewart JD, Webb B, Francis M, Graham M, Korman TM. Should we routinely test for Mycoplasma genitalium when testing for other sexually transmitted infections? Med J Aust **2020**; 212:30–31.

4. Manhart LE, Gaydos CA, Taylor SN, et al. Characteristics of Mycoplasma genitalium Urogenital Infections in a Diverse Patient Sample from the United States: Results from the Aptima Mycoplasma genitalium Evaluation Study (AMES). J Clin Microbiol **2020**; 58:e00165-20.

5. Crowell TA, Lawlor J, Lombardi K, et al. Anorectal and Urogenital Mycoplasma genitalium in Nigerian Men Who Have Sex With Men and Transgender Women: Prevalence, Incidence, and Association With HIV. Sex Transm Dis **2020**; 47:202–206.

6. Karim S, Bouchikhi C, Banani A, et al. Bacterial sexually transmitted infections and syndromic approach: a study conducted on women at Moroccan University Hospital. Germs **2021**; 11:544–553.

7. Maina AN, Mureithi MW, Ndemi JK, Revathi G. Diagnostic accuracy of the syndromic management of four STIs among individuals seeking treatment at a health centre in Nairobi, Kenya: a cross-sectional study. Pan Afr Med J **2021**; 40:138.

8. Stafford IA, Hummel K, Dunn JJ, et al. Retrospective analysis of infection and antimicrobial resistance patterns of Mycoplasma genitalium among pregnant women in the southwestern USA. BMJ Open **2021**; 11:e050475.

9. Alamon-Reig F, Riera-Monroig J, González-Cordón A, Bosch J, Alsina M, Fuertes I. Mycoplasma genitalium: A descriptive study of 196 cases. Indian J Dermatol Venereol Leprol **2022**; 88:873.

10. Che G, Liu F, Yang Q, et al. Mycoplasma genitalium and Chlamydia trachomatis infection among women in Southwest China: a retrospective study. Epidemiol Infect **2022**; 150:e129.

11. Herms F, Poizeau F, Anyfantakis V, et al. Mycoplasma genitalium screening in a specialized French unit: A retrospective study. Ann Dermatol Venereol **2022**; 149:165–168.

12. Sandri A, Carelli M, Visentin A, et al. Mycoplasma genitalium antibiotic resistance-associated mutations in genital and extragenital samples from men-who-have-sex-with-men attending a STI clinic in Verona, Italy. Front Cell Infect Microbiol **2023**; 13:1155451.

13. Kirkoyun Uysal H, Koksal MO, Sarsar K, et al. Prevalence of Chlamydia trachomatis, Neisseria gonorrhoeae, and Mycoplasma genitalium among Patients with Urogenital Symptoms in Istanbul. Healthcare (Basel) **2023**; 11:930.

14. Sienkiewicz L, Thomas Y, Reynoso A, Munson E. Incidence and laboratory diagnosis of sexually-transmitted infections among university students in a high-prevalence community. J Am Coll Health **2023**; 71:571–577.

15. Manhart LE, Leipertz G, Soge OO, et al. Mycoplasma genitalium in the US (MyGeniUS): Surveillance Data From Sexual Health Clinics in 4 US Regions. Clin Infect Dis **2023**; 77:1449–1459.

16. Kadylak D, Czarny J, Nowicki RJ, Sokołowska-Wojdyło M. The Prevalence of Sexually Transmitted Infections among Male Patients at a Dermato-Venereology Outpatient Clinic in Gdańsk, Poland: Findings from a Single-Center Study. J Clin Med **2024**; 13:3736.

17. Schröder D, Sorano S, Shipitsyna E, et al. Prevalence and epidemiology of Mycoplasma genitalium and the absence of macrolide resistance in M. genitalium among pregnant women attending antenatal care in Zambia. Front Public Health **2025**; 13:1576376.

18. Johnson K, Buluwela E, McDonald G, et al. Mycoplasma genitalium treatment outcomes among a cohort failing macrolide resistance-guided treatment across three London sexual health clinics. Sex Transm Infect **2025**; 101:5–9.

19. Guschin A, Ryzhikh P, Rumyantseva T, Gomberg M, Unemo M. Treatment efficacy, treatment failures and selection of macrolide resistance in patients with high load of Mycoplasma genitalium during treatment of male urethritis with josamycin. BMC Infect Dis **2015**; 15:40.

20. Frølund M, Lidbrink P, Wikström A, Cowan S, Ahrens P, Jensen JS. Urethritis-associated Pathogens in Urine from Men with Non-gonococcal Urethritis: A Case-control Study. Acta Derm Venereol **2016**; 96:689–694.

21. You C, Hamasuna R, Ogawa M, et al. The first report: An analysis of bacterial flora of the first voided urine specimens of patients with male urethritis using the 16S ribosomal RNA gene-based clone library method. Microb Pathog **2016**; 95:95–100.

22. le Roux MC, Hoosen AA. Quantitative Real-Time Polymerase Chain Reaction for the Diagnosis of Mycoplasma genitalium Infection in South African Men With and Without Symptoms of Urethritis. Sex Transm Dis **2017**; 44:17–20.

23. Moi H, Hartgill U, Skullerud KH, Reponen EJ, Syvertsen L, Moghaddam A. Microscopy of Stained Urethral Smear in Male Urethritis; Which Cutoff Should be Used? Sex Transm Dis **2017**; 44:189–194.

24. Libois A, Hallin M, Crucitti T, Delforge M, De Wit S. Prevalence of Mycoplasma genitalium in men with urethritis in a large public hospital in Brussels, Belgium: An observational, cross-sectional study. PLoS One **2018**; 13:e0196217.

25. Chra P, Papaparaskevas J, Papadogeorgaki E, et al. Prevalence of Mycoplasma genitalium and other sexually-transmitted pathogens among high-risk individuals in Greece. Germs **2018**; 8:12–20.

26. Chambers LC, Morgan JL, Lowens MS, et al. Cross-sectional study of urethral exposures at last sexual episode associated with non-gonococcal urethritis among STD clinic patients. Sex Transm Infect **2019**; 95:212–218.

27. Chambers LC, Hughes JP, Glick SN, et al. Resolution of Symptoms and Resumption of Sex After Diagnosis of Nongonococcal Urethritis Among Men Who Have Sex With Men. Sex Transm Dis **2019**; 46:676–682.

28. Jordan SJ, Toh E, Williams JA, et al. Aetiology and prevalence of mixed-infections and mono-infections in non-gonococcal urethritis in men: a case-control study. Sex Transm Infect **2020**; 96:306–311.

29. Jordan SJ, Toh E, Williams JA, et al. No Pathogen-Specific Sign or Symptom Predicts the Etiology of Monomicrobial Nongonococcal Urethritis in Men. Sex Transm Dis **2020**; 47:329–331.

30. Li Y, Su X, Le W, et al. Mycoplasma genitalium in Symptomatic Male Urethritis: Macrolide Use Is Associated With Increased Resistance. Clin Infect Dis **2020**; 70:805–810.

31. Rowlinson E, Hughes JP, Chambers LC, et al. Incidence of Nongonococcal Urethritis in Men Who Have Sex With Women and Associated Risk Factors. Sex Transm Dis **2021**; 48:341–346.

32. Xie J, Li X, Lü Y, et al. Female chronic posterior urethritis is underestimated in patients with lower urinary tract symptoms. Transl Androl Urol **2021**; 10:3456–3464.

33. de Souza LS, Sardinha JC, Talhari S, Heibel M, Santos MND, Talhari C. Main etiological agents identified in 170 men with urethritis attended at the Fundação Alfredo da Matta, Manaus, Amazonas, Brazil. An Bras Dermatol **2021**; 96:176–183.

34. Lee SS, Cheng KF, Wong NS, et al. Emergence of antibiotic-resistant Mycoplasma genitalium as the cause of non-gonococcal urethritis in male patients at a sexually transmitted infection clinic. Int J Antimicrob Agents **2022**; 59:106510.

35. Llaca-Díaz J, Medina-Loredo V, Huerta-López D, Casillas-Vega N. Sexually Transmitted Infections in Male Patients with Urethritis. Pathogens **2023**; 12:1434.

36. Wang F, Zhang C, Xiu L, et al. Etiological, sociodemographic and clinical characteristics of sexually transmitted infections and M. genitalium resistance in Shenzhen: a multicenter cross-sectional study in China. Front Cell Infect Microbiol **2024**; 14:1407124.

37. Lusk MJ, Garden FL, Rawlinson WD, Naing ZW, Cumming RG, Konecny P. Cervicitis aetiology and case definition: a study in Australian women attending sexually transmitted infection clinics. Sex Transm Infect **2016**; 92:175–181.

38. Dehon PM, Hagensee ME, Sutton KJ, Oddo HE, Nelson N, McGowin CL. Histological Evidence of Chronic Mycoplasma genitalium-Induced Cervicitis in HIV-Infected Women: A Retrospective Cohort Study. J Infect Dis **2016**; 213:1828–1835.

39. Chirenje ZM, Dhibi N, Handsfield HH, et al. The Etiology of Vaginal Discharge Syndrome in Zimbabwe: Results from the Zimbabwe STI Etiology Study. Sex Transm Dis **2018**; 45:422–428.

40. Taylor BD, Zheng X, O’Connell CM, Wiesenfeld HC, Hillier SL, Darville T. Risk factors for Mycoplasma genitalium endometritis and incident infection: a secondary data analysis of the T cell Response Against Chlamydia (TRAC) Study. Sex Transm Infect **2018**; 94:414–420.

41. Lillis RA, Martin DH, Nsuami MJ. Mycoplasma genitalium Infections in Women Attending a Sexually Transmitted Disease Clinic in New Orleans. Clin Infect Dis **2019**; 69:459–465.

42. Hoffman CM, Mbambazela N, Sithole P, et al. Provision of Sexually Transmitted Infection Services in a Mobile Clinic Reveals High Unmet Need in Remote Areas of South Africa: A Cross-sectional Study. Sex Transm Dis **2019**; 46:206–212.

43. Lapii GA, Molodykh OP, Yakovlev AV, Neimark AI, Bakarev MA. Ultrastructural Analysis of Urethral Polyps against the Background of Urogenital Infection. Bull Exp Biol Med **2019**; 167:795–800.

44. Güralp O, Bostancı A, Özerkman Başaran E, Schild-Suhren M, Kaya B. Evaluation of the prevalence of sexually transmitted bacterial pathogens in Northern Cyprus by nucleic acid amplification tests, and investigation of the relationship between these pathogens and cervicitis. Turk J Obstet Gynecol **2019**; 16:242–248.

45. Roy A, Dadwal R, Yadav R, et al. Association of Chlamydia trachomatis, Neisseria gonorrhoeae, Mycoplasma genitalium and Ureaplasma species infection and organism load with cervicitis in north Indian population. Lett Appl Microbiol **2021**; 73:506–514.

46. Wang R, Trent ME, Bream JH, et al. Mycoplasma genitalium Infection Is Not Associated With Genital Tract Inflammation Among Adolescent and Young Adult Women in Baltimore, Maryland. Sex Transm Dis **2022**; 49:139–144.

47. Latimer RL, Vodstrcil LA, Plummer EL, et al. The clinical indications for testing women for Mycoplasma genitalium. Sex Transm Infect **2022**; 98:277–285.

48. Hay PE, Kerry SR, Normansell R, et al. Which sexually active young female students are most at risk of pelvic inflammatory disease? A prospective study. Sex Transm Infect **2016**; 92:63–66.

49. Oliphant J, Azariah S. Pelvic inflammatory disease associated with Chlamydia trachomatis but not Mycoplasma genitalium in New Zealand. Sex Health **2016**; 13:43–48.

50. Ong JJ, Sarumpaet A, Chow EPF, et al. Should Female Partners of Men With Non-Gonococcal Urethritis, Negative for Chlamydia trachomatis and Mycoplasma genitalium, Be Informed and Treated? Clinical Outcomes From a Partner Study of Heterosexual Men With NGU. Sex Transm Dis **2017**; 44:126–130.

51. Latimer RL, Read TRH, Vodstrcil LA, et al. Clinical Features and Therapeutic Response in Women Meeting Criteria for Presumptive Treatment for Pelvic Inflammatory Disease Associated With Mycoplasma genitalium. Sex Transm Dis **2019**; 46:73–79.

52. Trent M, Perin J, Rowell J, et al. Using Innovation to Address Adolescent and Young Adult Health Disparities in Pelvic Inflammatory Disease: Design of the Technology Enhanced Community Health Precision Nursing (TECH-PN) Trial. J Infect Dis **2021**; 224:S145–S151.

53. Sweeney S, Bateson D, Fleming K, Huston W. Factors associated with pelvic inflammatory disease: A case series analysis of family planning clinic data. Womens Health (Lond) **2022**; 18:17455057221112263.

54. Menezes ME, Silver EJ, Goldstein DY, Collins-Ogle MD, Fox AS, Coupey SM. Prevalence and Factors Associated With Mycoplasma genitalium Infection in At-Risk Female Adolescents in Bronx County, New York. Sex Transm Dis **2023**; 50:635–641.

55. Bissessor M, Tabrizi SN, Bradshaw CS, et al. The contribution of Mycoplasma genitalium to the aetiology of sexually acquired infectious proctitis in men who have sex with men. Clin Microbiol Infect **2016**; 22:260–265.

56. Ong JJ, Aung E, Read TRH, et al. Clinical Characteristics of Anorectal Mycoplasma genitalium Infection and Microbial Cure in Men Who Have Sex With Men. Sex Transm Dis **2018**; 45:522–526.

57. Foschi C, Salvo M, D’Antuono A, et al. Distribution of genital Mollicutes in the vaginal ecosystem of women with different clinical conditions. New Microbiol **2018**; 41:225–229.

58. Mwasakifwa GE, Nugent C, Varma R. Proctitis in gay and bisexual men. Are microscopy and proctoscopy worthwhile? Sex Transm Infect **2020**; 96:475–477.

59. Chow EPF, Lee D, Bond S, et al. Nonclassical Pathogens as Causative Agents of Proctitis in Men who Have Sex With Men. Open Forum Infect Dis **2021**; 8:ofab137.

60. Berti V, Blondel J, Spindler L, et al. Infective anoproctitis in men having sex with men: Don’t forget Mycoplasma genitalium. Infect Dis Now **2023**; 53:104771.

61. Baiers RA, Ryan DT, Clifford A, et al. Asymptomatic Rectal Bacterial Pathogens Show Large Prospective Relationships With HIV Incidence in a Cohort of Young Sexual and Gender Minorities: Implications for STI Screening and HIV Prevention. Open Forum Infect Dis **2024**; 11:ofae444.

62. Mo X, Zhu C, Gan J, et al. Prevalence and correlates of Mycoplasma genitalium infection among prostatitis patients in Shanghai, China. Sex Health **2016**;

63. Miyake M, Ohnishi K, Hori S, et al. Mycoplasma genitalium Infection and Chronic Inflammation in Human Prostate Cancer: Detection Using Prostatectomy and Needle Biopsy Specimens. Cells **2019**; 8:212.

64. Justel M, Alexandre I, Martínez P, et al. Vertical transmission of bacterial eye infections, Angola, 2011-2012. Emerg Infect Dis **2015**; 21:471–473.

65. Oeser C, Pond M, Butcher P, et al. PCR for the detection of pathogens in neonatal early onset sepsis. PLoS One **2020**; 15:e0226817.

66. Towns JM, Williamson DA, Bradshaw CS. Case of Mycoplasma genitalium pelvic inflammatory disease with perihepatitis. Sex Transm Infect **2021**; 97:628.

67. Ramakrishnan G, Kronig I, Gaïa N, Lazarevic V, Schrenzel J. Mycoplasma genitalium Endocarditis in Prosthetic Aortic Valve. Emerg Infect Dis **2023**; 29:2164–2166.

68. Basu A, Biswas J, Choudhary R, Dhawan B. Chronic sexually acquired reactive arthritis, associated with extragenital Mycoplasma genitalium infection in an MSM - a case report. Indian J Med Microbiol **2025**; 56:100899.

69. Rajkumari N, Kaur H, Roy A, Gupta N, Dhaliwal LK, Sethi S. Association of Mycoplasma genitalium with infertility in North Indian women. Indian J Sex Transm Dis AIDS **2015**; 36:144–148.

70. Rekha S, Nooren M, Kalyan S, et al. Occurrence of Mycoplasma genitalium in the peritoneal fluid of fertile and infertile women with detailed analysis among infertile women. Microb Pathog **2019**; 129:183–186.

71. Lokken EM, Kabare E, Oyaro B, et al. A prospective preconception cohort study of the association between Mycoplasma genitalium and fecundability in Kenyan women trying to conceive. Hum Reprod **2023**; 38:2020–2027.

72. Ling C, Qian Y, Zhong Q. Detecting of mycoplasma genitalium infection in surgically treated infertile women. Asian J Surg **2022**; 45:2508–2509.

73. Sameni F, Zadehmodarres S, Dabiri H, Khaledi M, Nezamzadeh F. Evaluation of Ureaplasma urealyticum, Chlamydia trachomatis, Mycoplasma genitalium and Neisseria gonorrhoeae in infertile women compared to pregnant women. J Obstet Gynaecol **2022**; 42:2151–2155.

74. Mbah CE, Jasani A, Aaron KJ, et al. Association between Chlamydia trachomatis, Neisseria gonorrhea, Mycoplasma genitalium, and Trichomonas vaginalis and Secondary Infertility in Cameroon: A case-control study. PLoS One **2022**; 17:e0263186.

75. Krotik OI. CHARACTERISTICS OF THE VAGINAL MICROBIOTA, CERVICAL AND UTERINE FLORA IN WOMEN WITH THE PAST HISTORY OF SEXUALLY TRANSMITTED INFECTIONS. Wiad Lek **2021**; 74:896–901.

76. Qing L, Song Q-X, Feng J-L, Li H-Y, Liu G, Jiang H-H. Prevalence of Chlamydia trachomatis, Neisseria gonorrhoeae, Mycoplasma genitalium and Ureaplasma urealyticum infections using a novel isothermal simultaneous RNA amplification testing method in infertile males. Ann Clin Microbiol Antimicrob **2017**; 16:45.

77. Kim SJ, Paik D-J, Lee JS, et al. Effects of infections with five sexually transmitted pathogens on sperm quality. Clin Exp Reprod Med **2017**; 44:207–213.

78. Ahmadi MH, Mirsalehian A, Gilani MAS, Bahador A, Talebi M. Improvement of semen parameters after antibiotic therapy in asymptomatic infertile men infected with Mycoplasma genitalium. Infection **2018**; 46:31–38.

79. Akgul A, Kadioglu A, Koksal MO, Ozmez A, Agacfidan A. Sexually transmitted agents and their association with leucocytospermia in infertility clinic patients. Andrologia **2018**; 50:e13127.

80. Li WN, Shi L, Long XY, Li Y, Zhu WB, Liu G. Mycoplasma genitalium incidence, treatment failure, and resistance: a retrospective survey of men of infertile couples from a hospital in China. Andrology **2020**; 8:91–100.

81. Bai S, Li Y, Wan Y, et al. Sexually transmitted infections and semen quality from subfertile men with and without leukocytospermia. Reprod Biol Endocrinol **2021**; 19:92.

82. Moridi K, Ghazvini K, Hemmaty M, Hoseiniun H, Torkaman M, Fallah Mehrabadi MH. Prevalence Determination of m. Hominis and m. Genitalium in the Semen Samples in the Northeast of Iran Using Culture and Multiplex Polymerase Chain Reaction. Arch Razi Inst **2021**; 76:41–49.

83. Tjagur S, Mändar R, Poolamets O, Pomm K, Punab M. Mycoplasma genitalium Provokes Seminal Inflammation among Infertile Males. Int J Mol Sci **2021**; 22:13467.

84. Bai S, Li Y, Hu M-H, et al. Association of sexually transmitted infection with semen quality in men from couples with primary and secondary infertility. Asian J Androl **2022**; 24:317–322.

85. Al-Masri MY, Ashour IK, Swafta A, Al-Shunar S. Prevalence of Certain Urogenital Bacterial Mollicutes in Patients Suffering from Infertility. Can J Infect Dis Med Microbiol **2022**; 2022:2812788.

86. Tam Le M, Nguyen Nguyen D, Bach Nguyen H, Quynh Tram Ngo V, Quoc Huy Nguyen V. Ureaplasma urealyticum and Mycoplasma genitalium detection and sperm quality: A cross-sectional study in Vietnam. Int J Reprod Biomed **2021**; 20:185–194.

87. Ahmadi K, Moosavian M, Mardaneh J, Pouresmaeil O, Afzali M. Prevalence of Chlamydia trachomatis, Ureaplasma parvum and Mycoplasma genitalium in Infertile Couples and the Effect on Semen Parameters. Ethiop J Health Sci **2023**; 33:133–142.

88. Ashshi AM, Batwa SA, Kutbi SY, Malibary FA, Batwa M, Refaat B. Prevalence of 7 sexually transmitted organisms by multiplex real-time PCR in Fallopian tube specimens collected from Saudi women with and without ectopic pregnancy. BMC Infect Dis **2015**; 15:569.

89. Cox C, Saxena N, Watt AP, et al. The common vaginal commensal bacterium Ureaplasma parvum is associated with chorioamnionitis in extreme preterm labor. J Matern Fetal Neonatal Med **2016**; 29:3646–3651.

90. Refaat B, Ashshi AM, Batwa SA, et al. The prevalence of Chlamydia trachomatis and Mycoplasma genitalium tubal infections and their effects on the expression of IL-6 and leukaemia inhibitory factor in Fallopian tubes with and without an ectopic pregnancy. Innate Immun **2016**; 22:534–545.

91. Ramazanzadeh R, Khodabandehloo M, Farhadifar F, et al. A Case-control Study on the Relationship between Mycoplasma genitalium Infection in Women with Normal Pregnancy and Spontaneous Abortion using Polymerase Chain Reaction. Osong Public Health Res Perspect **2016**; 7:334–338.

92. Rowlands S, Danielewski JA, Tabrizi SN, Walker SP, Garland SM. Microbial invasion of the amniotic cavity in midtrimester pregnancies using molecular microbiology. Am J Obstet Gynecol **2017**; 217:71.e1-71.e5.

93. Contini C, Rotondo JC, Magagnoli F, et al. Investigation on silent bacterial infections in specimens from pregnant women affected by spontaneous miscarriage. J Cell Physiol **2018**; 234:100–107.

94. Kayem G, Doloy A, Schmitz T, et al. Antibiotics for amniotic-fluid colonization by Ureaplasma and/or Mycoplasma spp. to prevent preterm birth: A randomized trial. PLoS One **2018**; 13:e0206290.

95. Perin J, Coleman JS, Ronda J, Neibaur E, Gaydos CA, Trent M. Maternal and Fetal Outcomes in an Observational Cohort of Women With Mycoplasma genitalium Infections. Sex Transm Dis **2021**; 48:991–996.

96. Juliana NCA, Deb S, Juma MH, et al. The Vaginal Microbiota Composition and Genital Infections during and after Pregnancy among Women in Pemba Island, Tanzania. Microorganisms **2022**; 10:509.

97. Hu M, Souder JP, Subramaniam A, et al. Prevalence of Mycoplasma genitalium infection and macrolide resistance in pregnant women receiving prenatal care. Int J Gynaecol Obstet **2023**; 160:341–344.

98. Govender V, Moodley D, Naidoo M, Connoly C, Ngcapu S, Abdool Karim Q. Sexually transmitted infections in pregnancy and adverse pregnancy outcomes: A retrospective cohort study. Int J Gynaecol Obstet **2024**; 166:62–70.

99. Scoullar MJL, Melepia P, Peach E, et al. Mycoplasma genitalium in pregnancy, including specific co-infections, is associated with lower birthweight: A prospective cohort study. Med **2024**; 5:1123-1136.e3.

100. Nandagopal M, Rajan N R, Padhiar C, Abhaya M, Bansal U, Ghambir P. Revolutionizing chronic endometritis diagnosis: real-time polymerase chain reaction unveils microbial pathogens in Indian women with abnormal bleeding and reproductive challenges. AJOG Glob Rep **2024**; 4:100377.
