# Supplementary material for: Clinical Manifestations and Associated Disease States with Mycoplasma genitalium Infection: Narrative Review and State of the Literature, 2015–2025
Source: Open Forum Infect Dis. 2026 Mar 30;13(Suppl 2):S1339–51. doi: 10.1093/ofid/ofaf799 (PMC13035034; doi:10.1093/ofid/ofaf799)
Supplement: ofaf799_Supplementary_Data [file ofaf799_supplementary_data.zip › Mgen coinfection table_1016.docx]

| **Supplementary Table 3: Co-infections reported with *Mycoplasma genitalium*** | | | |
| --- | --- | --- | --- |
| **First Author, Year** | **Country** | **Population** | **Findings** |
| *Prevalence of Co-infection of other Sexually Transmitted Infections with Mycoplasma genitalium* | | | |
| Guschin, 2015[1] | Russia | Men visiting STI clinic | In the 51 with MG, CT in 2/51 (3.9%), TV in 1/51 (2.0%), HSV-2 in 1/51 (2.0%) |
| Justel, 2015[2] | Angola | Pregnant women | Among women with MG, CT in 1/19 (5.3%) |
| Campos, 2015[3] | Brazil | Women age 14-78 years | Among all participants, CT/MG in 1/302 (0.33%), NG/MG in 7/302 (2.3%) |
| Hay, 2016[4] | South Africa | Women visiting primary care clinics with sexual activity in last 6 months | Of those with MG, CT in 12/65 (19%), NG in 8/65 (12%), TV in18/65 (27%) |
| Gesink, 2016[5] | Canada | Men and women attending STI clinic | MG co-infection rate was 12.0% (6/50) with CT and/or NG |
| Getman, 2016[6] | United States | Symptomatic and asymptomatic males and females | MG presented with co-infection with TV in 6.3% and CT in 3.1% |
| Masha, 2018[7] | Kenya | Pregnant women | All 5 individuals who were MG-positive were also positive for TV, univariate analysis (COR 0.3-94.3, p = 0.002) |
| Trent, 2018[8] | United States | Pregnant and non-pregnant women aged 13–29 years at gynecologic and prenatal visits | Women with MG were 3.4 times more likely to have co-infections compared with those with other STIs (OR 3.4, 95% CI 1.17 to 10.3, P=0.02) |
| Korhonen, 2019[9] | Finland | Patients aged 18-22 | 7 cases MG and CT co-infection (4.5%) relative to 3 cases MG in CT negative group (1.9%), no significant difference |
| Latimer, 2020[10] | Australia | MSM positive for either CT or NG | MG was detected in 27 of 212 rectal CT samples (13%, 95% CI 9 to18) and in 29 of 212 rectal NG samples (14%, 95% CI 9 to 19), no significant difference |
| Perin, 2021[11] | United States | Pregnant women aged 13 to 29 years old | 24% (12/51) of those with MG were also diagnosed with CT, NG, or TV |
| Stafford, 2021[12] | United States | Pregnant women | MG infection was significantly associated with TV (p<0.001) coinfection. In addition, the rate of GBS colonization was significantly higher among women infected with MG compared with women who tested negative (58.3% vs 16.1%, p<0.01). No statistical association with CT, NG, HBV, HCV, syphilis, BV, HPV 16 or 18 |
| Richardson, 2021[13] | Australia | Men with symptomatic gonococcal urethritis | 14 of 184 (7.6%, 95% CI 3.7 to 11.5) men with gonococcal urethritis had MG detected in the urine at the time of presentation, of whom 7 were MSM |
| Pitt, 2021[14] | United Kingdom | Males and females | 9.2% (23/250) of CT tests showed MG coinfection |
| Zhang, 2021[15] | China | Symptomatic patients seeking individual STI screening or asymptomatic individuals seeking STI screening due to sexual behaviors or infected sexual contact | Of patients with MG, 10.2% (5/49) were co-infected with CT, 6.1% (3/49) with NG, 2.0% (1/49) with both CT and NG, 8.2% (4/49) with HSV-2, 4.1% (2/49) with TP and 22.4% (11/49) with BV. No statistically significant differences between percent of MG positive without and with the following coinfections- HIV (7.3% vs 0%, p=0.43), CT (7.3% vs 6.6% p=0.802), NG (7.3% vs 6.8%, 0.89), HSV2 (7.7% vs 4.3%p=0.25), TP (7.2% vs 8%, p=0.88) |
| Msemwa, 2022[16] | Tanzania | Patients with clinical diagnosis of urinary tract infection and negative urine cultures with leukocytes on urinalysis | Of 13 patients with MG, 23% (3/13 patients) with TV and MG, 8% (1/13) with CT and MG, 8% (1/13) patient with CT, TV and MG |
| Streeck, 2022[17] | Germany | MSM at risk for HIV but HIV negative aged 18-55 years | Of all participants, 2.1% (22/1043) with NG and MG, 1.7% (18/1043) with CT and MG; 8 with MG and TP; 10 participants had MG, NG and CT, and 1 participant had MG, CT and TP. |
| Lee, 2022[18] | Hong Kong | Males presenting with non-gonococcal urethritis | 2% of all 493 participants had CT and MG coinfection |
| Alamon-Reig, 2022[19] | Spain | Patients with MG | Coinfection with NG or CT in the same location was noted in 13.8% (n = 27) of all individuals |
| Melendez, 2022[20] | Uganda | Men with urethritis | Compared to participants with a negative MG test, those with MG were significantly less likely to be coinfected with other curable STIs (P = 0.008). Coinfections of MG with NG in 28.1% (n = 9/32), CT in 12.5% (n = 4/32), and TV 6.3% (n = 2/32). MG, CT, and NG coinfection was found in 12.5% (n = 4/32) |
| Maldonado-Barrueco, 2023[21] | Spain | Patients being seen at hospital with clinical STI testing | Of 56 with MG, 12.5% (7/56) with coinfection. NG in 7.1% (4/56) and CT in 5.4% (3/56) |
| Yusuf, 2023[22] | Netherlands | Patients seeking care for urethral symptoms, vaginal symptoms or their sexual contacts | MG co-infection with CT in 1.4% (0.3 to 0.6) of female patients and in 0.7% (0.5 to 0.9) of male patients. Co-infection of MG and NG was in 0.1% (0.04 to 0.2) of female patients, and in 0.2% (0.1 to 0.4) of male patients. |
| Filho, 2023[23] | Brazil | Pregnant women aged 15-45 years | CT in 15% (3/20) with MG |
| Omosa-Manyonyi, 2023 [24] | Kenya | Women presenting with lower genital tract symptoms | 14% (6 /43) with single infection, 30.2% (13/43) with dual infection, 55.8% (24/43) with at least 3 infections (other infections included vulvovaginal candidiasis, BV, TV, CT and NG) |
| Lê, 2023[25] | Canada | Males with at least one sexual encounter with a man in the prior 6 months | Among the individuals with urethral CT infection, 20% (1/5) were co-infected with MG; among those with rectal CT infection, 9.1% (2/22) were co-infected with MG; Among those with rectal NG infection, 16.7% (2/12) were co-infected with MG; no urethral NG infections observed. |
| Manhart, 2023[26] | United States | Persons tested for CT and NG at sexual health clinic with attempted equal enrollment in each of the 4 groups (symptomatic males, asymptomatic males, symptomatic females, asymptomatic females) | MG was detected in 28.2% of CT and 23.9% of NG infections. After adjusting for sampling criteria (site, birth sex, symptoms), MG was associated with CT (aPR: 1.7; 95% CI 1.13 to 2.53) but not NG |
| Menezes, 2023[27] | United States | Females aged 13-20 whose clinicians were testing for chlamydia and gonorrhea | Of all participants, 0.6% (1/152 ) with MG and CT co-infection and 1.3% (2/152) with MG and NG co-infections |
| Romo, 2024[28] | United States | Patients at military health system aged 18 to 30 years | 30.2% (13/43) with MG had a coinfection with another bacterial STI- 12 with CT alone, 1 with CT and NG |
| Manjate, 2024[29] | Mozambique | Females with any urogenital complaints | Coinfection prevalence was 0.8% with CT/MG and 0.4% with TV/MG |
| Bjartling, 2024[30] | Sweden | MSM | Total coinfection rate (syphilis and HIV excluded) in MG-positive MSM was 14.1% (9/64), including 7.8% (5/64) coinfected with NG, 4.7% (3 /64) with CT and 1.6% (1/64) with both NG and CT. Coinfection rate was 3.1% (2 /64) for TP and 14.1% (9/64) for HIV |
| Sheele, 2024[31] | United States | People tested for NG, CT, MG, TV | Among those with MG and additional testing CT 4/56 (7.1%), NG 1/56 (1.8%), TV 0/36 (0%). Testing positive for CT, NG or TV non statistically associated with MG infection (OR 2.138, 95%CI 0.92 to 6.14, p=0.08). |
| Sukhija-Cohen, 2024[32] | United States | Adult male patients presenting with symptoms of CT or NG | 45.5% (5/11) diagnosed with MG had coinfections with other STIs: 4 (36.4%) with NG only and 1 (9.1%) with both CT and TP |
| Scoullar, 2024[33] | Papau New Guinea | Pregnant women | 34.6% (18/52) of women with MG had co-infection- 14 with CT, 8 with TV, and 3 with NG |
| Lam, 2024[34] | Vietnam | Both symptomatic and asymptomatic patients visiting an STI clinic | Prevalence of coinfection- MG/CT was 6.25% (95% CI, 1.99 to 10.51), MG/NG was 1.53% (95% CI, −1.04 to 3.14) and no patient co-infected with all three bacteria. |
| Mullis, 2024[35] | United States | Patients testing positive for MG | 12.4% (57/460) with CT, 6.7% (31/460) with NG, 8.5% (32/376) with TV, 1.5% (6/397) with syphilis, 53.6% (45/84) with BV |
| Kerschberger, 2025[36] | Eswatini | Outpatients age ≥ 18 years, who were screened for STIs | In 77 with MG, 37.7% (29/77) with CT, 19.5% (15/77) with NG and 23.4% (18/77) with TV |
| Schröder, 2025[37] | Zambia | Pregnant females | CT in those MG positive 19/126 (15.1%) vs MG neg (6.2%) 54/871, p<0.001, NG in those MG positive 15.0% (19/127) vs MG neg 7.5% (65/870), p=0.005, TV 32.3% (41/127) vs MG neg 22.0% (192/873), p-value 0.01 |
| Vodstrcil, 2025[38] | Australia | Women with cervicitis | BV and MG in 13/517 (2.5%), NG and MG in 1/517 (0.2%), CT and MG in 6/517 (1.2%), BV, CT and MG in 4/517 (0.8%) |
| Nguyen, 2025[39] | Vietnam | Men who have sex with women | CT in 39.3% of those with MG, NG in 21.3% of those with MG |
| Johnson, 2025[40] | United Kingdom | Patients positive for MG at a sexual health clinic | CT 16/340 (4.7%) cases with CT alone and alongside additional STIs in 6/340 (1.8%) cases. NG 8/340 (2.4%) cases. |
| *Chlamydia Trachomatis* and *Mycoplasma genitalium* Co-infection | | | |
| Cabello Úbeda 2016[41] | Spain | MSM with symptomatic proctitis from LGV included | Among 26 with symptomatic proctitis from rectal LGV, 1/26 had MG anal coinfection |
| Chernesky, 2017[42] | Canada | Remnant samples from CT positive women and CT-negative age matched controls | The overall MG rate in CT-infected women was 13.4% compared with 5.4% in women without CT infection (P < 0.001) |
| Couldwell, 2018[43] | Australia | MSM attending an STI clinic | Anorectal C. trachomatis was independently associated with anorectal MG (OR 5.0, 95% CI 2.1 to 11.8, P<0.001) after controlling for condom use, number of male sexual partners in the last 3 months, age, anorectal N. gonorrhoeae, use of PrEP and HIV infection. HIV-infected men were not more likely than men without HIV infection to test positive for MG or to have infection with CT or NG (p=0.26). |
| Harrison, 2019[44] | United States | Women with CT | 22/302 = 7.3% had MG coinfection, presence/absence of symptoms was not associated with MG coinfection |
| Hart, 2020[45] | Singapore | All patients attending study site | 16/472 = 3.4% overall prevalence, 9/107 = 7.3% pos among CT pos/NG neg, 6/184 = 3.3% pos among CT/NG neg. Odds ratio of 3.5 for MG infection in patients with C. trachomatis infection as compared to C. trachomatis negative patients (P = 0.02, 95% CI 1.3 to 10.0). |
| Parmar, 2021[46] | Canada | Patients tested for MG | MG infection was statistically significantly (p < 0.001) higher in females with CT (12.6%; 19/151) or CT/ NG coinfection (3.9%; 6/151) as compared with those without coinfections. NG (10.5%, 4/38) infection in males was statistically significantly associated with a ( p < 0.002) MG infection |
| Che, 2022[47] | China | Females | 37% (34/92) with MG +/CT- had >15 WBCs vs 22.8% (114/500) of those MG-, CT-, p-value <0.05; MG alone did not increase WBCs in as many participants at CT (52.6%, 141/268). In patients MG+ and CT+ 74.1% (20/27) with > 15 WBCs, p<0.05 when compared to all other groups [co-infection of MG and CT can aggravate the increase of WBCs, compared to MG or CT infection alone]; Compared with patients with MG or CT infection alone, patients with MG and CT co-infection had significantly less Lactobacillus in leucorrhea secretion. MG or CT was significantly related to the increase of clue cells in vaginal secretion (P < 0.001) |
| Brin, 2022[48] | France | All patients tested at center | Chlamydia coinfection more likely in women with MG than men (26%, 20/78 vs 13%, 41/319, p-value=0.006). NG coinfection more likely in men (27/319, 8.5%) vs women (0/78, 0%) with MG (p=0.008). |
| Wang, 2022[49] | United States | Adolescent and young adult women | MG associated with increased adjusted prevalence ratio for CT 3.02 (1.69, 5.39) |
| Manhart, 2023[26] | United States | Persons tested for CT and NG at sexual health clinic- tried to identify 100 specimens per cycle from each of 4 groups (symptomatic males, asymptomatic males, symptomatic females, asymptomatic females) at each site | MG was detected in 28.2% of CT and 23.9% of GC infections. After adjusting for sampling criteria (site, birth sex, symptoms), MG was associated with CT (aPR: 1.7; 95% CIT 1.13 to 2.53) but not GC |
| Dirks, 2023[50] | Netherlands | Patients from general population aged 16-30 years who participated in a trial, or patients visiting an STI clinic | No correlation between the urogenital MG and CT load in MG/CT coinfected individuals (beta, 0.2; 95% CI, −0.05 to 0.24; P = 0.2 |
| Bacterial Vaginosis and *M. genitalium* | | | |
| Napierala Mavedzenge, 2015[51] | Zimbabwe | Women with new HIV-1 diagnosis <6 months | BV associated with increased prevalence of MG (aOR 2.24, 95% CI 1.03 to 4.85) in multivariable model |
| Lokken, 2017[52] | Kenya | Women age >16 and reporting exchanging sex for cash or in-kind payments | At baseline 40.4% (113/280) women had BV and 16.1% had MG. BV was detected at least once in 73.9% (207/280) of women at 38.4% (940/2,448) of visits with BV data. Only 8.4% (79/940) of visits with BV were accompanied by symptoms (vaginal discharge and/or itching), and metronidazole was prescribed at only 2.6% (24/940) of visits with BV. Among women with BV, 55.1% (114/207) had at least 1 recurrence of BV, with a maximum of 4 recurrences. |
| Seña, 2018[53] | United States | Women with asymptomatic BV ages 15-25 | 233/1139 = 20.5% (18.2-22.9) prevalence MG |
| Nye, 2020[54] | United States | Women with vaginitis symptoms | MG (p= .02) infections were significantly more common in women with BV than those without this condition (OR 1.97, 95% CI: 1.14 to 3.39) |
| Shipitsyna, 2020[55] | Russia | Outpatients attending routine gynecological care | Significantly higher odds of testing positive for MG for women with BV-associated microbiota than women with normal microbiota, (OR 2.88, 95% CI 1.19 to 7.16) |
| Moore, 2021[56] | United States | African American women in Detroit area | The percent detection of BV-associated bacterial species did not differ by MG status for most species; however, both the detection and quantity of Leptotrichia/Sneathia (detection PR: 2.9 (95% CI 1.1 to 7.7); quantity PR: 1.2 (95% CI 1.0 to 1.3)) and Megasphaera phylotype 1 (detection PR: 2.2, (95% CI 1.2 to 4.2); quantity PR: 1.1 (95% CI 1.0 to 1.2)) were associated with MG |
| Wiesenfeld, 2021[57] | United States | Women diagnosed with acute PID | At baseline 41 (18%) of women had MG and BV was present in 127 women (55%). Even though metronidazole has no activity against MG, cervical infection following PID treatment was less frequent in women receiving metronidazole than in women in the placebo-containing group (4% vs 14%, P < .05). BV (20% vs 54%, P < .001) and TV (5% vs 12%, p= .10) were also less prevalent in women who were randomized to metronidazole. |
| Zhang, 2023[58] | China | Females with purulent cervical secretions or abnormal vaginal microecology were included as the research group, and those with normal vaginal microecology and cervical secretions were included as the control group | MG was close to statistical association with presence of BV, P = 0.057 or mixed vaginitis, P = 0.081; Among those with BV 12/244 with MG (4.9%), 232/244 (95.1%) are MG neg |
| Schwebke, 2024[59] | United States | Remnant specimens from a multi-center cross-sectional observational study to validate assays | MG infection was significantly associated with positive modified Amsel criteria - OR 1.9821 (95% CI 1.2936 to 2.8739); MG solo infection associated with diagnosis of BV +/ VVC - (OR 3.0751; 95% CI 1.5797 to 5.9858, P = 0.0113), mixed infection with MG and another STI also associated with diagnosis of BV+/ VVC- ( OR 3.4886; 95% CI 1.8901 to 6.439, P = 0.0042 |
| Mullis, 2024[35] | United States | Patients with MG | Females with BV had a higher percentage of microbiologic cure of MG relative to both those testing negative for BV (77% vs 40%, p-value <0.01) and all other females (those testing negative and those not tested for BV) (77% vs 6 43%, p-value <0.01) |
| Human immunodeficiency virus (HIV) | | | |
| Napierala Mavedzenge, 2015[51] | Zimbabwe | Women with new HIV-1 diagnosis <6 months | Genital HIV-1 RNA more likely in endocervical samples with MG compared to no MG 71.4% vs 58.3%, aOR 2.81 (95% CI 1.01 to 7.83) |
| Hay, 2015[60] | South Africa | Women visiting primary care clinics reporting sexual activity in the last 6 months | HIV coinfection aOR 2.1 (95% CI 1.1 to 4.1) in multivariable model associated with MG |
| Chen, 2015[61] | China | Men living with HIV-1 older than age 18 in Jiangsu province | MG prevalence was 24.0% vs 15.3% for those with CD4 cell count <350 or >350 respectively, p<0.001 |
| Lokken, 2017[52] | Kenya | Women age >16 and reporting exchanging sex for cash or in-kind payments | Women with MG were more likely to be HIV-positive (68.9% vs. 51.5%; P = 0.04) and younger (median age, 33.5 years vs. 35.5 years; P = 0.003) than those without MG |
| Adebamowo, 2017[62] | Nigeria | Women enrolled in a study of HPV infection and cervical cancer | At baseline, HIV positive women were more likely to have persistent MG (p < 0.001). In longitudinal analysis, no significant association between being positive for HIV and prevalent or persistent MG was noted. |
| Zhao, 2019[63] | China | MSM who sought voluntary HIV counseling and testing | MG infection associated with HIV infection (aOR = 3.2, 95%CI, 1.3 to 7.7, P = 0.012) |
| Roxby, 2019[64] | Kenya | Pregnant women, PLWH, <28 weeks gestation | Mean plasma HIV RNA levels at 32 weeks gestation were significantly higher among MG-infected mothers, (5.0 log10 copies/ml for mothers with MG v. 4.6 log10 copies/ml for mothers without MG, p=0.02). Mothers with MG were more likely to be diagnosed with TV while pregnant (28% vs. 16%, p=0.07). |
| Mahlangu, 2019[65] | South Africa | Patients with male urethral syndrome of vaginal discharge syndrome enrolled | Prevalence of HIV coinfection in those with MG, was significantly higher than in those without MG infection (49.6% vs. 40.5%, P = 0.006) |
| Smullin, 2020[66] | South Africa | Pregnant women receiving antenatal care | 24% (95% CI 16% to 34%, n=22) of women living with HIV and 12% (95% CI 6.8% to 20%, n=13) of women living without HIV were infected with MG |
| Kharsany, 2020[67] | South Africa | Males, females ages 15-49 | Being HIV positive was associated with an increased probability of having MG (aPR=1.49, 95% CI 1.02 to 2.19) |
| Jary, 2021[68] | Mali | Women seen at an outpatient clinic | MG prevalence of 11% (5/44) in PLWH, 8% (8/96) in persons without HIV, p=0.55 |
| Herms, 2022[69] | France | All patients seen and tested for MG in study period | HIV+ status was significantly associated with MG+ (OR = 7.25, 95%CI = 1.31 to 41.73, P = 0.02 after adjustment for age, sexual orientation and number of sexual partners). |
| Baiers, 2024[70] | United States | Young adult sexual and gender minorities assigned male at birth | In bivariate GEE analysis, MG (OR, 3.04; 95% CI, 1.17 to 7.92) was found to be a statistically significant risk factor for HIV seroconversion compared with participants who tested nonreactive. Multivariate analysis, after controlling for demographic characteristics and sexual risk behavior, rectal MG was not found to be a statistically significant predictor of HIV seroconversion (adjusted odds ratio [AOR], 1.82; 95% CI, 0.61 to 5.44). However, the presence of rectal NG (aOR, 5.11; 95% CI, 1.20 to 21.77) and number of condomless anal sex partners (aOR, 1.11; 95% CI, 1.00 to 1.23) were found to be statistically significant predictors. |
| Lindman, 2024[71] | Guinea-Bissau | Female sex workers | MG was significantly more common in HIV-1-infected participants compared with HIV-1-negative participants adjusted for age, 30.2% (26/86) vs 18.8% (43/229) (OR 2.05, 95% CI 1.14 to 3.70, p=0.016) |
| Schröder, 2025[37] | Zambia | Pregnant females | HIV seropositivity 14.3% (18/126) vs (7.5%) 65/865, p-value 0.01 |
| Human papillomavirus (HPV) | | | |
| Vielot, 2015[72] | Kenya | Female sex workers attending clinic | Cervical HPV 16/173 = 9.2% MG coinfection, MG coinfection not associated with longer duration of hrHPV positivity (p=0.23) |
| Wohlmeister, 2016[73] | Brazil | Women undergoing routine gynecologic examination | High-risk cervical HPV not associated with MG infection (only 3 cases of MG) |
| de Abreu, 2016[74] | Brazil | Women in a public health system with referral for colposcopy | MG prevalence was 3.5% out of 838 women in the study. Co-infection with MG did not significantly increase the risk of HPV or HR-HPV |
| Ferré, 2019[75] | Togo | MSM >18 | In multivariable analysis hrHPV anal infection among MSM was significantly and independently associated with MG anal infection aOR 9.61 (3.08-29.89) |
| Mortaki, 2020[76] | Greece | Women in 3 groups: anogenital warts, cervical HPV, routine check-up (controls) | MG found in 8/196 AGW (4.1%), 2/315 cervical HPV (0.6%), 1/178 routine check-up (0.6%), significantly higher in AGW group =-0.005 |
| Jary, 2021[68] | Mali | women seen at clinic | In adjusted model MG associated with hrHPV OR 6.55 (95% CI 1.47 to 66.6), p=0.081 |
| Xie, 2021[77] | China | Women undergoing gyn exam | 10/668 = 1.5% prevalence (all in HPV positive cases), The proportion of patients with Mg infection was 4.13% (5/121) and 0.91% (5/547) in the CIN group and the control group, respectively, suggesting that the risk of CIN was higher in patients with Mg infection (OR=4.207; 95% CI, 1.160 to 15.260; P=0.029). |
| Tovo, 2021[78] | Burkina Faso | Female sex workers | MG not statistically associated with HPV (12%, 12/100 HPV+ with MG vs 11.0%, 9/82 HPV- with MG, p=0.83 |
| Che, 2022[47] | China | Females | hrHPV- no significant relationship between MG or CT infection and high-risk HPV infection (P = 0.123) |
| Jiang, 2023[79] | China | Women getting a colposcopy | MG in 16 individuals (3.64%) with HPV infection; Not associated with severity of cervical lesions in 3 adjusted models |
| Valasoulis, 2023[80] | Greece | Women attending GYN department for general GYN exam or routine cervical screening or colposcopy | MG not statistically significantly associated with cervical dysplasia: 0.38% (3/800) negative for intraepithelial lesion with MG, 1.6% (5/78) with ASCUS with MG, 1.6% (5/313) with LSIL with MG, 0/6 with HSIL with MG, p-value 0.1028 |
| Rodrigues, 2023[81] | Brazil | Women both HIV-infected and HIV-uninfected women | Fair concordance of anal and genital HPV with MG- kappa 0.23 (95% CI, −0.03 to 0.66, p-value 0.003) among all participants; in HIV uninfected participants kappa 0.26 (−0.04–0.66), p-value 0.005 |
| Pella-Saavedra, 2023[82] | Peru | Asymptomatic women | Among those with high risk HPV 1.25% (2/160) with MG, in group with low risk HPV (n=42)- no MG coinfections |
| Arévalos, 2024[83] | Paraguay | HPV positive women | hrHPV- overall 1.3% MG positive (3/231), 1/185 (0.5%) with hr-HPV without CIN3 lesion and 2/46 (4.4%) of those with hrHPV with CIN3 lesion, p-value 0.12- no association between presence of CIN3 lesion among those positive for hrHPV and MG detection |
| Klein, 2024[84] | Ethiopia | Asymptomatic pregnant women | MG associated with HPV-positive- 6/257, 2.3% (0.5-4.2%) of HPV-positive also with MG, p= 0.02; MG not associated with hrHPV 2/172 (1.2%, 95% CI 0.2 to 4.6%) of those with hrHPV positive for MG, p-value=0.69 |
| Rizzo, 2024[85] | Italy | Sexually active and reported unprotected intercourse | Rectal HPV: Among those positive for MG and HPV. 34% (18/53) of HR-HPV, 35% (13/37) of probable/possible HR-HPV and 31% (13/42) of LR-HPV were positive for MG patients |
| Zonta, 2024[86] | Brazil | Incarcerated women | HPV positive with MG 2.2% (4/186) vs HPV neg with MG 4.5% (5/112), p=0.26 |
| Other infections associated with MG | | | |
| Cameron, 2018[87] | Kenya | Female sex workers attending clinic | EBV DNA found in cervix of 62/330 women, including 29% of 42 women with MG and 17% of 288 without MG, age-adjusted prevalence ratio: 1.73 (1.03, 2.92) |
| Sturt, 2021[88] | Zambia | Women aged 18-31 years, not pregnant and sexually active | 7/158 (4.4%) negative for Genital Schisto with MG vs 0/30 (0%) with FGS positive for MG, p-value 0.60 |
| Rizzo, 2023[89] | Italy | Patients with Mpox | 15.6% (5/32) patients with Mpox with MG coinfection (3 uretheral swabs, 1 urine swab, 1 anal swab) |
| Maldonado-Barrueco, 2023[90] | Spain | Patients with Mpox | MG in 8.6% (3/36) patients with Mpox, (2 in rectum, 1 in urine) |

Abbreviations: STI: sexually transmitted infection; MG: *Mycoplasma genitalium*; CT: *Chlamydia trachomatis*; TV: *Trichomonas vaginalis*; HSV-2: Herpes simplex virus-2; NG: *Neisseria gonorrhea*; HBV: Hepatitis B virus; HCV: Hepatitis C virus; BV: Bacterial vaginosis; HPV: Human papillomavirus; LR-HPV: low risk human papillomavirus; hrHPV: high risk human papillomavirus; MSM: men who have sex with men; OR: odds ratio; aOR: adjusted odds ratio; PR: prevalence ratio; aPR: adjusted prevalence ratio; CI: confidence interval; HIV: human immunodeficiency virus; TP: *Treponema pallidum*; Mpox: monkeypox virus; vs: versus

References:

1. Guschin A, Ryzhikh P, Rumyantseva T, Gomberg M, Unemo M. Treatment efficacy, treatment failures and selection of macrolide resistance in patients with high load of Mycoplasma genitalium during treatment of male urethritis with josamycin. BMC Infect Dis **2015**; 15:40.

2. Justel M, Alexandre I, Martínez P, et al. Vertical transmission of bacterial eye infections, Angola, 2011-2012. Emerg Infect Dis **2015**; 21:471–473.

3. Campos GB, Lobão TN, Selis NN, et al. Prevalence of Mycoplasma genitalium and Mycoplasma hominis in urogenital tract of Brazilian women. BMC Infect Dis **2015**; 15:60.

4. Hay PE, Kerry SR, Normansell R, et al. Which sexually active young female students are most at risk of pelvic inflammatory disease? A prospective study. Sex Transm Infect **2016**; 92:63–66.

5. Gesink D, Racey CS, Seah C, et al. Mycoplasma genitalium in Toronto, Ont: Estimates of prevalence and macrolide resistance. Can Fam Physician Med Fam Can **2016**; 62:e96-101.

6. Getman D, Jiang A, O’Donnell M, Cohen S. Mycoplasma genitalium Prevalence, Coinfection, and Macrolide Antibiotic Resistance Frequency in a Multicenter Clinical Study Cohort in the United States. J Clin Microbiol **2016**; 54:2278–2283.

7. Masha SC, Cools P, Descheemaeker P, Reynders M, Sanders EJ, Vaneechoutte M. Urogenital pathogens, associated with Trichomonas vaginalis, among pregnant women in Kilifi, Kenya: a nested case-control study. BMC Infect Dis **2018**; 18:549.

8. Trent M, Coleman JS, Hardick J, et al. Clinical and sexual risk correlates of Mycoplasma genitalium in urban pregnant and non-pregnant young women: cross-sectional outcomes using the baseline data from the Women’s BioHealth Study. Sex Transm Infect **2018**; 94:411–413.

9. Korhonen S, Hokynar K, Eriksson T, et al. The Prevalence of HSV, HHV-6, HPV and Mycoplasma genitalium in Chlamydia trachomatis positive and Chlamydia trachomatis Negative Urogenital Samples among Young Women in Finland. Pathog Basel Switz **2019**; 8:276.

10. Latimer RL, Vodstrcil L, De Petra V, et al. Extragenital Mycoplasma genitalium infections among men who have sex with men. Sex Transm Infect **2020**; 96:10–18.

11. Perin J, Coleman JS, Ronda J, Neibaur E, Gaydos CA, Trent M. Maternal and Fetal Outcomes in an Observational Cohort of Women With Mycoplasma genitalium Infections. Sex Transm Dis **2021**; 48:991–996.

12. Stafford IA, Hummel K, Dunn JJ, et al. Retrospective analysis of infection and antimicrobial resistance patterns of Mycoplasma genitalium among pregnant women in the southwestern USA. BMJ Open **2021**; 11:e050475.

13. Richardson D, Lewis DA, Jeoffreys NJ, Couldwell DL. Mycoplasma genitalium coinfection in men with symptomatic gonococcal urethritis. Sex Transm Infect **2021**; 97:363–367.

14. Pitt R, Fifer H, Woodford N, Hopkins S, Cole MJ. Prevalence of Chlamydia trachomatis and Mycoplasma genitalium coinfections and M. genitalium antimicrobial resistance in rectal specimens. Sex Transm Infect **2021**; 97:469–470.

15. Zhang X-H, Zhao P-Z, Ke W-J, et al. Prevalence and correlates of Mycoplasma genitalium infection among patients attending a sexually transmitted infection clinic in Guangdong, China: a cross-sectional study. BMC Infect Dis **2021**; 21:649.

16. Msemwa B, Mushi MF, Kidenya B, et al. Urogenital pathogens in urine samples of clinically diagnosed urinary tract infected patients in Tanzania: A laboratory based cross-sectional study. IJID Reg **2023**; 7:170–175.

17. Streeck H, Jansen K, Crowell TA, et al. HIV pre-exposure prophylaxis was associated with no impact on sexually transmitted infection prevalence in a high-prevalence population of predominantly men who have sex with men, Germany, 2018 to 2019. Euro Surveill Bull Eur Sur Mal Transm Eur Commun Dis Bull **2022**; 27:2100591.

18. Lee SS, Cheng KF, Wong NS, et al. Emergence of antibiotic-resistant Mycoplasma genitalium as the cause of non-gonococcal urethritis in male patients at a sexually transmitted infection clinic. Int J Antimicrob Agents **2022**; 59:106510.

19. Alamon-Reig F, Riera-Monroig J, González-Cordón A, Bosch J, Alsina M, Fuertes I. Mycoplasma genitalium: A descriptive study of 196 cases. Indian J Dermatol Venereol Leprol **2022**; 88:873.

20. Melendez JH, Hardick J, Onzia A, et al. Retrospective Analysis of Ugandan Men with Urethritis Reveals Mycoplasma genitalium and Associated Macrolide Resistance. Microbiol Spectr **2022**; 10:e0230421.

21. Maldonado-Barrueco A, Rodríguez-Ayala M, Grandioso-Vas D, et al. Epidemiology and prevalence of mutations associated with resistance to macrolides and fluoroquinolones in Mycoplasma genitalium in a tertiary hospital from Madrid, Spain. Rev Espanola Quimioter Publicacion Of Soc Espanola Quimioter **2023**; 36:310–313.

22. Yusuf E, Mertens K, van Lisdonk N, Houwen C, Thai KTD. Epidemiology of Mycoplasma genitalium and Trichomonas vaginalis in the primary health care setting in the Netherlands. Epidemiol Infect **2023**; 151:e79.

23. Filho AC, Marcos CRSA, Colnago JM, Miranda AEB, Duarte JN, Peruchi LS. Sexually transmitted infections with Chlamydia trachomatis, Neisseria gonorrhoeae, Mycoplasma genitalium, and Trichomonas vaginalis in pregnant women as detected by molecular testing. Indian J Sex Transm Dis AIDS **2023**; 44:139–142.

24. Omosa-Manyonyi GS, de Kam M, Tostmann A, et al. Evaluation and optimization of the syndromic management of female genital tract infections in Nairobi, Kenya. BMC Infect Dis **2023**; 23:547.

25. Lê A-S, Labbé A-C, Fourmigue A, et al. Mycoplasma genitalium infection among gay, bisexual and other men who have sex with men in Montréal, Canada. Can Commun Dis Rep Releve Mal Transm Au Can **2023**; 49:477–486.

26. Manhart LE, Leipertz G, Soge OO, et al. Mycoplasma genitalium in the US (MyGeniUS): Surveillance Data From Sexual Health Clinics in 4 US Regions. Clin Infect Dis Off Publ Infect Dis Soc Am **2023**; 77:1449–1459.

27. Menezes ME, Silver EJ, Goldstein DY, Collins-Ogle MD, Fox AS, Coupey SM. Prevalence and Factors Associated With Mycoplasma genitalium Infection in At-Risk Female Adolescents in Bronx County, New York. Sex Transm Dis **2023**; 50:635–641.

28. Romo ML, Moreland SC, Yates AM, et al. Prevalence of Urogenital Mycoplasma genitalium Infection at 2 US Army Medical Facilities. Sex Transm Dis **2024**; 51:367–373.

29. Manjate A, Sergon G, Kenga D, et al. Prevalence of sexually transmitted infections (STIs), associations with sociodemographic and behavioural factors, and assessment of the syndromic management of vaginal discharge in women with urogenital complaints in Mozambique. Front Reprod Health **2024**; 6:1323926.

30. Bjartling C, Kertes R, Kristiansen S, Johnsson A, Forslund O. Prevalence of Mycoplasma genitalium and macrolide resistance in rectal and urine samples among men who have sex with men in Sweden. Sex Transm Infect **2024**; 100:430–434.

31. Sheele JM, Bragg K. Features Associated With Mycoplasma genitalium Infection. Cureus **2024**; 16:e72728.

32. Sukhija-Cohen AC, Patani H, Robinson AC, Santos MR, Granados Y. Mycoplasma genitalium Incidence, Coinfection, and Antibiotic Resistance: A Prospective Study at a Walk-In Clinic in Los Angeles County, CA. Open Forum Infect Dis **2024**; 11:ofae419.

33. Scoullar MJL, Melepia P, Peach E, et al. Mycoplasma genitalium in pregnancy, including specific co-infections, is associated with lower birthweight: A prospective cohort study. Med N Y N **2024**; 5:1123-1136.e3.

34. Lam PPH, Nguyen NH, Nguyen TTT, Trinh NB, Luong BA. Mycoplasma genitalium prevalence, co-infection and macrolide resistance-associated mutations in Southern Vietnam. Infez Med **2024**; 32:222–230.

35. Mullis CE, Marlow KA, Maity A, et al. Clinical Presentations and Treatment Outcomes of Mycoplasma genitalium Infections at a Large New York City Health Care System. Sex Transm Dis **2024**; 51:199–205.

36. Kerschberger B, Lekelem S, Daka M, et al. Mycoplasma genitalium infection in Eswatini amid syndromic case management: prevalence, coinfections, diagnostic challenges and treatment gaps. BMC Infect Dis **2025**; 25:547.

37. Schröder D, Sorano S, Shipitsyna E, et al. Prevalence and epidemiology of Mycoplasma genitalium and the absence of macrolide resistance in M. genitalium among pregnant women attending antenatal care in Zambia. Front Public Health **2025**; 13:1576376.

38. Vodstrcil LA, Plummer EL, Nguyen TV, et al. Trends in infections detected in women with cervicitis over a decade. Front Reprod Health **2025**; 7:1539186.

39. Nguyen HB, Nguyen HDK, Pham MQ, Nguyen CT, Adamson PC. Clinical characteristics and symptoms associated with Mycoplasma genitalium infections among heterosexual men in Hanoi, Vietnam. Sex Transm Infect **2025**; 101:361–366.

40. Johnson K, Buluwela E, McDonald G, et al. Mycoplasma genitalium treatment outcomes among a cohort failing macrolide resistance-guided treatment across three London sexual health clinics. Sex Transm Infect **2025**; 101:5–9.

41. Cabello Úbeda A, Fernández Roblas R, García Delgado R, et al. Anorectal Lymphogranuloma Venereum in Madrid: A Persistent Emerging Problem in Men Who Have Sex With Men. Sex Transm Dis **2016**; 43:414–419.

42. Chernesky MA, Jang D, Martin I, et al. Mycoplasma genitalium Antibiotic Resistance-Mediating Mutations in Canadian Women With or Without Chlamydia Trachomatis Infection. Sex Transm Dis **2017**; 44:433–435.

43. Couldwell DL, Jalocon D, Power M, Jeoffreys NJ, Chen SC-A, Lewis DA. Mycoplasma genitalium: high prevalence of resistance to macrolides and frequent anorectal infection in men who have sex with men in western Sydney. Sex Transm Infect **2018**; 94:406–410.

44. Harrison SA, Olson KM, Ratliff AE, et al. Mycoplasma genitalium Coinfection in Women With Chlamydia trachomatis Infection. Sex Transm Dis **2019**; 46:e101–e104.

45. Hart T, Tang WY, Mansoor SAB, Chio MTW, Barkham T. Mycoplasma genitalium in Singapore is associated with Chlamydia trachomatis infection and displays high macrolide and Fluoroquinolone resistance rates. BMC Infect Dis **2020**; 20:314.

46. Parmar NR, Mushanski L, Wanlin T, et al. High Prevalence of Macrolide and Fluoroquinolone Resistance-Mediating Mutations in Mycoplasma genitalium-Positive Urine Specimens From Saskatchewan. Sex Transm Dis **2021**; 48:680–684.

47. Che G, Liu F, Yang Q, et al. Mycoplasma genitalium and Chlamydia trachomatis infection among women in Southwest China: a retrospective study. Epidemiol Infect **2022**; 150:e129.

48. Brin C, Palich R, Godefroy N, et al. Clinical, epidemiological and therapeutic characteristics of Mycoplasma genitalium infection in a French STI center. Infect Dis Now **2022**; 52:13–17.

49. Wang R, Trent ME, Bream JH, et al. Mycoplasma genitalium Infection Is Not Associated With Genital Tract Inflammation Among Adolescent and Young Adult Women in Baltimore, Maryland. Sex Transm Dis **2022**; 49:139–144.

50. Dirks JAMC, van Loo IHM, Dukers-Muijrers NHTM, Wolffs PFG, Hoebe CJPA. Chlamydia trachomatis Coinfection Does Not Influence Mycoplasma genitalium Bacterial Load in Urogenital Samples. Sex Transm Dis **2023**; 50:157–160.

51. Napierala Mavedzenge S, Müller EE, Lewis DA, Chipato T, Morrison CS, Weiss HA. Mycoplasma genitalium is associated with increased genital HIV type 1 RNA in Zimbabwean women. J Infect Dis **2015**; 211:1388–1398.

52. Lokken EM, Balkus JE, Kiarie J, et al. Association of Recent Bacterial Vaginosis With Acquisition of Mycoplasma genitalium. Am J Epidemiol **2017**; 186:194–201.

53. Seña AC, Lee JY, Schwebke J, et al. A Silent Epidemic: The Prevalence, Incidence and Persistence of Mycoplasma genitalium Among Young, Asymptomatic High-Risk Women in the United States. Clin Infect Dis Off Publ Infect Dis Soc Am **2018**; 67:73–79.

54. Nye MB, Harris AB, Pherson AJ, Cartwright CP. Prevalence of Mycoplasma genitalium infection in women with bacterial vaginosis. BMC Womens Health **2020**; 20:62.

55. Shipitsyna E, Khusnutdinova T, Budilovskaya O, et al. Bacterial vaginosis-associated vaginal microbiota is an age-independent risk factor for Chlamydia trachomatis, Mycoplasma genitalium and Trichomonas vaginalis infections in low-risk women, St. Petersburg, Russia. Eur J Clin Microbiol Infect Dis Off Publ Eur Soc Clin Microbiol **2020**; 39:1221–1230.

56. Moore KR, Tomar M, Taylor BD, Gygax SE, Hilbert DW, Baird DD. Mycoplasma genitalium and Bacterial Vaginosis-Associated Bacteria in a Non-Clinic-Based Sample of African American Women. Sex Transm Dis **2021**; 48:118–122.

57. Wiesenfeld HC, Meyn LA, Darville T, Macio IS, Hillier SL. A Randomized Controlled Trial of Ceftriaxone and Doxycycline, With or Without Metronidazole, for the Treatment of Acute Pelvic Inflammatory Disease. Clin Infect Dis Off Publ Infect Dis Soc Am **2021**; 72:1181–1189.

58. Zhang Z, Zong X, Bai H, Fan L, Li T, Liu Z. Prevalence of Mycoplasma genitalium and Chlamydia trachomatis in Chinese female with lower reproductive tract infection: a multicenter epidemiological survey. BMC Infect Dis **2023**; 23:2.

59. Schwebke JR, Nyirjesy P, Dsouza M, Getman D. Vaginitis and risk of sexually transmitted infections: results of a multi-center U.S. clinical study using STI nucleic acid amplification testing. J Clin Microbiol **2024**; 62:e0081624.

60. Hay B, Dubbink JH, Ouburg S, et al. Prevalence and macrolide resistance of Mycoplasma genitalium in South African women. Sex Transm Dis **2015**; 42:140–142.

61. Chen L-S, Wu J-R, Wang B, et al. Epidemiology of Mycoplasma acquisition in male HIV-1 infected patients: a multistage cross-sectional survey in Jiangsu, China. Epidemiol Infect **2015**; 143:3327–3334.

62. Adebamowo SN, Ma B, Zella D, et al. Mycoplasma hominis and Mycoplasma genitalium in the Vaginal Microbiota and Persistent High-Risk Human Papillomavirus Infection. Front Public Health **2017**; 5:140.

63. Zhao N, Li KT, Gao Y-Y, Xu J-J, Huang D-S. Mycoplasma Genitalium and Mycoplasma Hominis are prevalent and correlated with HIV risk in MSM: a cross-sectional study in Shenyang, China. BMC Infect Dis **2019**; 19:494.

64. Roxby AC, Yuhas K, Farquhar C, et al. Mycoplasma genitalium infection among HIV-infected pregnant African women and implications for mother-to-child transmission of HIV. AIDS Lond Engl **2019**; 33:2211–2217.

65. Mahlangu MP, Müller EE, Venter JME, Maseko DV, Kularatne RS. The Prevalence of Mycoplasma genitalium and Association With Human Immunodeficiency Virus Infection in Symptomatic Patients, Johannesburg, South Africa, 2007-2014. Sex Transm Dis **2019**; 46:395–399.

66. Smullin CP, Green H, Peters R, et al. Prevalence and incidence of Mycoplasma genitalium in a cohort of HIV-infected and HIV-uninfected pregnant women in Cape Town, South Africa. Sex Transm Infect **2020**; 96:501–508.

67. Kharsany ABM, McKinnon LR, Lewis L, et al. Population prevalence of sexually transmitted infections in a high HIV burden district in KwaZulu-Natal, South Africa: Implications for HIV epidemic control. Int J Infect Dis IJID Off Publ Int Soc Infect Dis **2020**; 98:130–137.

68. Jary A, Teguete I, Sidibé Y, et al. Prevalence of cervical HPV infection, sexually transmitted infections and associated antimicrobial resistance in women attending cervical cancer screening in Mali. Int J Infect Dis IJID Off Publ Int Soc Infect Dis **2021**; 108:610–616.

69. Herms F, Poizeau F, Anyfantakis V, et al. Mycoplasma genitalium screening in a specialized French unit: A retrospective study. Ann Dermatol Venereol **2022**; 149:165–168.

70. Baiers RA, Ryan DT, Clifford A, et al. Asymptomatic Rectal Bacterial Pathogens Show Large Prospective Relationships With HIV Incidence in a Cohort of Young Sexual and Gender Minorities: Implications for STI Screening and HIV Prevention. Open Forum Infect Dis **2024**; 11:ofae444.

71. Lindman J, Djalo MA, Biai A, et al. Prevalence of sexually transmitted infections and associated risk factors among female sex workers in Guinea-Bissau. Sex Transm Infect **2024**; 100:411–417.

72. Vielot N, Hudgens MG, Mugo N, Chitwa M, Kimani J, Smith J. The Role of Chlamydia trachomatis in High-Risk Human Papillomavirus Persistence Among Female Sex Workers in Nairobi, Kenya. Sex Transm Dis **2015**; 42:305–311.

73. Wohlmeister D, Vianna DRB, Helfer VE, et al. Association of human papillomavirus and Chlamydia trachomatis with intraepithelial alterations in cervix samples. Mem Inst Oswaldo Cruz **2016**; 111:106–113.

74. de Abreu AL, Malaguti N, Souza RP, et al. Association of human papillomavirus, Neisseria gonorrhoeae and Chlamydia trachomatis co-infections on the risk of high-grade squamous intraepithelial cervical lesion. Am J Cancer Res **2016**; 6:1371–1383.

75. Ferré VM, Gbeasor-Komlanvi FA, Collin G, et al. Prevalence of Human Papillomavirus, Human Immunodeficiency Virus, and Other Sexually Transmitted Infections Among Men Who Have Sex With Men in Togo: A National Cross-sectional Survey. Clin Infect Dis Off Publ Infect Dis Soc Am **2019**; 69:1019–1026.

76. Mortaki D, Tsitsopoulos E, Louizou E, et al. Prevalence of Cervico-vaginal High-risk HPV Types and Other Sexually Transmitted Pathogens in Anogenital Warts Patients. Anticancer Res **2020**; 40:2219–2223.

77. Xie L, Li Q, Dong X, et al. Investigation of the association between ten pathogens causing sexually transmitted diseases and high-risk human papilloma virus infection in Shanghai. Mol Clin Oncol **2021**; 15:132.

78. Tovo SF, Zohoncon TM, Dabiré AM, et al. Molecular Epidemiology of Human Papillomaviruses, Neisseria gonorrhoeae, Chlamydia trachomatis and Mycoplasma genitalium among Female Sex Workers in Burkina Faso: Prevalence, Coinfections and Drug Resistance Genes. Trop Med Infect Dis **2021**; 6:90.

79. Jiang M, Ding H, He L, et al. Association between co-infection with Chlamydia trachomatis or Mycoplasma genitalium and cervical lesions in HPV-positive population in Hunan, China: a cross-sectional study. Infect Agent Cancer **2023**; 18:76.

80. Valasoulis G, Pouliakis A, Michail G, et al. Cervical HPV Infections, Sexually Transmitted Bacterial Pathogens and Cytology Findings-A Molecular Epidemiology Study. Pathog Basel Switz **2023**; 12:1347.

81. Rodrigues LLS, Pilotto JH, Martinelli KG, et al. Diversity of Anal HPV and Non-HPV Sexually Transmitted Infections and Concordance with Genital Infections in HIV-Infected and HIV-Uninfected Women in the Tapajós Region, Amazon, Brazil. Viruses **2023**; 15:1328.

82. Pella-Saavedra P, Ramos-Vallejos F, Del Valle-Mendoza J, et al. Prevalence of coinfections in a cross-sectional cohort of women screened for multiple pathogens in Peru. Heliyon **2023**; 9:e14257.

83. Arévalos A, Valenzuela A, Mongelós P, et al. Genital infections in high-risk human papillomavirus positive Paraguayan women aged 30-64 with and without cervical lesions. PloS One **2024**; 19:e0312947.

84. Klein JMA, Runge I, Pannen A-K, et al. Prevalence of bacterial vaginosis, sexually transmitted infections and their association with HPV infections in asymptomatic women attending antenatal care in Ethiopia. Ecancermedicalscience **2024**; 18:1783.

85. Rizzo A, Moschese D, Salari F, et al. Anal HPV prevalence in individuals with and without other concomitant sexually transmitted infections. J Med Virol **2024**; 96:e29852.

86. Zonta MA, Liljander A, Roque KB, et al. Prevalence of sexually transmitted infections and human papillomavirus in cervical samples from incarcerated women in São Paulo, Brazil: a retrospective single-center study. Front Public Health **2024**; 12:1353845.

87. Cameron JE, Rositch AF, Vielot NA, et al. Epstein-Barr Virus, High-Risk Human Papillomavirus and Abnormal Cervical Cytology in a Prospective Cohort of African Female Sex Workers. Sex Transm Dis **2018**; 45:666–672.

88. Sturt AS, Webb EL, Himschoot L, et al. Association of Female Genital Schistosomiasis With the Cervicovaginal Microbiota and Sexually Transmitted Infections in Zambian Women. Open Forum Infect Dis **2021**; 8:ofab438.

89. Rizzo A, Pozza G, Salari F, et al. Concomitant diagnosis of sexually transmitted infections and human monkeypox in patients attending a sexual health clinic in Milan, Italy. J Med Virol **2023**; 95:e28328.

90. Maldonado-Barrueco A, Sanz-González C, Gutiérrez-Arroyo A, et al. Sexually transmitted infections and clinical features in monkeypox (mpox) patients in Madrid, Spain. Travel Med Infect Dis **2023**; 52:102544.
