# Supplementary material for: Clinical Manifestations and Associated Disease States with Mycoplasma genitalium Infection: Narrative Review and State of the Literature, 2015–2025
Source: Open Forum Infect Dis. 2026 Mar 30;13(Suppl 2):S1339–51. doi: 10.1093/ofid/ofaf799 (PMC13035034; doi:10.1093/ofid/ofaf799)
Supplement: ofaf799_Supplementary_Data [file ofaf799_supplementary_data.zip › Mgen prevalence table_1212_clean.docx]

| **Supplementary Table 1: Studies Reporting Prevalence Data for *Mycoplasma genitalium* (MG), 2015-2025** | | | | |
| --- | --- | --- | --- | --- |
| **First Author, Year** | **Country** | **Setting** | **Population** | **Prevalence = % (95% CI)^a^, MG+ (n) / total (N)** |
| ***Studies Including Both Symptomatic and Asymptomatic Individuals*** | | | | |
| Napierala Mavedzenge, 2015[1] | Zimbabwe | Multiple centers for reproductive and general healthcare | Non-pregnant women with recent HIV^b^ seroconversion | Endocervical = 10.7%, 14/131 |
| Guschin, 2015[2] | Russia | STI^c^ clinic | Consecutive males at study site | Urethral = 15.9%, 51/320 |
| Hay, 2015[3] | South Africa | Multiple primary care clinics | Sexually active women | Vaginal = 8.7% (6.4-10.9), 52/601  Rectal = 2.7% (1.4-3.9), 16/601 |
| Justel, 2015[4] | Angola | Multiple obstetric wards | Pregnant women and their newborns | Endocervical = 6.1% (3.909.3), 19/312  Conjunctival = 0.8% (0.2-2.8), 2/255 |
| Peuchant, 2015[5] | France | Public hospital | Pregnant women | Vaginal = 0.8% (0.3-1.6), 8/1004  [18-24 vs >24 years, aOR^d^ = 9.0 (1.5-57.3, p=0.01)] |
| Chen, 2015[6] | China | Population survey with outreach in 13 cities | Males living with HIV infection | Urine = 20.1%, 310/1541  [Among PLWH^e^ CD4 cell <350 vs >350, 24.0% vs 15.3%, p<0.001] |
| Barreto Campos, 2015[7] | Brazil | Public health clinic | Women aged 14-78, (PLWH excluded) | Vaginal = 28.1%, 85/302 |
| Napierala, 2015[8] | United States | Aggregation of test results from a central lab | Males attending STI clinic or other outpatient clinics | Urine = 6.84%, 188/2750 |
| Vielot, 2015[9] | Kenya | Primary care clinic | Female sex workers | Endocervical = 16.8%, 59/350 |
| Deguchi, 2015[10] | Japan | STI clinic | Female sex workers | Vaginal = 14.1%, 21/149  Throat washing = 0.7% = 1/149 |
| Magaña-Contreras, 2015[11] | Mexico | Outpatient clinic | Women undergoing routine gyn screening | Endocervical = 0.5%, 1/201 |
| Homfray, 2015[12] | United Kingdon | National survey participants | Men aged 16-74 years | Urine = 1.9% (0.7-.51), 5/284 (circumcised)  Urine = 1.0% (0.6-1.7), 16/1566 (uncircumcised)  [Not sig] |
| Hook, 2015[13] | United States | Multiple STI clinics | Men and women with uncomplicated urogenital gonorrhea | Urogenital = 16.9%, 10/59 |
| Hay, 2015[14] | United Kingdom | Multiple universities | Non-pregnant female students aged ≤27 years | Urogenital (GU) = 3.2%, 64/2004 |
| Sonnenberg, 2015[15] | United Kingdom | National survey participants | Men and women aged 16-74 | Urine = 1.2%* (0.7-1.8), 24/1875 (men)  Urine = 1.3%* (0.9-1.9%), 48/2632 (women)  [Men Black ethnicity aOR 12.05 (3.68-39.44)] |
| van der Veer, 2016[16] | The  Netherlands | STI clinic | Men receiving care at study site | Urine = 3.1%, 37/1204 (overall)  Urine = 3.8%, 20/526 (MSW^f^)  Urine = 2.5%, 17/678 (MSM^g^)  [Not sig] |
| Oliphant, 2016[17] | New Zealand | Multiple STI clinics | Consecutive women who received STI screening | Endocervical = 8.4% (5.6-12.5), 22/261 |
| Fuchs, 2016[18] | Germany | Single center for anal cancer screening | MSM living with HIV infection | Rectal = 4.2% (2.6-6.6), 18/503 |
| Munson, 2016[19] | United States | Aggregation of test results from a central lab | Females undergoing chlamydia/gonorrhea testing | GU = 10.3%, 106/1032 (outpatient OB^h^)  GU = 15.2%, 7/46 (inpatient OB)  GU = 6.9%, 18/261 (suburban family care)  GU = 10.7%, 41/384 (urban family care)  GU = 14.6%, 110/755 (ED) |
| Paulsen, 2016[20] | Norway | Multiple clinics, including STI and non-STI clinics | Samples tested for chlamydia in people >15 years | Urine = 3.6%, 169/4665 |
| Molin, 2016[21] | Norway | STI clinic | Women receiving care at study site | Vaginal = 2.7%, 2847/103564 (overall)  Vaginal = 1%, 7/641 (WSW)  Vaginal = 3%, 346/12010 (WSWM)  Vaginal = 3%, 2494/90913 (WSM) |
| Ursi, 2016[22] | Belgium | STI clinic | Men and women receiving care at study site | Urine = 1.6%, 7/441 |
| Getman, 2016[23] | US | Multiple centers, including STI clinics | Men and women receiving care at study sites | GU = 16.1% (13.2-19.6), 83/515 (women)  GU = 17.2% (13.9-21), 74/431 (men) |
| Gesink, 2016[24] | Canada | STI clinic | Consecutive male, female, and transgender patients receiving care at study site | Urine = 4.2% (3.2-5.5), 50/1193 |
| Sylverken, 2016[25] | Ghana | STI clinic | Women receiving care at study site | Vaginal = 3.2%, 5/156 (symptomatic)  Vaginal = 6.8%, 3/44 (asymptomatic) |
| Djomand, 2016[26] | Namibia | Multiple outpatient health facilities | Males and females with recent HIV diagnosis | Vaginal = 12.8% (9.2-17.1), 39/305  Urine = 11.4% (7.7-16.0), 28/246 (males) |
| Munson, 2017[27] | United States | STI clinic | Men receiving care at study site | Urine = 6.6%, 97/1465  Pharyngeal = 0.9%, 13/1493  Rectal = 5.8%, 48/823 |
| Björnelius, 2017[28] | Sweden | Two STI clinics | Men and women receiving care at the study sites | GU = 7.5% (6.5-8.7), 171/2276 |
| Pereyre, 2017[29] | France | Multiple centers | Men and women receiving cares at sites served by central lab | All sites = 3.4% (2.8-4.2), 88/2594  Cervicovaginal = 3.0%, 45/1481 (women)  GU = 4.2%, 38/904 (men)  Sperm = 1.7%, 1/58  Throat = 1.7%, 1/59 (men)  Anal = 5.9%, 3/51 (men) |
| Lefebvre, 2017[30] | France | STI clinic | Male and female patients receiving care at study site | All sites = 2.4% (1.3-3.6), 16/651 |
| Le Roux, 2017[31] | South Africa | Single family practice clinic | Men receiving care at the study site | Urine = 15.7%, 59/375 (overall)  Urine = 13.7%, 41/300 (symptomatic)  Urine = 5.3%, 4/75 (asymptomatic)  [Sig more likely in symptomatic, p=0.002) |
| Dehghan Marvast, 2017[32] | Iran | Single infertility clinic | Sequential couples receiving care at study site | GU = 0%, 0/500 |
| Del Prete, 2017[33] | Italy | Aggregation of samples from clinics served by a central lab | Men and women tested for STIs | GU = 0.4%, 5/1272 (women)  GU = 1%, 3/303 (men) |
| Jenniskens, 2017[34] | The Netherlands | Multiple sites served by a central lab | Men and women with CT/NG^i^ screening | GU = 1.9%, 108/5628 |
| Tabrizi, 2017[35] | Australia | Multiple sites, including an STI clinic | Consecutive men and women with STI testing | All sites = 6.0%, 65/1089  GU = 3.3%, 23/678 (women)  Urine = 9.9%, 35/354 (men)  Anal = 20.6%, 7/34 (men) |
| Edouard, 2017[36] | France | Single center | Convenience sample, PLWH >18 years | Rectal = 1.7%, 2/118 |
| Gratrix, 2017[37] | Canada | Multiple STI clinics | Sequential males and females >17 years undergoing screening for NG/CT | GU = 6.2% (5.2-7.2), 142/2294 (overall)  GU = 5.3% (4.0-6.5), 64/1212 (men)  GU = 7.2% (5.6-8.8%), 75/1042 (women) |
| Chernesky, 2017[38] | Canada | Single health center for street youth | Consecutive men aged 16-25 attending study site for care | Urethral = 15.3%, 54/354  Urine = 12.6%, 45/356  [Urethral swabs sig more sensitive, p=0.035] |
| Edouard, 2017[39] | France | Single center | Men and women with STI testing at study site | All sites = 3.3%, 154/4624  Vaginal = 3.6%, 78/2163  Urine = 3.4%, 71/2070  Urethral = 3.5%, 5/126  Rectal = 0%, 0/90  Pharyngeal = 0%, 0/74  Sperm = 0%, 0/58 |
| Madsen, 2017[40] | Denmark | Multiple HIV clinics | Women living with HIV, age ≥18 | Endocervical =1.3%, 3/234 |
| Hakre, 2017[41] | United States | HIV clinic | US Air Force personnel living with HIV undergoing annual medical exam | All sites = 18.6%, 19/102 (overall)  Urine = 9.3% (4.3–16.9), 9/97  Rectal = 11.1% (5.7–19.0), 11/99  Pharyngeal = 0.98% (0.0–5.3), 1/102 |
| Leli, 2018[42] | Italy | Multiple sites | Women aged 15-50 receiving care at study sites | Vaginal = 0.5%, 9/1761 |
| Tjagur, 2018[43] | Estonia | Multiple departments at a single center | Heterosexual men aged 18-49.5 | Urine = 4.4%, 36/825 |
| Unemo, 2018[44] | Denmark, Norway, Sweden | Multiple STI clinics | Consecutive males and females | Vaginal = 8.2%, 138/1683  Cervical = 8.2%, 23/280  Urethral = 9.0%, 26/290 (females)  Rectal = 8.1%, 3/37 (females)  Pharyngeal = 0%, 0/74 (females)  Urine = 6.0%, 165/2740 (males)  Urethral = 7.4%, 56/754 (males)  Rectal = 6.3%, 15/237 (males)  Pharyngeal = 1.5%, 4/260 (males) |
| Dionne-Odom, 2018[45] | United States | HIV primary care clinic | MSM >18 years with receptive anal intercourse in last 30 days | All sites = 17.2%, 27/157  GU = 10.8%, 17/157  Rectal = 6.4%, 10/157  [No sig difference in organism load by anatomic site] |
| Upton, 2018[46] | New Zealand | Outpatient clinics of Aukland and Northland regions (population ~1.6 million) | All samples tested for CT/NG in individuals ≥13 years | All sites = 5.1%, 134/2643  [No sig difference between men and women (aOR 1.35, p=0.19); sig more prevalent in those aged <25 versus older (aOR 3.04, p<0.001) and Māori/Pacific vs European (aOR 1.82, p=0.002) or Asian (aOR 3.02, p=0.002)] |
| Balkus, 2018[47] | Kenya and United States | Multiple sites | Non-pregnant women aged 18-45 without HIV infection with 1 or more vaginal infection | Vaginal = 35%, 77/221  [Non-sig higher prevalence in Kenya vs US (38%, 64/168, and 25%, 13/53, respectively; p=0.07] |
| Lockhart, 2018[48] | Kenya | STI clinic | Female sex workers | Vaginal = 20.1%, 70/348 |
| Allan-Blitz, 2018[49] | United States | Remnant samples from a lab serving a large health system | Individuals with testing for CT/NG | All sites = 2.0% (1.1-3.8), 10/500 (overall)  Urine = 1.1%, 4/362  Rectal = 17.4%, 4/23  Vaginal = 1.9%, 2/106 |
| Chra, 2018[50] | Greece | STI clinic | Consecutive patients | GU = 5.7%, 10/176 |
| Couldwell, 2018[51] | Australia | STI clinic | MSM | All sites = 13.4%, 68/508  Urethra = 4.7%, 24/508  Rectal = 8.9%, 45/505  Pharyngeal = 0%, 0/508 |
| Trent, 2018[52] | United States | Multiple clinics in a large health system | Women aged 13-29 during prenatal and gynecological visits who underwent CT/NG testing | GU = 16%, 75/483 |
| Brosh-Nissimov, 2018[53] | Israel | Samples from a central lab serving the Israel Defense Force clinical sites | Israel Defense Force soldiers on active duty | All sites = 1.9%, 54/2816 |
| Trevis, 2018[54] | Australia | Opportunistic sampling at hostels | Backpackers | Urine = 1.8% (0.22-3.38), 5/271 |
| Kaida, 2018[55] | South Africa | Multiple sites of a longitudinal research cohort (AYAZAZI study) | Aged 16-24, not known to be living with HIV infection | GU = 6.8%, 24/352 (overall cohort)  Urine = 3.3%, 5/154 (males)  Vaginal = 9.6%, 19/198 (females) |
| Clifton, 2018[56] | United Kingdom | National survey participants (Natsal-3) | Men and women aged 16-74 | Urine = 1.6%, 72/4550 |
| Lillis, 2019[57] | United States | STI clinic | Non-pregnant women | GU = 17.5%, 70/400  [MG independently associated with age <25 (p<0.03) and with reporting ≥2 partners in last 12 months] |
| Nolskog, 2019[58] | Sweden | Multiple youth clinics | Patients aged 15-25 tested for CT | Urine = 9.8%, 96/977 (overall)  Urine = 7.8%, 39/499 (women)  Urine = 11.9%, 57/478 (men) |
| Hoffman, 2019[59] | South Africa | Mobile clinic | Women aged ≥18 | Vaginal = 8.4%, 21/251 |
| Ferré, 2019[60] | Togo | National, population-based survey | MSM ≥18, transgender and gender diverse excluded | Rectal = 15.0% (10.1-19.9), 31/207 |
| Mulligan, 2019[61] | Ireland | STI clinic | Consecutive patients | Urine = 3% (1.3-4.7), 12/400 |
| Ong, 2019[62] | South Africa | Cervical cancer screening study | Women living with HIV aged 25-50 | Endocervical = 7.4% (5.5-9.7), 46/622  [Women with MG were sig younger (mean 31.8 versus 35.2 years, p<0.001), had more lifetime sexual partners (4.3 vs 3.7, p<0.001)] |
| Deborde, 2019[63] | France | PrEP^j^ program | MSM | Overall = 10.1%, 9/89  Rectal = 57.5%, 15/26  Urethra = 34.6%, 9/26  Pharyngeal = 7.7%, 2/26 |
| Harrison, 2019[64] | Solomon Islands | Multiple antenatal clinics | Women | Vaginal = 11.9% (8.3-16.6), 28/236 |
| Ferré, 2019[65] | Togo | Population-based survey, recruiting in “hot-spots” and brothels | Female sex workers ≥18 | GU = 5.5% (2.9-8.0), 17/310 |
| Batteiger, 2019[66] | United States | STI clinic | Men ≥18 | Urine = 10.8%, 21/197  Rectal = 1.5%, 3/197 |
| Gaydos, 2019[67] | United States | Multiple sites including STI clinics (AMES study) | Females and males age ≥14 | GU = 10.1%, 176/1737 (females)  GU = 10.6%, 165/1563 (males) |
| de Salazar, 2019[68] | Spain | STI clinic | Patients receiving care at study site | Rectal = 4.8%, 4/83  Pharyngeal = 4.6%, 10/218  Endocervical = 3.7%, 4/107  Urine = 2.8%, 6/214 |
| Güralp, 2019[69] | Cyprus | Multiple sites, including inpatient gynecology unit | Non-pregnant women ≥18 | Endocervical = 2.9%, 8/273 |
| Stewart, 2020[70] | Australia | Multiple sites within a large healthcare network | Anyone tested for CT/NG during the study period | GU = 5%, 56/1176 (overall)  GU = 3%, 12/365 (men)  GU = 5%, 44/811 (women)  GU = 9%, 8/92 (pregnant women) |
| Spiller, 2020[71] | United Kingdom | Multiple STI clinics | Patients receiving care at study sites | GU = 2.7% (1.5-4.0), 17/600 (females)  GU = 3.5% (1.7-5.2), 13/400 (males) |
| Alicja, 2020[72] | Poland | Single hospital | Hospitalized patients with active or non-active systemic lupus erythematous | GU = 8.3%, 3/36 |
| Crowell, 2020[73] | Nigeria | HIV/STI treatment and prevention cohort (TRUST/RV368) | MSM and TGW^k^ ≥18 | Rectal = 36.8% (32.1-41.6), 150/408  GU = 12.4% (9.2-15.6), 51/410 |
| Smullin, 2020[74] | South Africa | Public health facility | Women ≥18 receiving antenatal care | Vaginal = 18%, 35/197 (overall)  Vaginal = 24% (16-34), 22/92 (PLWH)  Vaginal = 12% (6.8-20%), 13/105 (PWoH^l^) |
| Spornraft-Ragaller, 2020[75] | Germany | HIV/STI clinic | MSM living with HIV presenting for annual clinical visit | Rectal = 8.2%, 26/317 |
| Jansen, 2020[76] | Germany | Nationwide multi-center study, “MSM-friendly” clinics | MSM ≥18 | Overall = 17% (15.5-18.6), 66/391  Pharyngeal = 2.9% (0.2-3.0), 2/66  Rectal = 11.5% (10.2-12.9), 30/265  Urine = 5.4% (4.5-6.4), 7/124 |
| Bernier, 2020[77] | Russia | Respondent-driven sampling in Moscow | Women ≥18 receiving money, drugs, or goods in exchange for sex in the preceding 3 months | All sites = 14.9%^*^ (23.8-33.6), 54/385  Rectal = 7.4% (4.1-13.0), 18 cases  Pharyngeal = 0%, 0 cases  Vaginal = 13.0% (9.0-18.0), 48 cases |
| Khosropour, 2020[78] | United States | STI clinic | Non-pregnant women ≥16 with history of CT, partner with CT, or urogenital symptoms | Overall = 26%, 13/50  Vaginal = 20%, 10/50  Rectal = 22%, 11/50  [Report of anal sex in the past 12 months was not associated with rectal MG, p=0.47] |
| Manhart, 2020[79] | United States | Multiple sites including STI clinics (AMES study) | Females and males age ≥14 | GU = 10.1%, 176/1737 (females)  GU = 10.6%, 165/1563 (males)  [MG risk sig higher women aged 15-24 vs aged 35-49 (aOR 5.05, 3.01-8.46), men aged 15-24 vs 35-49 (aOR 1.91, 1.20-3.02), Black versus white race (aOR 1.88, 1.30-2.72 for women and aOR 2.02, 1.38-2.96 for men), and non-Hispanic vs Hispanic (aOR 1.97, 1.25-3.10 for women and aOR 1.80, 1.14-2.85 for men), and STI clinics, family planning clinics, public health clinics and EDs vs clinical research centers (aOR 2.71, 1.03-6.34)] |
| Park, 2020[80] | South Korea | Sexual assault centers | Female attendees of study sites | Vaginal = 7.9%, 146/1843 |
| Cowley, 2020[81] | Guinea Bissau | Fieldworkers directly outreaching to participants | Men and women aged 16-49 | GU = 1.9%, 9/478 (overall)  GU = 0.5%, 1/195 (males)  GU = 2.8%, 8/283 (females) |
| Grad, 2020[82] | Romania | Dermatology, infectious diseases, urology clinics | Men and women aged 17-75 | Urine = 3.4%, 7/207 (males)  Urine = 0%, 0/42 (females) |
| Hanna, 2020[83] | Lebanon | Multiple gynecology clinics | Non-pregnant women ≥18 | GU = 0.2%, 1/505 |
| Kharsany, 2020[84] | South Africa | HIV surveillance program, with referrals to local clinics and community organizations | Men and women ≥18 randomly selected from their household to participate in the study | Overall = 5.5% (4.8-6.1), 532/9778  Urine = 5.7% (4.8-6.7), 204/3529 (males)  Vaginal = 5.2% (4.5-6.0), 328/6249 (females) |
| Hilmarsdóttir, 2020[85] | Iceland | STI clinic | Males and females ≥18 | GU = 9.3%, 91/978 (overall)  Urine = 7.7%, 38/492 (males)  Vaginal = 10.9%, 53/487 (females) |
| Juliana, 2020[86] | Tanzania | Biobanked samples collected from multiple healthcare facilities | Pregnant women | Vaginal = 2.1% (0.9-3.9), 9/439 |
| Ng, 2020[87] | China | Multiple STI clinics | Female patients with endocervical swabs | Endocervical = 7.4%, 21/285 |
| Munson, 2020[88] | United States | Longitudinal cohort including MSM and TGW (RADAR) | Assigned male at birth, age 16-29, report of sexual encounter with a man in the last year | Urine = 9.1% (7.3-10.9), 179/1977  Rectal = 21.5% (19.1-23.8%), 423/1977 |
| Angela, 2020[89] | Italy | STI samples processed at a central lab | People with STI testing during the study period | All sites = 0.94%, 67/7150 |
| Karim, 2021[90] | Morocco | Single gynecology department | Consecutive women presenting for routine gynecologic exam or Pap smear | Endocervical = 1%, 8/809 |
| Balle, 2021[91] | South Africa | Youth center | Females 15-19 seeking hormonal contraception | Vaginal = 6.9%, 5/72 |
| Nijhuis, 2021[92] | The Netherlands | Single medical center | All consecutively received samples for STI testing | All sites = 4.1%, 106/2592 |
| Oh, 2021[93] | South Korea | Multiple clinics | Males and females receiving STI screening | All sites = 3.3%, 1942/59381 |
| Perin, 2021[94] | United States | Single academic medical center | Pregnant women aged 13-29 | Vaginal = 18.1%, 51/281 |
| Desdorf, 2021[95] | Denmark | Multiple primary care clinics served by a central lab | People receiving testing for CT/NG | Urine = 4.2%, 25/601 |
| Naicker, 2021[96] | South Africa | Single hospital | Pregnant women ≥18 | Urine = 5.9%, 13/221 |
| Rowlinson, 2021[97] | United States | STI clinic | Cisgender male STI clinic patients ≥16 reporting sex exclusively with female partners, PLWH excluded | Urine = 11.1%, 34/307  Urine = 5.0%, 10/200 (non-NGU^m^)  Urine = 22.4%, 24/107 (NGU)  [Sig higher prevalence among men with NGU, p<0.01] |
| Parmar, 2021[98] | Canada | Specimens received at a central lab | Patients tested for CT/NG | Urine = 9.6%, 189/1977 (overall)  Urine = 6.2%, 38/609 (females)  Urine = 11%, 151/1368 (males) |
| Calas, 2021[99] | France | STI clinic | All patients visiting the clinic | GU = 1.81%, 15/826  Pharyngeal = 0.33%, 2/606  Rectal = 2.36%, 3/127 |
| Lucena Nemirosky, 2021[100] | Spain | Multiple centers | Men and women with symptoms or reporting recent condomless sexual activity | GU = 3.4% (3.2–3.7), 612/17846 (overall)  GU = 2.9% (2.6–3.2), 381/13071 (females)  Urethral = 4.8% (4.3–5.5), 231/4775 (males) |
| Han, 2021[101] | China | Multiple STI clinics | Patients ≥18 | All sites = 7.2%, 97/1374  Rectal = 2.2%, 30/1377  GU = 5.6%, 77/1374  [Sig increased risk for rectal MG among MSM (aOR 40.4, 3.9-421.2), those with having genital symptoms (aOR 3.5, 1.2-10.5), and having infection with urogenital MG (AOR 7.2, 2.9–18.0], decreased risk for males vs females (aOR 0.2, 0.1-0.7)] |
| Torrone, 2021[102] | United States | National survey participants (NHANES) | People aged 14-59 | Urine = 1.7%, 67/3945  [MG prevalence sig higher among non-Hispanic Blacks vs non-Hispanic Whites (PR 2.5, 1.0–6.4), higher among persons reporting ≥10 lifetime partners vs 0-4 (PR 3.0, 1.2–7.4)] |
| Zhang, 2021[103] | China | STI clinic | Patients ≥18 in specific exam rooms | GU = 7.2%, 49/678 (overall)  GU = 7.4%, 36/489 (men)  Endocervical = 6.9%, 13/189 (women) |
| Scoullar, 2021[104] | Papua New Guinea | Five antenatal clinics | Women attending their first antenatal clinic visit | GU = 12.5% (10.0-15.3), 78/625 |
| Taku, 2021[105] | South Africa | Community based clinic | Stored cervical samples from women ≥30 | Endocervical = 1.5%, 3/205 |
| Jary, 2021[106] | Mali | HIV clinic | Non-pregnant women ≥18 receiving cervical cancer screening | GU = 9%, 13/144 (overall)  GU = 11%, 5/44 (PLWH)  GU = 8%, 8/96 (PWoH)  [No sig difference between PLWH and PWoH, p=0.55] |
| Llangarí-Arizo, 2021[107] | Ecuador | Public clinic | FSW^n^ ≥18 | Vaginal = 4.9% (2.8-8.4), 12/245 |
| Hammer, 2021[108] | United States | Aggregated specimens from cervical cancer screening covering 70% of state of New Mexico | Women aged 21-64 receiving cervical cancer screening | Vaginal = 1.95%^*^, 1651/84686 |
| Begnis, 2021[109] | France | STI clinic | All patients screening for CT/MG/NG | All sites = 4.88%, 101/2969  GU = 4.38 (3.51-5.40), 87/1987  Rectal = 3.06% (1.47-5.62), 10/327  Pharyngeal = 0.61 (0.25-1.27%), 69/1136 |
| Stafford, 2021[110] | United States | Remnant samples within a single healthcare system | Pregnant women with endocervical samples | Endocervical = 5.7%, 41/719  [MG sig more likely in younger women (mean 24.9 vs 28.1 years, p=0.004), more likely Black race (p=0.003) and Hispanic ethnicity (p=0.008)] |
| Horseman, 2021[111] | United States | A single military medical center | US military services members and their beneficiaries in the Pacific region | All sites = 8.8%, 165/1876 (overall)  All sites = 7.1%, 82/1158 (females)  All sites = 11.6%, 83/718 (males)  Urine= 7.6%, 37/484 (females)  Endocervical = 6.6%, 44/670  Urethral = 0%, (0/1) (females)  Vaginal = 33.3%, 1/3  Pharyngeal = 0%, 0/2 (females)  Urine = 11.3%, 75/665 (males)  Urethral = 13.6%, 3/22 (males)  Rectal = 28.6%, 4/14 (males)  Pharyngeal = 6.7%, 1/15 (males) |
| Kenyon, 2021[112] | Belgium | Be-PrEP-ared study of PrEP | MSM on PrEP | All sites = 39.1%, 79/179 (positive at some point during the study) |
| Beayni, 2021[113] | Lebanon | All STI tests performed at a central lab | All patients tested for STIs | GU = 4.5%, 26/597 |
| Tovo, 2021[114] | Burkina Faso | Single gynecology department | FSW | All sites = 11.54%, 21/182 |
| Martín-Saco, 2021[115] | Spain | Single hospital | Patients with STI testing | All sites = 2.8%, 234/8473 |
| Brin, 2022[116] | France | Multiple departments including an STI clinic from a single hospital | Consecutive patients tested for MG/CT/NG | All sites = 7%, 397/5586 (overall)  All sites = 8.6%, 319/3694 (men)  All sites = 4.1%, 78/1884 (women) |
| Shilling, 2022[117] | Australia | Women’s hospital | Women 15-45 tested for CT/MG receiving contraception or pregnancy termination services | GU = 3.4%, 599/17573 |
| Latimer, 2022[118] | Australia | STI clinic | Women ≥18 | GU = 6% (5%-8%), 83/1303  [No sig difference between asymptomatic (5%, 2-9) and symptomatic women (7%, 5-8), p=0.506] |
| Wang, 2022[119] | United States | Multiple clinics, including STI clinics | Non-pregnant, women aged 13-24, without HIV infection | Vaginal = 16.7% (12.0-22.4), 36/215 |
| Tu, 2022[120] | China | Public medical facility | Male and female (non-pregnant) living with HIV ≥18 | Urine = 4.7%, 19/406 |
| Liu, 2022[121] | China | OB/gyn department of a single hospital | Women aged 16-75 | GU = 3.1%, 100/3225 |
| Klavs, 2022[122] | Slovenia | Individuals identified from probability sample of general population | Individuals selected for CT testing | Urine = 0.4% (0.1-1.1), 4/1023 |
| Streeck, 2022[123] | Germany | Multiple sites (BRAHMS cohort) | MSM without HIV infection aged 18-55 | All sites = 19.0%, 198/1043  Rectal = 13.3%, 139/1043  Pharyngeal = 3.3%, 34/1043  GU = 6.0%, 63/1043 |
| Herms, 2022[124] | France | STI clinic | All patients with MG testing | All sites = 11%, 28/249 |
| Che, 2022[125] | China | Gynecology hospital | Non-pregnant women aged 18-50 | Endocervical = 2.6%, 119/4526 |
| Ring, 2022[126] | Switzerland | HIV clinics part of ZPHI cohort study | PLWH | All sites = 20.3%, 30/148 |
| Scaglione, 2022[127] | Italy | Single hospital | Anonymized samples collected for STI testing | GU = 0.3%, 2/717 |
| Asare, 2022[128] | South Africa | Clinical research sites of CAPRISA002 acute HIV infection study | Women ≥16 with new HIV infection (neg HIV Ab within 5 months) self-identifying as sex worker or as having >3 partners in preceding 3 months | GU = 8.6%, 15/175 |
| Skjælaaen, 2022[129] | Norway | Sexual assault center | Patients ≥14 | GU = 6.4%, 34/529 |
| De Baetselier, 2022[130] | Togo, Burkina Faso, Côte d’Ivoire, Mali | Multiple community clinics (CohMSM-PrEP study) | MSM ≥18 | All sites = 19.4%, 116/598 |
| Waniki, 2022[131] | Kenya | Multiple university and college campuses | MSM ≥18 | All sites = 6.0% (2.6-9.4)^*^, 25/242 |
| Sienkiewicz, 2023[132] | United States | University campus health clinics | Students presenting to campus clinic for STI care or to participate in the study | GU = 6.7%, 77/1156 |
| Hu, 2023[133] | United States | Multiple OB clinics | Women ≥18 receiving prenatal care | Vaginal = 8.0%, 18/224 |
| Perry, 2023[134] | Belgium, Germany, Spain, United Kingdom | Remnant samples from multiple sites, including STI clinics | Patients ≥18 who received CT/NG screening | Urine = 1.7% (0.9-3.1), 9/538 (females)  Vaginal = 5.7% (3.7-8.8), 19/332  Endocervical = 2.6% (1.3-5.15), 8/304  Rectal = 5.8% (2.5-12.9), 5/86 (females)  Urine = 3.9% (3.0-5.2), 46/1168 (males)  Rectal = 12.5% (9.5-16.3), 46/367 (males) |
| Zhang, 2023[135] | China | Multiple gynecologic clinics | Non-pregnant women ≥18 | Vaginal = 3.4%, 74/2190 |
| Maldonado-Barrueco, 2023[136] | Spain | Tests screened for MG at a central lab in Madrid | Males and females with MG screening | All sites = 2.1%, 56/2654 |
| Shipitsyna, 2023[137] | Guatemala, Malta, Morocco, Peru, South Africa | Multiple STI clinics, part of WHO initiative global project on point-of-care tests for STIs | Consecutive patients receiving STI care at study sites | Urine = 4.3%, 60/1384 (MSM)  Rectal = 12.4%, 173/1397 (MSM)  Vaginal = 19.1%, 259/1359 (women)  [MG sig more prevalent in rectal than urine samples among MSM, p<0.0001] |
| Sandri, 2023[138] | Italy | STI clinic | MSM | All sites = 9.2%, 96/1040 |
| de Jesus Salgado, 2023[139] | Brazil | Referrals to the study through various outreach strategies | MSM and TGW aged 15-19 | All sites = 5.7%, 14/246  Pharyngeal = 3.3%, 8/245  Rectal = 2.9%, 5/210  Urethral 1.4%, 3/213 |
| Yusuf, 2023[140] | The Netherlands | Multiple primary care clinics | Patients tested for STIs | All sites = 6.7% (6.2-7.4), 497/7411 (females)  All sites = 3.7% (3.3-4.3), 212/5732 (males) |
| Govender, 2023[141] | South Africa | Multiple primary care clinics | Pregnant women receiving antenatal care, without HIV infection | Vaginal = 3.5% (2.4-5.1), 26/752 |
| Menezes, 2023[142] | United States | Adolescent medicine subspeciality clinics within 1 health system | Females aged 13-20 where CT/NG testing was recommended | GU = 11.1%, 17/153 |
| Manhart, 2023[143] | United States | STI clinics in 6 US cities | Patients in care at study sites | All sites = 16.6% (14.9-18.5), 290/1743 |
| Totten, 2023[144] | United States | Stored samples from a cohort of pregnant women served by a local healthcare system | Pregnant women | GU = 11.3% (6.9-15.7), 23/203 |
| Butcher, 2023[145] | The Gambia | Single center, archived specimens used | Women aged 15-69 enrolled in a trial of HPV vaccine schedules | Urine = 0.2% (0.0-1.3), 1/420 |
| Lokken, 2023[146] | Kenya | Multiple hospitals serving as study sites for the Microbiota and Preterm Birth (MPTB) study | Women 18-45 without HIV infection trying to conceive | Vaginal = 7.7%, 30/389 |
| Amesty, 2023[147] | Dominican Republic | Multiple sites, including HIV/STI clinics | Recruitment focused on marginalized groups: PLWH aged 18-60, FSW aged 18-60, pregnant youth aged 15-24, individuals coming from rural areas, MSM, and TGW | GU = 2.3%, 46/1991 (overall)  GU = 1.7%, 7/408 (pregnant youth)  Urine = 5.0%, 14/278 (MSM)  GU = 0%, 0/101 (TGW)  GU = 2.8%, 11/386 (rural)  FSW = 2.3%, 9/394 (FSW) |
| Harryparsad, 2023[148] | South Africa | Sub-study of the ECHO trial | Non-pregnant women aged 16-35 seeking effective contraception | Vaginal = 4%, 7/162 |
| Valasoulis, 2023[149] | Greece | Outpatient gynecology clinics, including colposcopy clinics | Non-pregnant women undergoing cervical cancer screening | Endocervical = 0.65%, 14/2156 |
| Filho, 2023[150] | Brazil | Single tertiary hospital providing prenatal care | Pregnant women aged 15-45 | Vaginal = 10%, 20/200 |
| Lê, 2023[151] | Canada | Longitudinal cohort involving community-based organizations (Engage Cohort Study) | MSM ≥16 | All sites = 5.7% (4.0-8.1), 41/716  Rectal = 4.0%^*^ (2.6-6.0), 41/688  Urethral swab = 2.2%^*^ (1.2-4.0), 23/687  Pharyngeal = 0.2%^*^ (0.1-0.9), 2/688 |
| Lindman, 2024[152] | Guinea-Bissau | Multiple venues known for sex work | FSW | Vaginal = 21.9%, 69/315 |
| Romo, 2024[153] | United States | US military health care clinic | Active-duty US military and beneficiaries of the Military Health System aged 18-30 | Urine = 10.0%, 43/432 (overall)  Urine = 13.5%, 23/170 (women)  Urine = 7.6%, 20/262 (men) |
| Beesham, 2024[154] | South Africa | Longitudinal study (ZINK) offering PrEP | Women aged 18-35 without HIV infection | Vaginal = 2%, 1/50 |
| Miranda, 2024[155] | Brazil | Public antenatal clinics | Women aged 15-49 | Vaginal = 7.8% (6.8-8.8), 212/2728 |
| Lavey, 2024[156] | United States | University health clinic | University students undergoing STI screening | GU = 6.51%, 76/1167 |
| Ljubin-Sternak, 2024[157] | Croatia | Primary care clinics | Men ≥18 receiving testing for STIs | Urine = 2.2%, 145/6480 |
| Tsai, 2024[158] | Taiwan | Single hospital | PLWH, people with STIs in the last 6 months, people on PrEP, people seeking STI care | All sites = 8.1%, 83/1021  PLWH = 10.2%, 54/531  PWoH = 5.9%, 29/490  [MG sig more likely among PLWH, p=0.01] |
| Lam, 2024[159] | Vietnam | STI clinic | All patients receiving care at study site | GU = 5.95% (4.76-7.14), 90/1513 (overall)  GU = 4.82% (3.49-6.14), 49/1017 (women)  GU = 8.27% (5.83-10.69), 41/596 (men) |
| Scoullar, 2024[160] | Papua New Guinea | Five antenatal clinics | Women attending their first antenatal clinic visit | Vaginal = 13.9%, 52/373 |
| Kadylak, 2024[161] | Poland | STI clinic | Males aged ≥16 | All sites = 8.6% (4-13.3), 12/139 (overall)  All sites = 10.4% (4.6-16.2), 11/106 (MSM)  All sites = 3.2% (0-9.4), 1/31 (non-MSM)  Urine = 7.5% (2.5-12.6), 8/106 (MSM)  Pharyngeal = 0.7% (0-2.1), 1/106 (MSM)  Rectal = 1.9% (0-4.5), 2/106 (MSM)  Urine = 3.2% (0-9.4), 1/31 (non-MSM) |
| Nguyen, 2024[162] | Vietnam | STI/PrEP clinic | MSM on PrEP | Overall = 10.9%, 52/477  Urine = 3.2%, 15/476  Rectal = 7.3%, 34/464  Pharyngeal = 1.9%, 9/476 |
| Kostera, 2024[163] | United States | Non-sequential remnant samples | Women undergoing routine cervical cancer screening | Endocervical = 17.6%, 3/17^@^ |
| Hakre, 2024[164] | United States | HIV clinic | Male US Air Force personnel living with HIV undergoing annual medical exam | Overall = 19.7% (15.5-24.5), 59/299)  Urine = 6.7% (18/268)  Rectal = 13.9%, 41/294)  Pharyngeal = 1.7%, 5/296) |
| Bjartling, 2024[165] | Sweden | STI clinic | Consecutive MSM receiving care who received CT/NG testing | Overall = 10.5%, 64/609  Urine = 3.9%, 24/609  Rectal = 7.6%, 46/609  [Sig more likely to be positive from rectal swab, p=0.007] |
| Zonta, 2024[166] | Brazil | Single center (women’s prison) | Women prisoners undergoing cervical cancer screening | Endocervical = 3%, 9/298 |
| Llangarí-Arizo, 2024[167] | Ecuador | Candidates approached by research staff in brothels and non-sex workers approached in primary care clinics | Female sex workers (FSW), and a comparative group of non-sex workers (NSW) | FSW, all sites = 10.0% (6.8-14.5), 24/239 (overall)  Vaginal = 6.4% (4.0-10.1), 16/250  Pharyngeal = 0.4% (0-2.2), 1/249 (FSW)  Rectal = 6.7% (4.2-10.6), 16/239 (FSW)  NSW, all sites = 3.2% (1.6-6.2), 8/250 (overall)  Vaginal = 3.2% (1.6-6.2), 8/250 (NSW)  Pharyngeal = = 0.4% (0-2.2), 1/250 (NSW) |
| Wang, 2024[168] | China | Multiple STI, urology, and gynecology clinics in Shenzhen | Age ≥16 years receiving STI services | Urethral = 2.8% (2.3-3.6), 70/2466 (males)  Endocervical = 2.3% (1.9-2.7), 122/5420 |
| Sethi, 2024[169] | Cameroon | Multiple sites | Women attending prenatal and other routine visits | Endocervical = 5.2%, 35/680 |
| Proudmore, 2024[170] | Australia | Multiple sites, including STI clinics | Individuals undergoing STI screening | All sites = 12.6%, 1540/12178  Correctional facilities = 21.3%, 660/3103  Antenatal = 7.2%, 127/1767  ID clinics = 7.2%, 193/2695 |
| Rizzo, 2024[171] | Italy | STI clinic | People undergoing anal HPV^o^ testing (97% men) | Rectal = 5%, 24/507 |
| Baiers, 2024[172] | United States | Longitudinal cohort study (RADAR) | Sexual and gender minorities, assigned male at birth | Rectal = 20.8% (18.4-23.5), 213/1022 |
| Tian, 2024[173] | China | STI clinic | First time patients receiving care at study site | All sites = 7.4%, 350/4719 |
| Sheele, 2024[174] | United States | Multiple sites in a single health system | All patients in a single health system tested for MG | All sites = 1.8%, 103/5572  Outpatient = 1.8%, 96/5439  ED = 6.3%, 6/96  Inpatient = 2.7%, 1/37 |
| Oliveira, 2024[175] | Portugal | Single center | Men and women aged 18-64 | GU = 2.3%, 11/475 (overall)  GU = 3.4%, 6/176 (men)  GU = 1.7%, 5/299 (women) |
| Schröder, 2025[176] | Zambia | Multiple antenatal care facilities | Women receiving antenatal care, at least 13 weeks gestation | Vaginal = 12.6% (10.7-14.8), 127/1005 |
| Kogler, 2025[177] | Austria | STI clinic | Patients ≥18 who received 1 rectal and/or 1 pharyngeal swab during study period | Pharyngeal = 0.2%, 1/418  Urethra = 5.2%, 22/421  Vaginal = 0%, 0/16  Endocervical = 5.6%, 4/72  Rectal = 1.7%, 4/241 |
| Leng, 2025[178] | China | Single center | Men and women >18 years old | All sites = 13.0%, 21/162  Urethral = 6.1%, 8/132  Pharyngeal = 9.7%, 12/124  Rectal = 7.9%, 7/89 |
| Nguyen, 2025[179] | Vietnam | STI clinic | MSW attending the study site | Urethral = 14.6%, 307/2102 |
| Fortas, 2025 | Madagascar | Multiple outpatient clinics | Non-pregnant women aged 18-45 | Vaginal = 8.2% (5.8-10.6), 41/501 |
| Kerschberger, 2025[180] | Eswatini | Multiple outpatient clinics | Men and women receiving care at study sites | Urine = 10.5%, 77/735 |
| Santana, 2025[181] | Brazil | Single center | Pregnant women attending prenatal visits | Endocervical = 1.22%, 2/164 |
| ***Studies Including Only Symptomatic Individuals*** | | | | |
| Ito, 2016[182] | Japan | Single urology clinic | Men with symptomatic urethritis | Urine = 10.6%, 45/424 (overall)  Urine = 22.7%, 35/154 (those with non-CT/NG infection) |
| Saigal, 2016[183] | India | STI clinic | Men and women with urethral or vaginal discharge | GU = 1.2%, 2/164 |
| Dumke, 2016[184] | Germany | Single center | Men with symptoms of urethritis | Urethral = 5.9%, 19/323 |
| Moi, 2017[185] | Norway | STI clinic | Men with genital symptoms | Urethral = 3.8%, 1674/44180 |
| Le, 2017[186] | Japan | Multiple urology clinics | Men with urethritis | Urine = 8.7%, 8/92  Oral wash fluids = 0%, 0/92 |
| Rietmeijer, 2018[187] | Zimbabwe | Multiple STI clinics | Men with urethral discharge | Urine = 3.5%, 7/200 |
| Kufa, 2018[188] | South Africa | Multiple STI clinics | Women aged ≥18 | GU = 8.6%, 65/757  GU = 9.8%, 59/600 (age <35)  GU = 3.8%, 6/157 (age ≥35)  [Sig higher prevalence in age <35, p=0.017] |
| Mahlangu, 2019[189] | South Africa | Primary care clinic | Consecutive patients with male urethritis or vaginal discharge | Urine = 8.9%, 222/2509 (men)  Endocervical = 10.6%, 231/2222 (women) |
| McIver, 2019[190] | Australia | Multiple STI cilnics | Consecutive men ≥16 with symptoms of NGU | Urine = 12.8%, 75/588 (overall, same % in MSM and MSW) |
| Kato, 2020[191] | Japan | HIV clinic | MSM without symptoms of proctitis | Rectal = 4.4%, 4/90  Urine = 3.1%, 3/97 |
| Dhawan, 2020[192] | India | STI clinic | MSM with urethritis symptoms | All sites = 41.3%, 19/46  Rectal = 28.3%, 13/46  Pharyngeal = 0%, 0/7  Urine = 19.6%, 9/46 |
| Bachmann, 2020[193] | United States | Multiple STI clinics | Men ≥18 with symptomatic urethritis | GU = 28.7% (23.8-33.6), 262/914  [MG associated with Black race (aOR 2.41, 1.45-4.02) and younger age (aOR 0.95, 0.94-0.97)] |
| Broad, 2021[194] | United Kingdom | STI clinic | Men and women ≥16 | All sites = 11.1%, 61/550 (overall)  Vaginal = 7.5% (5.0-11.0), 23/307  Urine = 17.3% (12.4-23.7), 30/173 (MSW)  All sites = 11.4% (5.0-11.0), 8/70 (MSM) |
| Vives, 2021[195] | Spain | STI clinic | Men with genital symptoms | Urethral = 6.3%, 10/158 |
| Xie, 2021[196] | China | Single center (referral hospital) | Non-pregnant women ≥18 diagnosed with “chronic papillary urethritis” | Urine = 25.2%, 37/147 |
| Trent, 2021[197] | United States | Multiple sites within a single academic medical center | Women aged 13-25 with mild or moderate PID^p^ | Vaginal = 45%, 24/54 |
| Brehony, 2022[198] | Ireland | Maternity hospital | Symptomatic women ≥18 | GU = 0.77%, 10/1291 |
| Braam, 2022[199] | The Netherlands | STI clinic | Men with urethritis | Urine = 23%, 522/2288 |
| Lee, 2022[200] | China | STI clinic | Men with symptomatic NGU | Urine = 7.7%, 38/493 |
| Melendez, 2022[201] | Uganda | Government health centers | Men with symptomatic urethritis | Urethral = 12.8%, 32/250 |
| Nguyen, 2022[202] | Vietnam | STI clinic | Men aged 15-65 with symptomatic urethritis | Urethral = 19.5%, 68/349 |
| Hamill, 2022[203] | Uganda | Government health centers | Men with self-reported symptomatic urethritis | Urethral = 12.4% (8.1-16.5), 31/250 |
| Kazemian, 2022[204] | Iran | Multiple OB/gyn clinics | Women receiving care at study sites | Vaginal = 3.6%, 6/169 |
| Aboud, 2023 | Tanzania | Multiple STI clinics | Patients presenting with genital discharge or genital ulcerations | GU = 4.7%, 53/1134 |
| Kirkoyun Uysal, 2023[205] | Turkey | Single center | Urogenital specimens from symptomatic male and female patients | GU = 7.8%, 28/360 |
| Msemwa, 2023[206] | Tanzania | Multiple health facilities served by a central lab | Stored urine samples from people with urinary symptoms and pyuria with a negative urine culture | Urine = 5.7%, 13/227 (note: mid-stream urine samples) |
| Berti, 2023[207] | France | Single center (proctology clinic) | Consecutive patients with symptomatic proctitis | Rectal = 15%, 46/315 |
| Omosa-Manyonyi, 2023[208] | Kenya | Multiple outpatient facilities participating in a larger study | Women aged 18-50 with genital symptoms, without HIV infection | Vaginal = 5.6%, 43/813 |
| Llaca-Díaz, 2023[209] | Mexico | Urology department at a hospital | Men >18 with genital symptoms | Urine = 2%, 4/200 |
| Bazzo, 2024[210] | Brazil | Primary care clinics | Men >18 with penile discharge and/or genital ulcerations | Urethral = 8.2%, 94/1141 |
| Dunaiski, 2024[211] | Namibia | Single hospital providing gynecologic and OB services | Women aged 18-49 presenting with vaginal discharge | Vaginal = 15.6%, 17/109 |
| Manjate, 2024[212] | Mozambique | Primary care clinics | Women aged 18-49 with urogenital complaints | GU = 2.1% (1.0-3.0), 19/924 |
| Sukhija-Cohen, 2024[213] | United States | STI clinic | Adult male patients with penile discharge | Urine = 11.2%, 11/98 |
| Schwebke, 2024[214] | United States | Multiple centers | Women ≥14 with symptoms of vaginitis | Vaginal = 8.8%, 93/1051 |
| Vodstrcil, 2025[215] | Australia | STI clinic | Women with vaginal, pelvic, and/or abdominal symptoms | GU = 6.4% (4.8-8.4), 48/747 |
| Kanu, 2025[216] | Sierra Leone | Single center | Women with signs or symptoms of genital infections | Endocervical and vaginal = 0%, 0/104 |
| Tandon, 2025[217] | India | Multiple clinics | Women with symptomatic genital infections | Vaginal = 1.2%, 3/250 |
| ***Studies Including Only Asymptomatic Individuals*** | | | | |
| Kim, 2018[218] | South Korea | Gynecologic outpatient clinic | Asymptomatic women aged 21-80 receiving care at study site | Endocervical = 1.9%, 5/264 |
| Lima, 2018[219] | Brazil | General gynecology clinic and colposcopy clinic | Non-pregnant women without genital symptoms | Endocervical = 28.6%, 36/126 (overall)  Endocervical = 28.6%, 16/56 (general gyn)  Endocervical = 28.6%, 20/70 (colposcopy) |
| Read, 2019[220] | Australia | STI clinic | MSM ≥18 | Overall = 9.5% (7.7-11.5), 95/1001  Urine = 2.7% (1.8-3.9), 27/1001  Rectal = 7.0% (5.5-8.8), 70/1001  [MG sig associated with younger age (OR 0.96, 0.93-0.99, per year increase age), receptive anal sex with ≥2 partners in the last 3 months (OR 1.88, 1.09-3.3), and inconsistent condom use (OR 2.36, 1.24-4.81)] |
| Fernández-Huerta, 2019[221] | Spain | STI clinic | Asymptomatic individuals positive for CT and/or NG | GU = 4.8%, 12/249 |
| Nodjikouambaye, 2019[222] | Chad | Community outreach with subsequent visit to STI clinic | Asymptomatic women >18 | Vaginal = 1.6% (0.04-3.1), 4/251 |
| Fernández-Huerta, 2020[223] | Spain | STI clinic | Asymptomatic individuals positive for CT and/or NG | Overall = 7.4% (5.8-9.3), 66/890  Vaginal = 3.2% (1.3-6.4), 7/222  Rectal = 6.9% (1.9-16.7), 4/58 (women)  Urethral = 5.6% (2.7-10.0), 10/179 (MSW)  Urethral = 1.8% (0.8-3.5), 9/489 (MSM)  Rectal = 8.5% (6.1-11.4), 40/471 (MSM) |
| Bradley, 2020[224] | Australia | STI clinic | MSM ≥18 asymptomatic | Rectal = 7.0% (5.3-9.1), 52/742  [Sig higher in those on PrEP than not, 11.8% vs 5.6%, p=0.01] |
| Berçot, 2021[225] | France | Multiple hospitals participating in open-label phase of trial (ANRS IPERGAY) | Asymptomatic MSM taking TDF/FTC “on demand” and randomized to open-label “doxy-PEP” | All sites = 10.5%, 22/210 (baseline prevalence)  Urine = 6.3%, 13/205  Rectal = 4.3%, 8/184  Pharyngeal = 0.5%, 1/197 |
| Gragg, 2021[226] | United States | Single emergency department | Non-pregnant women aged 16-29 without genital symptoms | Vaginal = 14.8%, 23/155 |
| Suehiro, 2021[227] | Brazil | Convenience sample at a single university | Non-pregnant female students or employees of the university aged 18-50 | GU = 0.5%, 1/210 |
| Ando, 2021[228] | Japan | HIV and STI clinics | Asymptomatic MSM ≥16 | All sites = 6.1%, 60/982 (overall)  PLWH = 7.5%, 36/483  PWoH = 4.8%, 24/499  [No sig difference between PLWH and PWoH, p=0.083. MG sig more prevalent in rectal than GU samples, (4.7%, 46/977, vs 1.4%, 14/980, p< 0.0001] |
| Corgie, 2022[229] | France | Single medical center | Male and female aircrew >18, asymptomatic, with >15 leukocytes/hpf in urine sample | GU = 3.2%, 3/94 |
| Lesiak-Markowicz, 2022[230] | Austria | Leftover samples from soldiers leaving for or returning from missions abroad | Asymptomatic soldiers | Urine = 0%, 0/654 |
| Dias, 2024[231] | South Africa | STI clinic | MSM ≥18 reporting anal intercourse in the last 3 months | Rectal = 5.5%, 5/91  Pharyngeal = 0%, 0/97 |
| Klein, 2024[232] | Ethiopia | Multiple sites providing antenatal care | Pregnant women | Vaginal = 1.0%, 8/779 |
| Mukavhanyedzi, 2024[233] | South Africa | Single center | Pregnant women in care at study site | Vaginal = 2.9%, 6/202 |
| Sánchez Navarro, 2025[234] | Mexico | HIV clinic | MSM living with HIV ≥18 years | Urethral = 4.2%, 11/261 |

^a^: 95% CI = 95% confidence interval; ^b^: HIV = human immunodeficiency virus; ^c^: STI = sexually transmitted infection; ^d^: aOR = adjusted odds ratio; ^e^: PLWH = person living with HIV infection; ^f^: MSW = men who have sex with women; ^g^: MSM = men who have sex with male partners; ^h^: OB = obstetrics; ^i^: CT/NG = Chlamydia trachomatis / Neisseria gonorrhea; ^j^: PrEP = pre-exposure prophylaxis (to prevent HIV infections); ^k^: TGW = transgender women; ^l^: PWoH = person without HIV infection; ^m^: NGU = non-gonococcal urethritis; ^n^: FSW = female sex workers; ^o^: HPV = human papilloma virus; ^p^: PID = pelvic inflammatory disease; *weighted/adjusted prevalence; ^@^results are investigational, the Alinity m STI assay is not cleared to release MG results from ThinPrep specimens

References:

1. Napierala Mavedzenge S, Müller EE, Lewis DA, Chipato T, Morrison CS, Weiss HA. Mycoplasma genitalium is associated with increased genital HIV type 1 RNA in Zimbabwean women. J Infect Dis **2015**; 211:1388–1398.

2. Guschin A, Ryzhikh P, Rumyantseva T, Gomberg M, Unemo M. Treatment efficacy, treatment failures and selection of macrolide resistance in patients with high load of Mycoplasma genitalium during treatment of male urethritis with josamycin. BMC Infect Dis **2015**; 15:40.

3. Hay B, Dubbink JH, Ouburg S, et al. Prevalence and macrolide resistance of Mycoplasma genitalium in South African women. Sex Transm Dis **2015**; 42:140–142.

4. Justel M, Alexandre I, Martínez P, et al. Vertical transmission of bacterial eye infections, Angola, 2011-2012. Emerg Infect Dis **2015**; 21:471–473.

5. Peuchant O, Le Roy C, Desveaux C, et al. Screening for Chlamydia trachomatis, Neisseria gonorrhoeae, and Mycoplasma genitalium should it be integrated into routine pregnancy care in French young pregnant women? Diagn Microbiol Infect Dis **2015**; 82:14–19.

6. Chen L-S, Wu J-R, Wang B, et al. Epidemiology of Mycoplasma acquisition in male HIV-1 infected patients: a multistage cross-sectional survey in Jiangsu, China. Epidemiol Infect **2015**; 143:3327–3334.

7. Campos GB, Lobão TN, Selis NN, et al. Prevalence of Mycoplasma genitalium and Mycoplasma hominis in urogenital tract of Brazilian women. BMC Infect Dis **2015**; 15:60.

8. Napierala M, Munson E, Wenten D, et al. Detection of Mycoplasma genitalium from male primary urine specimens: an epidemiologic dichotomy with Trichomonas vaginalis. Diagn Microbiol Infect Dis **2015**; 82:194–198.

9. Vielot N, Hudgens MG, Mugo N, Chitwa M, Kimani J, Smith J. The Role of Chlamydia trachomatis in High-Risk Human Papillomavirus Persistence Among Female Sex Workers in Nairobi, Kenya. Sex Transm Dis **2015**; 42:305–311.

10. Deguchi T, Yasuda M, Horie K, et al. Drug resistance-associated mutations in Mycoplasma genitalium in female sex workers, Japan. Emerg Infect Dis **2015**; 21:1062–1064.

11. Magaña-Contreras M, Contreras-Paredes A, Chavez-Blanco A, Lizano M, De la Cruz-Hernandez Y, De la Cruz-Hernandez E. Prevalence of sexually transmitted pathogens associated with HPV infection in cervical samples in a Mexican population. J Med Virol **2015**; 87:2098–2105.

12. Homfray V, Tanton C, Miller RF, et al. Male Circumcision and STI Acquisition in Britain: Evidence from a National Probability Sample Survey. PLoS One **2015**; 10:e0130396.

13. Hook EW, Golden M, Jamieson BD, et al. A Phase 2 Trial of Oral Solithromycin 1200 mg or 1000 mg as Single-Dose Oral Therapy for Uncomplicated Gonorrhea. Clin Infect Dis **2015**; 61:1043–1048.

14. Hay PE, Kerry SR, Normansell R, et al. Which sexually active young female students are most at risk of pelvic inflammatory disease? A prospective study. Sex Transm Infect **2016**; 92:63–66.

15. Sonnenberg P, Ison CA, Clifton S, et al. Epidemiology of Mycoplasma genitalium in British men and women aged 16–44 years: evidence from the third National Survey of Sexual Attitudes and Lifestyles (Natsal-3). Int J Epidemiol **2015**; 44:1982–1994.

16. van der Veer C, van Rooijen MS, Himschoot M, de Vries HJC, Bruisten SM. Trichomonas vaginalis and Mycoplasma genitalium: age-specific prevalence and disease burden in men attending a sexually transmitted infections clinic in Amsterdam, the Netherlands. Sex Transm Infect **2016**; 92:83–85.

17. Oliphant J, Azariah S. Pelvic inflammatory disease associated with Chlamydia trachomatis but not Mycoplasma genitalium in New Zealand. Sex Health **2016**; 13:43–48.

18. Fuchs W, Kreuter A, Hellmich M, et al. Asymptomatic anal sexually transmitted infections in HIV-positive men attending anal cancer screening. Br J Dermatol **2016**; 174:831–838.

19. Munson E, Bykowski H, Munson KL, et al. Clinical Laboratory Assessment of Mycoplasma genitalium Transcription-Mediated Amplification Using Primary Female Urogenital Specimens. J Clin Microbiol **2016**; 54:432–438.

20. Paulsen LK, Dahl ML, Skaare D, Grude N. Prevalence of M. genitalium and U. urealyticum in urine tested for C. trachomatis. Tidsskr Nor Laegeforen **2016**; 136:121–125.

21. Molin S-B, De Blasio BF, Olsen AO. Is the risk for sexually transmissible infections (STI) lower among women with exclusively female sexual partners compared with women with male partners? A retrospective study based on attendees at a Norwegian STI clinic from 2004 to 2014. Sex Health **2016**; 13:257–264.

22. Ursi D, Crucitti T, Smet H, Ieven M. Evaluation of the Bio-Rad Dx CT/NG/MG® assay for simultaneous detection of Chlamydia trachomatis, Neisseria gonorrhoeae and Mycoplasma genitalium in urine. Eur J Clin Microbiol Infect Dis **2016**; 35:1159–1163.

23. Getman D, Jiang A, O’Donnell M, Cohen S. Mycoplasma genitalium Prevalence, Coinfection, and Macrolide Antibiotic Resistance Frequency in a Multicenter Clinical Study Cohort in the United States. J Clin Microbiol **2016**; 54:2278–2283.

24. Gesink D, Racey CS, Seah C, et al. Mycoplasma genitalium in Toronto, Ont: Estimates of prevalence and macrolide resistance. Can Fam Physician **2016**; 62:e96-101.

25. Sylverken AA, Owusu-Dabo E, Yar DD, et al. Bacterial etiology of sexually transmitted infections at a STI clinic in Ghana; use of multiplex real time PCR. Ghana Med J **2016**; 50:142–148.

26. Djomand G, Schlefer M, Gutreuter S, et al. Prevalence and Correlates of Genital Infections Among Newly Diagnosed Human Immunodeficiency Virus-Infected Adults Entering Human Immunodeficiency Virus Care in Windhoek, Namibia. Sex Transm Dis **2016**; 43:698–705.

27. Munson E, Wenten D, Jhansale S, et al. Expansion of Comprehensive Screening of Male Sexually Transmitted Infection Clinic Attendees with Mycoplasma genitalium and Trichomonas vaginalis Molecular Assessment: a Retrospective Analysis. J Clin Microbiol **2017**; 55:321–325.

28. Björnelius E, Magnusson C, Jensen JS. Mycoplasma genitalium macrolide resistance in Stockholm, Sweden. Sex Transm Infect **2017**; 93:167–168.

29. Pereyre S, Laurier Nadalié C, Bébéar C, investigator group. Mycoplasma genitalium and Trichomonas vaginalis in France: a point prevalence study in people screened for sexually transmitted diseases. Clin Microbiol Infect **2017**; 23:122.e1-122.e7.

30. Lefebvre M, Coutherut J, Gibaud S, et al. Prevalence of Mycoplasma genitalium Infection and Relationship with Symptoms Among Adults Attending a Sexual Health Centre. Acta Derm Venereol **2017**; 97:543–545.

31. le Roux MC, Hoosen AA. Quantitative Real-Time Polymerase Chain Reaction for the Diagnosis of Mycoplasma genitalium Infection in South African Men With and Without Symptoms of Urethritis. Sex Transm Dis **2017**; 44:17–20.

32. Dehghan Marvast L, Aflatoonian A, Talebi AR, Eley A, Pacey AA. Relationship between Chlamydia trachomatis and Mycoplasma genitalium infection and pregnancy rate and outcome in Iranian infertile couples. Andrologia **2017**; 49.

33. Del Prete R, Ronga L, Lestingi M, et al. Simultaneous detection and identification of STI pathogens by multiplex Real-Time PCR in genital tract specimens in a selected area of Apulia, a region of Southern Italy. Infection **2017**; 45:469–477.

34. Jenniskens MLM, Veerbeek JHW, Deurloo KL, van Hannen EJ, Thijsen SFT. Routine testing of Mycoplasma genitalium and Trichomonas vaginalis. Infect Dis (Lond) **2017**; 49:461–465.

35. Tabrizi SN, Su J, Bradshaw CS, et al. Prospective Evaluation of ResistancePlus MG, a New Multiplex Quantitative PCR Assay for Detection of Mycoplasma genitalium and Macrolide Resistance. J Clin Microbiol **2017**; 55:1915–1919.

36. Edouard S, Tamalet C, Tissot-Dupont H, et al. Evaluation of self-collected rectal swabs for the detection of bacteria responsible for sexually transmitted infections in a cohort of HIV-1-infected patients. J Med Microbiol **2017**; 66:693–697.

37. Gratrix J, Plitt S, Turnbull L, et al. Prevalence and antibiotic resistance of Mycoplasma genitalium among STI clinic attendees in Western Canada: a cross-sectional analysis. BMJ Open **2017**; 7:e016300.

38. Chernesky M, Jang D, Smieja M, et al. Urinary Meatal Swabbing Detects More Men Infected With Mycoplasma genitalium and Four Other Sexually Transmitted Infections Than First Catch Urine. Sex Transm Dis **2017**; 44:489–491.

39. Edouard S, Tissot-Dupont H, Dubourg G, et al. Mycoplasma genitalium, an agent of reemerging sexually transmitted infections. APMIS **2017**; 125:916–920.

40. Madsen AMR, Thorsteinsson K, Lebech A-M, et al. Prevalence and significance of Mycoplasma genitalium in women living with HIV in Denmark. BMC Res Notes **2017**; 10:468.

41. Hakre S, Casimier RO, Danboise BA, et al. Enhanced Sexually Transmitted Infection Screening for Mycoplasma genitalium in Human Immunodeficiency Virus -Infected US Air Force Personnel. Clin Infect Dis **2017**; 65:1585–1588.

42. Leli C, Mencacci A, Latino MA, et al. Prevalence of cervical colonization by Ureaplasma parvum, Ureaplasma urealyticum, Mycoplasma hominis and Mycoplasma genitalium in childbearing age women by a commercially available multiplex real-time PCR: An Italian observational multicentre study. J Microbiol Immunol Infect **2018**; 51:220–225.

43. Tjagur S, Mändar R, Punab M. Prevalence of Mycoplasma genitalium and other sexually transmitted infections causing urethritis among high-risk heterosexual male patients in Estonia. Infect Dis (Lond) **2018**; 50:133–139.

44. Unemo M, Salado-Rasmussen K, Hansen M, et al. Clinical and analytical evaluation of the new Aptima Mycoplasma genitalium assay, with data on M. genitalium prevalence and antimicrobial resistance in M. genitalium in Denmark, Norway and Sweden in 2016. Clin Microbiol Infect **2018**; 24:533–539.

45. Dionne-Odom J, Geisler WM, Aaron KJ, et al. High Prevalence of Multidrug-Resistant Mycoplasma genitalium in Human Immunodeficiency Virus-Infected Men Who Have Sex With Men in Alabama. Clin Infect Dis **2018**; 66:796–798.

46. Upton A, Bissessor L, Lowe P, Wang X, McAuliffe G. Diagnosis of Chlamydia trachomatis, Neisseria gonorrhoeae, Trichomonas vaginalis and Mycoplasma genitalium: an observational study of testing patterns, prevalence and co-infection rates in northern New Zealand. Sex Health **2018**; 15:232–237.

47. Balkus JE, Manhart LE, Jensen JS, et al. Mycoplasma genitalium Infection in Kenyan and US Women. Sex Transm Dis **2018**; 45:514–521.

48. Lockhart A, Psioda M, Ting J, et al. Prospective Evaluation of Cervicovaginal Self- and Cervical Physician Collection for the Detection of Chlamydia trachomatis, Neisseria gonorrhoeae, Trichomonas vaginalis, and Mycoplasma genitalium Infections. Sex Transm Dis **2018**; 45:488–493.

49. Allan-Blitz L-T, Mokany E, Campeau S, Wee R, Shannon C, Klausner JD. Prevalence of Mycoplasma genitalium and Azithromycin-resistant Infections Among Remnant Clinical Specimens, Los Angeles. Sex Transm Dis **2018**; 45:632–635.

50. Chra P, Papaparaskevas J, Papadogeorgaki E, et al. Prevalence of Mycoplasma genitalium and other sexually-transmitted pathogens among high-risk individuals in Greece. Germs **2018**; 8:12–20.

51. Couldwell DL, Jalocon D, Power M, Jeoffreys NJ, Chen SC-A, Lewis DA. Mycoplasma genitalium: high prevalence of resistance to macrolides and frequent anorectal infection in men who have sex with men in western Sydney. Sex Transm Infect **2018**; 94:406–410.

52. Trent M, Coleman JS, Hardick J, et al. Clinical and sexual risk correlates of Mycoplasma genitalium in urban pregnant and non-pregnant young women: cross-sectional outcomes using the baseline data from the Women’s BioHealth Study. Sex Transm Infect **2018**; 94:411–413.

53. Brosh-Nissimov T, Kedem R, Ophir N, Shental O, Keller N, Amit S. Management of sexually transmissible infections in the era of multiplexed molecular diagnostics: a primary care survey. Sex Health **2018**; 15:298–303.

54. Trevis T, Gossé M, Santarossa N, Tabrizi S, Russell D, McBride WJ. Mycoplasma genitalium in the Far North Queensland backpacker population: An observational study of prevalence and azithromycin resistance. PLoS One **2018**; 13:e0202428.

55. Kaida A, Dietrich JJ, Laher F, et al. A high burden of asymptomatic genital tract infections undermines the syndromic management approach among adolescents and young adults in South Africa: implications for HIV prevention efforts. BMC Infect Dis **2018**; 18:499.

56. Clifton S, Mercer CH, Sonnenberg P, et al. STI Risk Perception in the British Population and How It Relates to Sexual Behaviour and STI Healthcare Use: Findings From a Cross-sectional Survey (Natsal-3). EClinicalMedicine **2018**; 2–3:29–36.

57. Lillis RA, Martin DH, Nsuami MJ. Mycoplasma genitalium Infections in Women Attending a Sexually Transmitted Disease Clinic in New Orleans. Clin Infect Dis **2019**; 69:459–465.

58. Nolskog P, Backhaus E, Nasic S, Enroth H. STI with Mycoplasma genitalium-more common than Chlamydia trachomatis in patients attending youth clinics in Sweden. Eur J Clin Microbiol Infect Dis **2019**; 38:81–86.

59. Hoffman CM, Mbambazela N, Sithole P, et al. Provision of Sexually Transmitted Infection Services in a Mobile Clinic Reveals High Unmet Need in Remote Areas of South Africa: A Cross-sectional Study. Sex Transm Dis **2019**; 46:206–212.

60. Ferré VM, Gbeasor-Komlanvi FA, Collin G, et al. Prevalence of Human Papillomavirus, Human Immunodeficiency Virus, and Other Sexually Transmitted Infections Among Men Who Have Sex With Men in Togo: A National Cross-sectional Survey. Clin Infect Dis **2019**; 69:1019–1026.

61. Mulligan V, Lynagh Y, Clarke S, Unemo M, Crowley B. Prevalence, Macrolide Resistance, and Fluoroquinolone Resistance in Mycoplasma genitalium in Men Who Have Sex With Men Attending an Sexually Transmitted Disease Clinic in Dublin, Ireland in 2017-2018. Sex Transm Dis **2019**; 46:e35–e37.

62. Ong JJ, Magooa MP, Chikandiwa A, et al. Prevalence and Antimicrobial Resistance of Mycoplasma genitalium Infection Among Women Living With Human Immunodeficiency Virus in South Africa: A Prospective Cohort Study. Clin Infect Dis **2019**; 69:873–876.

63. Deborde M, Pereyre S, Puges M, et al. High prevalence of Mycoplasma genitalium infection and macrolide resistance in patients enrolled in HIV pre-exposure prophylaxis program. Med Mal Infect **2019**; 49:347–349.

64. Harrison MA, Harding-Esch EM, Marks M, et al. Impact of mass drug administration of azithromycin for trachoma elimination on prevalence and azithromycin resistance of genital Mycoplasma genitalium infection. Sex Transm Infect **2019**; 95:522–528.

65. Ferré VM, Ekouevi DK, Gbeasor-Komlanvi FA, et al. Prevalence of human papillomavirus, human immunodeficiency virus and other sexually transmitted infections among female sex workers in Togo: a national cross-sectional survey. Clin Microbiol Infect **2019**; 25:1560.e1-1560.e7.

66. Batteiger TA, Jordan SJ, Toh E, et al. Detection of Rectal Chlamydia trachomatis in Heterosexual Men Who Report Cunnilingus. Sex Transm Dis **2019**; 46:440–445.

67. Gaydos CA, Manhart LE, Taylor SN, et al. Molecular Testing for Mycoplasma genitalium in the United States: Results from the AMES Prospective Multicenter Clinical Study. J Clin Microbiol **2019**; 57:e01125-19.

68. de Salazar A, Espadafor B, Fuentes-López A, et al. Comparison between Aptima Assays (Hologic) and the Allplex STI Essential Assay (Seegene) for the diagnosis of Sexually transmitted infections. PLoS One **2019**; 14:e0222439.

69. Güralp O, Bostancı A, Özerkman Başaran E, Schild-Suhren M, Kaya B. Evaluation of the prevalence of sexually transmitted bacterial pathogens in Northern Cyprus by nucleic acid amplification tests, and investigation of the relationship between these pathogens and cervicitis. Turk J Obstet Gynecol **2019**; 16:242–248.

70. Stewart JD, Webb B, Francis M, Graham M, Korman TM. Should we routinely test for Mycoplasma genitalium when testing for other sexually transmitted infections? Med J Aust **2020**; 212:30–31.

71. Spiller OB, Rees CL, Morris DJ, Davies RL, Jones LC. Mycoplasma genitalium prevalence in Welsh sexual health patients: Low antimicrobial resistance markers and no association of symptoms to bacterial load. Microb Pathog **2020**; 139:103872.

72. Alicja E, Małgorzata R, Małgorzata A, et al. Prevalence of urogenital mycoplasmas in women with systemic lupus erythematosus (SLE): preliminary study. Eur J Clin Microbiol Infect Dis **2020**; 39:717–721.

73. Crowell TA, Lawlor J, Lombardi K, et al. Anorectal and Urogenital Mycoplasma genitalium in Nigerian Men Who Have Sex With Men and Transgender Women: Prevalence, Incidence, and Association With HIV. Sex Transm Dis **2020**; 47:202–206.

74. Smullin CP, Green H, Peters R, et al. Prevalence and incidence of Mycoplasma genitalium in a cohort of HIV-infected and HIV-uninfected pregnant women in Cape Town, South Africa. Sex Transm Infect **2020**; 96:501–508.

75. Spornraft-Ragaller P, Dumke R. Prevalence and antibiotic resistance of rectal Mollicutes in HIV-infected men who have sex with men at the University Hospital of Dresden, Germany. Infection **2020**; 48:259–265.

76. Jansen K, Steffen G, Potthoff A, et al. STI in times of PrEP: high prevalence of chlamydia, gonorrhea, and mycoplasma at different anatomic sites in men who have sex with men in Germany. BMC Infect Dis **2020**; 20:110.

77. Bernier A, Rumyantseva T, Reques L, et al. HIV and other sexually transmitted infections among female sex workers in Moscow (Russia): prevalence and associated risk factors. Sex Transm Infect **2020**; 96:601–607.

78. Khosropour CM, Jensen JS, Soge OO, et al. High Prevalence of Vaginal and Rectal Mycoplasma genitalium Macrolide Resistance Among Female Sexually Transmitted Disease Clinic Patients in Seattle, Washington. Sex Transm Dis **2020**; 47:321–325.

79. Manhart LE, Gaydos CA, Taylor SN, et al. Characteristics of Mycoplasma genitalium Urogenital Infections in a Diverse Patient Sample from the United States: Results from the Aptima Mycoplasma genitalium Evaluation Study (AMES). J Clin Microbiol **2020**; 58:e00165-20.

80. Park JH, Kim N, Shin S, Roh EY, Yoon JH, Park H. Prevalence and correlated factors of sexually transmitted infections among women attending a Korean sexual assault center. J Forensic Leg Med **2020**; 71:101935.

81. Cowley G, Milne G, Teixeira da Silva E, et al. Prevalence of and risk factors for curable sexually transmitted infections on Bubaque Island, Guinea Bissau. Sex Transm Infect **2021**; 97:51–55.

82. Grad AI, Vica ML, Ungureanu L, Siserman CV, Tătaru AD, Matei HV. Assessment of STI screening in Romania using a multiplex PCR technique. J Infect Dev Ctries **2020**; 14:341–348.

83. Hanna J, Yassine R, El-Bikai R, et al. Molecular epidemiology and socio-demographic risk factors of sexually transmitted infections among women in Lebanon. BMC Infect Dis **2020**; 20:375.

84. Kharsany ABM, McKinnon LR, Lewis L, et al. Population prevalence of sexually transmitted infections in a high HIV burden district in KwaZulu-Natal, South Africa: Implications for HIV epidemic control. Int J Infect Dis **2020**; 98:130–137.

85. Hilmarsdóttir I, Arnardóttir EM, Jóhannesdóttir ER, et al. Prevalence of Mycoplasma genitalium and Antibiotic Resistance-Associated Mutations in Patients at a Sexually Transmitted Infection Clinic in Iceland, and Comparison of the S-DiaMGTV and Aptima Mycoplasma genitalium Assays for Diagnosis. J Clin Microbiol **2020**; 58:e01084-20.

86. Juliana NCA, Deb S, Ouburg S, et al. The Prevalence of Chlamydia trachomatis and Three Other Non-Viral Sexually Transmitted Infections among Pregnant Women in Pemba Island Tanzania. Pathogens **2020**; 9:625.

87. Ng KKM, Leung PKL, Cheung TKM. Molecular detection of Mycoplasma genitalium in endocervical swabs and associated rates of macrolide and fluoroquinolone resistance in Hong Kong. Hong Kong Med J **2020**; 26:390–396.

88. Munson E, Reynoso A, Pass M, et al. Comprehensive Molecular Screening in a Cohort of Young Men Who Have Sex With Men and Transgender Women: Effect of Additive Rectal Specimen Source Collection and Analyte Testing. Sex Transm Dis **2020**; 47:748–753.

89. Angela A, Raffaele DP, Federica R, Adriana M, Luigi S, Luigi R. Multi-year prevalence and macrolide resistance of Mycoplasma genitalium in clinical samples from a southern Italian hospital. Eur J Clin Microbiol Infect Dis **2021**; 40:893–895.

90. Karim S, Bouchikhi C, Banani A, et al. Bacterial sexually transmitted infections and syndromic approach: a study conducted on women at Moroccan University Hospital. Germs **2021**; 11:544–553.

91. Balle C, Gill K, Konstantinus IN, et al. Hormonal contraception and risk of STIs and bacterial vaginosis in South African adolescents: secondary analysis of a randomised trial. Sex Transm Infect **2021**; 97:112–117.

92. Nijhuis RHT, Duinsbergen RG, Pol A, Godschalk PCR. Prevalence of Chlamydia trachomatis, Neisseria gonorrhoeae, Mycoplasma genitalium and Trichomonas vaginalis including relevant resistance-associated mutations in a single center in the Netherlands. Eur J Clin Microbiol Infect Dis **2021**; 40:591–595.

93. Oh EJ, Jang TS, Kim JK. Mycoplasma genitalium and Mycoplasma hominis infection in south Korea during 2018-2020. Iran J Microbiol **2021**; 13:602–607.

94. Perin J, Coleman JS, Ronda J, Neibaur E, Gaydos CA, Trent M. Maternal and Fetal Outcomes in an Observational Cohort of Women With Mycoplasma genitalium Infections. Sex Transm Dis **2021**; 48:991–996.

95. Desdorf R, Andersen NM, Chen M. Mycoplasma genitalium prevalence and macrolide resistance-associated mutations and coinfection with Chlamydia trachomatis in Southern Jutland, Denmark. APMIS **2021**; 129:706–710.

96. Naicker M, Singh R, van der Westhuizen D, Tinarwo P, Abbai NS. Lack of resistance to macrolides in Mycoplasma genitalium detected in South African pregnant women. S Afr J Infect Dis **2021**; 36:209.

97. Rowlinson E, Hughes JP, Chambers LC, et al. Incidence of Nongonococcal Urethritis in Men Who Have Sex With Women and Associated Risk Factors. Sex Transm Dis **2021**; 48:341–346.

98. Parmar NR, Mushanski L, Wanlin T, et al. High Prevalence of Macrolide and Fluoroquinolone Resistance-Mediating Mutations in Mycoplasma genitalium-Positive Urine Specimens From Saskatchewan. Sex Transm Dis **2021**; 48:680–684.

99. Calas A, Zemali N, Camuset G, et al. Prevalence of urogenital, anal, and pharyngeal infections with Chlamydia trachomatis, Neisseria gonorrhoeae, and Mycoplasma genitalium: a cross-sectional study in Reunion island. BMC Infect Dis **2021**; 21:95.

100. Lucena Nemirosky J, Espelt R, López Grado E, et al. Macrolide resistance in Mycoplasma genitalium in Catalonia, Spain: a 1 year prospective study. J Antimicrob Chemother **2021**; 76:2702–2707.

101. Han Y, Yin Y-P, Liu J-W, et al. Rectal Mycoplasma genitalium in Patients Attending Sexually Transmitted Disease Clinics in China: An Infection That Cannot Be Ignored. Infect Drug Resist **2021**; 14:2509–2515.

102. Torrone EA, Kruszon-Moran D, Philips C, et al. Prevalence of Urogenital Mycoplasma genitalium Infection, United States, 2017 to 2018. Sex Transm Dis **2021**; 48:e160–e162.

103. Zhang X-H, Zhao P-Z, Ke W-J, et al. Prevalence and correlates of Mycoplasma genitalium infection among patients attending a sexually transmitted infection clinic in Guangdong, China: a cross-sectional study. BMC Infect Dis **2021**; 21:649.

104. Scoullar MJL, Boeuf P, Peach E, et al. Mycoplasma genitalium and Other Reproductive Tract Infections in Pregnant Women, Papua New Guinea, 2015-2017. Emerg Infect Dis **2021**; 27:894–904.

105. Taku O, Brink A, Meiring TL, et al. Detection of sexually transmitted pathogens and co-infection with human papillomavirus in women residing in rural Eastern Cape, South Africa. PeerJ **2021**; 9:e10793.

106. Jary A, Teguete I, Sidibé Y, et al. Prevalence of cervical HPV infection, sexually transmitted infections and associated antimicrobial resistance in women attending cervical cancer screening in Mali. Int J Infect Dis **2021**; 108:610–616.

107. Llangarí-Arizo LM, Sadiq ST, Márquez C, et al. Sexually transmitted infections and factors associated with risky sexual practices among female sex workers: A cross sectional study in a large Andean city. PLoS One **2021**; 16:e0250117.

108. Hammer A, Gravitt PE, Adcock R, et al. Burden of Mycoplasma genitalium and Bacterial Coinfections in a Population-Based Sample in New Mexico. Sex Transm Dis **2021**; 48:e186–e189.

109. Begnis R, Bouscaren N, Raffray L, et al. Prevalence and risk factors of Mycoplasma genitalium infection in patients attending a sexually transmitted infection clinic in Reunion Island: a cross-sectional study (2017-2018). BMC Infect Dis **2021**; 21:482.

110. Stafford IA, Hummel K, Dunn JJ, et al. Retrospective analysis of infection and antimicrobial resistance patterns of Mycoplasma genitalium among pregnant women in the southwestern USA. BMJ Open **2021**; 11:e050475.

111. Horseman TS, Crecelius EM, Miller MA, et al. Prevalence and Epidemiology of Mycoplasma genitalium in a Pacific-Region Military Population. Sex Transm Dis **2021**; 48:578–582.

112. Kenyon C, De Baetselier I, Vanbaelen T, Buyze J, Florence E. The Population-Level Effect of Screening for Mycoplasma genitalium on Antimicrobial Resistance: A Quasi-Experimental Study. Sex Transm Dis **2021**; 48:629–634.

113. Beayni NE, Hamad L, Nakad C, Keleshian S, Yazbek SN, Mahfouz R. Molecular prevalence of eight different sexually transmitted infections in a Lebanese major tertiary care center: impact on public health. Int J Mol Epidemiol Genet **2021**; 12:16–23.

114. Tovo SF, Zohoncon TM, Dabiré AM, et al. Molecular Epidemiology of Human Papillomaviruses, Neisseria gonorrhoeae, Chlamydia trachomatis and Mycoplasma genitalium among Female Sex Workers in Burkina Faso: Prevalence, Coinfections and Drug Resistance Genes. Trop Med Infect Dis **2021**; 6:90.

115. Martín-Saco G, Tristancho A, Arias A, Ferrer I, Milagro A, García-Lechuz JM. Mycoplasma genitalium and sexually transmitted infections: evidences and figures in a tertiary hospital. Rev Esp Quimioter **2022**; 35:76–79.

116. Brin C, Palich R, Godefroy N, et al. Clinical, epidemiological and therapeutic characteristics of Mycoplasma genitalium infection in a French STI center. Infect Dis Now **2022**; 52:13–17.

117. Shilling HS, Garland SM, Costa A-M, et al. Chlamydia trachomatis and Mycoplasma genitalium prevalence and associated factors among women presenting to a pregnancy termination and contraception clinic, 2009-2019. Sex Transm Infect **2022**; 98:115–120.

118. Latimer RL, Vodstrcil LA, Plummer EL, et al. The clinical indications for testing women for Mycoplasma genitalium. Sex Transm Infect **2022**; 98:277–285.

119. Wang R, Trent ME, Bream JH, et al. Mycoplasma genitalium Infection Is Not Associated With Genital Tract Inflammation Among Adolescent and Young Adult Women in Baltimore, Maryland. Sex Transm Dis **2022**; 49:139–144.

120. Tu W, Li Y-Y, Kuang Y-Q, et al. High prevalence of sexually transmitted infections and risk factors among HIV-positive individuals in Yunnan, China. Eur J Med Res **2022**; 27:9.

121. Liu T, Lai S-Y, Zhou W, Liu Y-L, Chen S-S, Jiang Y-M. Analysis of Ureaplasma urealyticum, Chlamydia trachomatis, Mycoplasma genitalium and Neisseria gonorrhoeae infections among obstetrics and gynecological outpatients in southwest China: a retrospective study. BMC Infect Dis **2022**; 22:283.

122. Klavs I, Milavec M, Berlot L, et al. Prevalence of sexually transmitted infections with Chlamydia trachomatis, Neisseria gonorrhoeae, Mycoplasma genitalium and Trichomonas vaginalis: findings from the National Survey of Sexual Lifestyles, Attitudes and Health, Slovenia, 2016 to 2017. Euro Surveill **2022**; 27:2100284.

123. Streeck H, Jansen K, Crowell TA, et al. HIV pre-exposure prophylaxis was associated with no impact on sexually transmitted infection prevalence in a high-prevalence population of predominantly men who have sex with men, Germany, 2018 to 2019. Euro Surveill **2022**; 27:2100591.

124. Herms F, Poizeau F, Anyfantakis V, et al. Mycoplasma genitalium screening in a specialized French unit: A retrospective study. Ann Dermatol Venereol **2022**; 149:165–168.

125. Che G, Liu F, Yang Q, et al. Mycoplasma genitalium and Chlamydia trachomatis infection among women in Southwest China: a retrospective study. Epidemiol Infect **2022**; 150:e129.

126. Ring A, Balakrishna S, Imkamp F, et al. High Rates of Asymptomatic Mycoplasma genitalium Infections With High Proportion of Genotypic Resistance to First-Line Macrolide Treatment Among Men Who Have Sex With Men Enrolled in the Zurich Primary HIV Infection Study. Open Forum Infect Dis **2022**; 9:ofac217.

127. Scaglione E, Mantova G, Caturano V, et al. Molecular Epidemiology of Genital Infections in Campania Region: A Retrospective Study. Diagnostics (Basel) **2022**; 12:1798.

128. Asare K, Osman F, Ngcapu S, et al. Burden of sexually transmitted infections from acute HIV infection among women in South Africa: Evidence from a prospective cohort study. Ann Epidemiol **2022**; 74:132–139.

129. Skjælaaen K, Nesvold H, Brekke M, et al. Sexually transmitted infections among patients attending a sexual assault centre: a cohort study from Oslo, Norway. BMJ Open **2022**; 12:e064934.

130. De Baetselier I, Vuylsteke B, Cuylaerts V, et al. Mycoplasma genitalium and Antimicrobial Resistance Among a Cohort of West African Men Who Have Sex With Men Using Preexposure Prophylaxis (CohMSM-PrEP ANRS 12369-Expertise France Study). Open Forum Infect Dis **2022**; 9:ofac615.

131. Mwaniki SW, Kaberia PM, Mugo PM, Palanee-Phillips T. Prevalence of five curable sexually transmitted infections and associated risk factors among tertiary student men who have sex with men in Nairobi, Kenya: a respondent-driven sampling survey†. Sex Health **2023**; 20:105–117.

132. Sienkiewicz L, Thomas Y, Reynoso A, Munson E. Incidence and laboratory diagnosis of sexually-transmitted infections among university students in a high-prevalence community. J Am Coll Health **2023**; 71:571–577.

133. Hu M, Souder JP, Subramaniam A, et al. Prevalence of Mycoplasma genitalium infection and macrolide resistance in pregnant women receiving prenatal care. Int J Gynaecol Obstet **2023**; 160:341–344.

134. Perry MD, Jones S, Bertram A, et al. The prevalence of Mycoplasma genitalium (MG) and Trichomonas vaginalis (TV) at testing centers in Belgium, Germany, Spain, and the UK using the cobas TV/MG molecular assay. Eur J Clin Microbiol Infect Dis **2023**; 42:43–52.

135. Zhang Z, Zong X, Bai H, Fan L, Li T, Liu Z. Prevalence of Mycoplasma genitalium and Chlamydia trachomatis in Chinese female with lower reproductive tract infection: a multicenter epidemiological survey. BMC Infect Dis **2023**; 23:2.

136. Maldonado-Barrueco A, Rodríguez-Ayala M, Grandioso-Vas D, et al. Epidemiology and prevalence of mutations associated with resistance to macrolides and fluoroquinolones in Mycoplasma genitalium in a tertiary hospital from Madrid, Spain. Rev Esp Quimioter **2023**; 36:310–313.

137. Shipitsyna E, Kularatne R, Golparian D, et al. Mycoplasma genitalium prevalence, antimicrobial resistance-associated mutations, and coinfections with non-viral sexually transmitted infections in high-risk populations in Guatemala, Malta, Morocco, Peru and South Africa, 2019-2021. Front Microbiol **2023**; 14:1130762.

138. Sandri A, Carelli M, Visentin A, et al. Mycoplasma genitalium antibiotic resistance-associated mutations in genital and extragenital samples from men-who-have-sex-with-men attending a STI clinic in Verona, Italy. Front Cell Infect Microbiol **2023**; 13:1155451.

139. de Jesus Salgado V, de Abreu Oliveira CMP, da Silva ÁMB, et al. Prevalence of Mollicutes among men who have sex with men and transgender women aged 15 to 19 years in Salvador, North-eastern Brazil. BMC Infect Dis **2023**; 23:244.

140. Yusuf E, Mertens K, van Lisdonk N, Houwen C, Thai KTD. Epidemiology of Mycoplasma genitalium and Trichomonas vaginalis in the primary health care setting in the Netherlands. Epidemiol Infect **2023**; 151:e79.

141. Govender V, Moodley D, Naidoo M, Connoly C, Ngcapu S, Abdool Karim Q. High incidence of asymptomatic genital tract infections in pregnancy in adolescent girls and young women: need for repeat aetiological screening. Sex Transm Infect **2023**; 99:482–488.

142. Menezes ME, Silver EJ, Goldstein DY, Collins-Ogle MD, Fox AS, Coupey SM. Prevalence and Factors Associated With Mycoplasma genitalium Infection in At-Risk Female Adolescents in Bronx County, New York. Sex Transm Dis **2023**; 50:635–641.

143. Manhart LE, Leipertz G, Soge OO, et al. Mycoplasma genitalium in the US (MyGeniUS): Surveillance Data From Sexual Health Clinics in 4 US Regions. Clin Infect Dis **2023**; 77:1449–1459.

144. Totten AH, Xiao L, Van Der Pol B, Szychowski J, Subramaniam A, Geisler WM. Retrospective Evaluation of Mycoplasma genitalium Prevalence and Macrolide Resistance in a Study Cohort of Pregnant Women in Birmingham, AL, From 1997 to 2001. Sex Transm Dis **2023**; 50:642–644.

145. Butcher R, Jarju S, Obayemi D, et al. Prevalence of five treatable sexually transmitted infections among women in Lower River region of The Gambia. BMC Infect Dis **2023**; 23:471.

146. Lokken EM, Kabare E, Oyaro B, et al. A prospective preconception cohort study of the association between Mycoplasma genitalium and fecundability in Kenyan women trying to conceive. Hum Reprod **2023**; 38:2020–2027.

147. Amesty S, Perez-Figueroa R, Stonbraker S, et al. High burden of sexually transmitted infections among under-resourced populations in the Dominican Republic. Ther Adv Infect Dis **2023**; 10:20499361231193561.

148. Harryparsad R, Meyer B, Taku O, et al. Prevalence and incidence of sexually transmitted infections among South African women initiating injectable and long-acting contraceptives. PLoS One **2023**; 18:e0294285.

149. Valasoulis G, Pouliakis A, Michail G, et al. Cervical HPV Infections, Sexually Transmitted Bacterial Pathogens and Cytology Findings-A Molecular Epidemiology Study. Pathogens **2023**; 12:1347.

150. Filho AC, Marcos CRSA, Colnago JM, Miranda AEB, Duarte JN, Peruchi LS. Sexually transmitted infections with Chlamydia trachomatis, Neisseria gonorrhoeae, Mycoplasma genitalium, and Trichomonas vaginalis in pregnant women as detected by molecular testing. Indian J Sex Transm Dis AIDS **2023**; 44:139–142.

151. Lê A-S, Labbé A-C, Fourmigue A, et al. Mycoplasma genitalium infection among gay, bisexual and other men who have sex with men in Montréal, Canada. Can Commun Dis Rep **2023**; 49:477–486.

152. Lindman J, Djalo MA, Biai A, et al. Prevalence of sexually transmitted infections and associated risk factors among female sex workers in Guinea-Bissau. Sex Transm Infect **2024**; 100:411–417.

153. Romo ML, Moreland SC, Yates AM, et al. Prevalence of Urogenital Mycoplasma genitalium Infection at 2 US Army Medical Facilities. Sex Transm Dis **2024**; 51:367–373.

154. Beesham I, Isehunwa O, Kriel Y, et al. Sexually Transmitted Infection Prevalence, Partner Notification, and Human Immunodeficiency Virus Risk Perception in a Cohort of Women Completing Sexually Transmitted Infection Screening as Part of a Safer Conception Study. Sex Transm Dis **2024**; 51:431–436.

155. Miranda AE, Gaspar PC, Schörner MA, et al. Prevalence of Chlamydia trachomatis, Neisseria gonorrhoeae, Trichomonas vaginalis, and Mycoplasma genitalium and risk factors among pregnant women in Brazil: Results from the national molecular diagnosis implementation project. Int J Gynaecol Obstet **2024**; 166:71–79.

156. Lavey SC, Cassel B, Munson E. Mycoplasmoides genitalium Macrolide Resistance Detection is Needed in University Settings. Clin Med Res **2024**; 22:13–18.

157. Ljubin-Sternak S, Meštrović T, Marijan T, Anušić M, Šuto S, Vraneš J. Detection of Macrolide and/or Fluoroquinolone Resistance Genes in Mycoplasma genitalium Strains Isolated from Men in the Northwest Region of Croatia in 2018-2023. Genes (Basel) **2024**; 15:470.

158. Tsai M-J, Sun H-Y, Su L-H, et al. Mycoplasma genitalium infection and resistance-associated mutations to macrolides and fluoroquinolones among high-risk patients in Taiwan. J Microbiol Immunol Infect **2024**; 57:629–637.

159. Lam PPH, Nguyen NH, Nguyen TTT, Trinh NB, Luong BA. Mycoplasma genitalium prevalence, co-infection and macrolide resistance-associated mutations in Southern Vietnam. Infez Med **2024**; 32:222–230.

160. Scoullar MJL, Melepia P, Peach E, et al. Mycoplasma genitalium in pregnancy, including specific co-infections, is associated with lower birthweight: A prospective cohort study. Med **2024**; 5:1123-1136.e3.

161. Kadylak D, Czarny J, Nowicki RJ, Sokołowska-Wojdyło M. The Prevalence of Sexually Transmitted Infections among Male Patients at a Dermato-Venereology Outpatient Clinic in Gdańsk, Poland: Findings from a Single-Center Study. J Clin Med **2024**; 13:3736.

162. Nguyen KD, Adamson PC, Bui HT, et al. Mycoplasma genitalium Infections Among Participants in an HIV Pre-exposure Prophylaxis Program in Hanoi, Vietnam. Sex Transm Dis **2024**; 51:750–755.

163. Kostera J, Tursunovic A, Botts P, Galloway R, Davis A, Yang T. Prevalence of co-infection between high-risk human papillomavirus and common sexually transmitted infections in cervical specimens. Sex Transm Infect **2024**; 100:470–471.

164. Hakre S, Sanders-Buell E, Casimier RO, et al. Prevalence of Mycoplasma genitalium Infection and Macrolide and Fluoroquinolone Resistance Mutations Among US Air Force Service Members With HIV, 2016-2020. Open Forum Infect Dis **2024**; 11:ofae407.

165. Bjartling C, Kertes R, Kristiansen S, Johnsson A, Forslund O. Prevalence of Mycoplasma genitalium and macrolide resistance in rectal and urine samples among men who have sex with men in Sweden. Sex Transm Infect **2024**; 100:430–434.

166. Zonta MA, Liljander A, Roque KB, et al. Prevalence of sexually transmitted infections and human papillomavirus in cervical samples from incarcerated women in São Paulo, Brazil: a retrospective single-center study. Front Public Health **2024**; 12:1353845.

167. Llangarí-Arizo LM, Broad CE, Zhou L, et al. Sexually transmitted infections among at-risk women in Ecuador: implications for global prevalence and testing practices for STIs detected only at the anorectum in female sex workers. Sex Transm Infect **2024**; 100:504–511.

168. Wang F, Zhang C, Xiu L, et al. Etiological, sociodemographic and clinical characteristics of sexually transmitted infections and M. genitalium resistance in Shenzhen: a multicenter cross-sectional study in China. Front Cell Infect Microbiol **2024**; 14:1407124.

169. Sethi S, Boris GDE, Sharma N, Kanaujia R, Roger KJ, de Dieu TJ. Prevalence and associated risk factors of Mycoplasma genitalium infection in women in Western Cameroon: A cross sectional study. Indian J Dermatol Venereol Leprol **2024**; :1–6.

170. Proudmore KE, Gunathilake M, Crawford LC, Freeman K, Menouhos D, Baird RW. Mycoplasma genitalium retrospective audit of Northern Territory isolates from 2022. Commun Dis Intell (2018) **2024**; 48.

171. Rizzo A, Moschese D, Salari F, et al. Anal HPV prevalence in individuals with and without other concomitant sexually transmitted infections. J Med Virol **2024**; 96:e29852.

172. Baiers RA, Ryan DT, Clifford A, et al. Asymptomatic Rectal Bacterial Pathogens Show Large Prospective Relationships With HIV Incidence in a Cohort of Young Sexual and Gender Minorities: Implications for STI Screening and HIV Prevention. Open Forum Infect Dis **2024**; 11:ofae444.

173. Tian J, Chen S, Li X, Teng Y, Chen B. Prevalence of sexually transmitted infections (STIs) among first time visitors at STIs clinic in Hangzhou, China: Assessing the influence of the COVID-19 pandemic. Immun Inflamm Dis **2024**; 12:e70009.

174. Sheele JM, Bragg K. Features Associated With Mycoplasma genitalium Infection. Cureus **2024**; 16:e72728.

175. Oliveira JM, Martins AH, Veiga D, Lavaredas C, Queirós A, Matos AM. Screening for STIs: Results of a Health-Promotion Programme in a Portuguese University. Microorganisms **2024**; 12:2479.

176. Schröder D, Sorano S, Shipitsyna E, et al. Prevalence and epidemiology of Mycoplasma genitalium and the absence of macrolide resistance in M. genitalium among pregnant women attending antenatal care in Zambia. Front Public Health **2025**; 13:1576376.

177. Kogler A, Sadoghi B, Draschl A, et al. Prevalence of Chlamydia trachomatis, Neisseria gonorrhoeae and Mycoplasma genitalium at pharyngeal and anorectal sites in patients presenting to an STI outpatient ward. J Eur Acad Dermatol Venereol **2025**; 39:389–397.

178. Leng X, Zhu R, Ao X, et al. Prevalence of Site-Specific Mycoplasma genitalium Infection and Macrolide and Fluoroquinolone-Associated Mutations in Men Who Have Sex with Men in Shenzhen, China. Infect Drug Resist **2025**; 18:239–252.

179. Nguyen HB, Nguyen HDK, Pham MQ, Nguyen CT, Adamson PC. Clinical characteristics and symptoms associated with Mycoplasma genitalium infections among heterosexual men in Hanoi, Vietnam. Sex Transm Infect **2025**; 101:361–366.

180. Kerschberger B, Lekelem S, Daka M, et al. Mycoplasma genitalium infection in Eswatini amid syndromic case management: prevalence, coinfections, diagnostic challenges and treatment gaps. BMC Infect Dis **2025**; 25:547.

181. Santana FAF, Bomfim J, Ferraz M, et al. Prevalence of Mollicutes in pregnant women undergoing high-risk prenatal care at a maternal and child reference unit in Bahia, Brazil. Epidemiol Infect **2025**; 153:e73.

182. Ito S, Hanaoka N, Shimuta K, et al. Male non-gonococcal urethritis: From microbiological etiologies to demographic and clinical features. Int J Urol **2016**; 23:325–331.

183. Saigal K, Dhawan B, Rawre J, Khanna N, Chaudhry R. Genital Mycoplasma and Chlamydia trachomatis infections in patients with genital tract infections attending a tertiary care hospital of North India. Indian J Pathol Microbiol **2016**; 59:194–196.

184. Dumke R, Thürmer A, Jacobs E. Emergence of Mycoplasma genitalium strains showing mutations associated with macrolide and fluoroquinolone resistance in the region Dresden, Germany. Diagn Microbiol Infect Dis **2016**; 86:221–223.

185. Moi H, Hartgill U, Skullerud KH, Reponen EJ, Syvertsen L, Moghaddam A. Microscopy of Stained Urethral Smear in Male Urethritis; Which Cutoff Should be Used? Sex Transm Dis **2017**; 44:189–194.

186. Le PT, Hamasuna R, Matsumoto M, et al. The detection of microorganisms related to urethritis from the oral cavity of male patients with urethritis. J Infect Chemother **2017**; 23:668–673.

187. Rietmeijer CA, Mungati M, Machiha A, et al. The Etiology of Male Urethral Discharge in Zimbabwe: Results from the Zimbabwe STI Etiology Study. Sex Transm Dis **2018**; 45:56–60.

188. Kufa T, Gumede L, Maseko DV, Radebe F, Kularatne R. The demographic and clinical profiles of women presenting with vaginal discharge syndrome at primary care facilities in South Africa: Associations with age and implications for management. S Afr Med J **2018**; 108:876–880.

189. Mahlangu MP, Müller EE, Venter JME, Maseko DV, Kularatne RS. The Prevalence of Mycoplasma genitalium and Association With Human Immunodeficiency Virus Infection in Symptomatic Patients, Johannesburg, South Africa, 2007-2014. Sex Transm Dis **2019**; 46:395–399.

190. McIver R, Jalocon D, McNulty A, et al. Men Who Have Sex With Men With Mycoplasma genitalium-Positive Nongonococcal Urethritis Are More Likely to Have Macrolide-Resistant Strains Than Men With Only Female Partners: A Prospective Study. Sex Transm Dis **2019**; 46:513–517.

191. Kato Y, Kawaguchi S, Shigehara K, et al. Prevalence of N. gonorrhoeae, C. trachomatis, M. genitalium, M. hominis and Ureaplasma spp. in the anus and urine among Japanese HIV-infected men who have sex with men. J Infect Chemother **2020**; 26:403–406.

192. Dhawan B, Rawre J, Dhawan N, Bhatia R, Gupta V, Khanna N. High prevalence of Mycoplasma genitalium in men who have sex with men: A cross-sectional study. Indian J Dermatol Venereol Leprol **2020**; 86:195–196.

193. Bachmann LH, Kirkcaldy RD, Geisler WM, et al. Prevalence of Mycoplasma genitalium Infection, Antimicrobial Resistance Mutations, and Symptom Resolution Following Treatment of Urethritis. Clin Infect Dis **2020**; 71:e624–e632.

194. Broad CE, Furegato M, Harrison MA, et al. High prevalence of coinfection of azithromycin-resistant Mycoplasma genitalium with other STIs: a prospective observational study of London-based symptomatic and STI-contact clinic attendees. Sex Transm Infect **2021**; 97:63–68.

195. Vives A, Cosentino M, Bassas L, Alonso C, Millan F. Epidemiological, clinical and laboratory differences between male urethral infections due to Haemophilus spp. and those due to Neisseria gonorrhoeae, Chlamydia trachomatis, Mycoplasma genitalium and Ureaplasma urealyticum: A descriptive study. Arch Ital Urol Androl **2021**; 93:468–474.

196. Xie J, Li X, Lü Y, et al. Female chronic posterior urethritis is underestimated in patients with lower urinary tract symptoms. Transl Androl Urol **2021**; 10:3456–3464.

197. Trent M, Perin J, Rowell J, et al. Using Innovation to Address Adolescent and Young Adult Health Disparities in Pelvic Inflammatory Disease: Design of the Technology Enhanced Community Health Precision Nursing (TECH-PN) Trial. J Infect Dis **2021**; 224:S145–S151.

198. Brehony C, Eogan M, Lambert JS, Drew RJ. Evaluation of molecular testing for Mycoplasma genitalium for symptomatic women. Ir J Med Sci **2022**; 191:1771–1775.

199. Braam JF, van Dam AP, Bruisten SM, et al. Macrolide-Resistant Mycoplasma genitalium Impairs Clinical Improvement of Male Urethritis After Empirical Treatment. Sex Transm Dis **2022**; 49:360–367.

200. Lee SS, Cheng KF, Wong NS, et al. Emergence of antibiotic-resistant Mycoplasma genitalium as the cause of non-gonococcal urethritis in male patients at a sexually transmitted infection clinic. Int J Antimicrob Agents **2022**; 59:106510.

201. Melendez JH, Hardick J, Onzia A, et al. Retrospective Analysis of Ugandan Men with Urethritis Reveals Mycoplasma genitalium and Associated Macrolide Resistance. Microbiol Spectr **2022**; 10:e0230421.

202. Nguyen BH, Pham QM, Hoang L, Sansone A, Jannini EA, Tran CM. Investigating the microbial pathogens of sexually transmitted infections among heterosexual Vietnamese men with symptomatic urethritis. Aging Male **2022**; 25:125–133.

203. Hamill MM, Onzia A, Wang T-H, et al. High burden of untreated syphilis, drug resistant Neisseria gonorrhoeae, and other sexually transmitted infections in men with urethral discharge syndrome in Kampala, Uganda. BMC Infect Dis **2022**; 22:440.

204. Kazemian H, Zarandi MK, Zargoush Z, et al. The prevalence of gonococcal and non-gonococcal infections in women referred to obstetrics and gynecology clinics. Infez Med **2022**; 30:247–253.

205. Kirkoyun Uysal H, Koksal MO, Sarsar K, et al. Prevalence of Chlamydia trachomatis, Neisseria gonorrhoeae, and Mycoplasma genitalium among Patients with Urogenital Symptoms in Istanbul. Healthcare (Basel) **2023**; 11:930.

206. Msemwa B, Mushi MF, Kidenya B, et al. Urogenital pathogens in urine samples of clinically diagnosed urinary tract infected patients in Tanzania: A laboratory based cross-sectional study. IJID Reg **2023**; 7:170–175.

207. Berti V, Blondel J, Spindler L, et al. Infective anoproctitis in men having sex with men: Don’t forget Mycoplasma genitalium. Infect Dis Now **2023**; 53:104771.

208. Omosa-Manyonyi GS, de Kam M, Tostmann A, et al. Evaluation and optimization of the syndromic management of female genital tract infections in Nairobi, Kenya. BMC Infect Dis **2023**; 23:547.

209. Llaca-Díaz J, Medina-Loredo V, Huerta-López D, Casillas-Vega N. Sexually Transmitted Infections in Male Patients with Urethritis. Pathogens **2023**; 12:1434.

210. Bazzo ML, Machado H de M, Martins JM, et al. Aetiological molecular identification of sexually transmitted infections that cause urethral discharge syndrome and genital ulcer disease in Brazilian men: a nationwide study. Sex Transm Infect **2024**; 100:133–137.

211. Dunaiski CM, Kock MM, Jung H, Peters RPH. Prospective Cohort Study of Treatment Outcomes of Vaginal Discharge Syndrome in Women in Windhoek, Namibia. Sex Transm Dis **2024**; 51:460–465.

212. Manjate A, Sergon G, Kenga D, et al. Prevalence of sexually transmitted infections (STIs), associations with sociodemographic and behavioural factors, and assessment of the syndromic management of vaginal discharge in women with urogenital complaints in Mozambique. Front Reprod Health **2024**; 6:1323926.

213. Sukhija-Cohen AC, Patani H, Robinson AC, Santos MR, Granados Y. Mycoplasma genitalium Incidence, Coinfection, and Antibiotic Resistance: A Prospective Study at a Walk-In Clinic in Los Angeles County, CA. Open Forum Infect Dis **2024**; 11:ofae419.

214. Schwebke JR, Nyirjesy P, Dsouza M, Getman D. Vaginitis and risk of sexually transmitted infections: results of a multi-center U.S. clinical study using STI nucleic acid amplification testing. J Clin Microbiol **2024**; 62:e0081624.

215. Vodstrcil LA, Plummer EL, Nguyen TV, et al. Trends in infections detected in women with cervicitis over a decade. Front Reprod Health **2025**; 7:1539186.

216. Kanu EM, Rottmann H, Olaru ID, et al. Sexually transmitted infections in women in a rural hospital in Sierra Leone: a retrospective database study. IJID Reg **2025**; 15:100652.

217. Tandon D, Ramachandra S, Kerkar SC, et al. Evaluation of aetiological profiles, treatment appropriateness, and outcomes of syndromic management for reproductive tract infections in women from urban clinics. Indian J Dermatol Venereol Leprol **2025**; :1–7.

218. Kim SI, Yoon JH, Park DC, et al. Co-infection Of Ureaplasma urealyticum And Human Papilloma Virus In Asymptomatic Sexually Active Individuals. Int J Med Sci **2018**; 15:915–920.

219. Lima L de M, Hoelzle CR, Simões RT, et al. Sexually Transmitted Infections Detected by Multiplex Real Time PCR in Asymptomatic Women and Association with Cervical Intraepithelial Neoplasia. Rev Bras Ginecol Obstet **2018**; 40:540–546.

220. Read TRH, Murray GL, Danielewski JA, et al. Symptoms, Sites, and Significance of Mycoplasma genitalium in Men Who Have Sex with Men. Emerg Infect Dis **2019**; 25:719–727.

221. Fernández-Huerta M, Espasa M. Mycoplasma genitalium co-infection with Chlamydia trachomatis and Neisseria gonorrhoeae among asymptomatic patients: the silent wick for macrolide resistance spread. Sex Transm Infect **2019**; 95:391.

222. Nodjikouambaye ZA, Compain F, Sadjoli D, et al. Accuracy of Curable Sexually Transmitted Infections and Genital Mycoplasmas Screening by Multiplex Real-Time PCR Using a Self-Collected Veil among Adult Women in Sub-Saharan Africa. Infect Dis Obstet Gynecol **2019**; 2019:8639510.

223. Fernández-Huerta M, Barberá M-J, Esperalba J, et al. Prevalence of Mycoplasma genitalium and macrolide resistance among asymptomatic people visiting a point of care service for rapid STI screening: a cross-sectional study. Sex Transm Infect **2020**; 96:300–305.

224. Bradley I, Varma R, Knight V, et al. Prevalence of rectal Mycoplasma genitalium and macrolide resistance in men who have sex with men attending Sydney Sexual Health Centre. Sex Health **2020**; 17:114–120.

225. Berçot B, Charreau I, Rousseau C, et al. High Prevalence and High Rate of Antibiotic Resistance of Mycoplasma genitalium Infections in Men Who Have Sex With Men: A Substudy of the ANRS IPERGAY Pre-exposure Prophylaxis Trial. Clin Infect Dis **2021**; 73:e2127–e2133.

226. Gragg SD, Gupta KA, Olson KM, et al. Mycoplasma genitalium Infection in Young Women Without Urogenital Symptoms Presenting to a Community-Based Emergency Department in Birmingham, Alabama. Sex Transm Dis **2021**; 48:e27–e29.

227. Suehiro TT, Gimenes F, Souza RP, et al. High molecular prevalence of HPV and other sexually transmitted infections in a population of asymptomatic women who work or study at a Brazilian university. Rev Inst Med Trop Sao Paulo **2021**; 63:e1.

228. Ando N, Mizushima D, Takano M, et al. High prevalence of circulating dual-class resistant Mycoplasma genitalium in asymptomatic MSM in Tokyo, Japan. JAC Antimicrob Resist **2021**; 3:dlab091.

229. Corgie L, Huiban N, Janvier F, et al. Prevalence of leukocyturia in a cohort of French asymptomatic aircrews. Clin Microbiol Infect **2022**; 28:141.e1-141.e4.

230. Lesiak-Markowicz I, Tscherwizek C, Pöppl W, Mooseder G, Walochnik J, Fürnkranz U. Prevalence of selected sexually transmitted infectious agents in a cohort of asymptomatic soldiers in Austria. Parasit Vectors **2022**; 15:424.

231. Dias BDC, Sekgele W, Nhlapo D, et al. Extragenital Sexually Transmitted Infections Among High-Risk Men Who Have Sex With Men in Johannesburg, South Africa. Sex Transm Dis **2024**; 51:245–250.

232. Klein JMA, Runge I, Pannen A-K, et al. Prevalence of bacterial vaginosis, sexually transmitted infections and their association with HPV infections in asymptomatic women attending antenatal care in Ethiopia. Ecancermedicalscience **2024**; 18:1783.

233. Mukavhanyedzi D, Rukasha I. Sexually transmitted pathogens in asymptomatic women at Rethabile clinic, Limpopo, South Africa. S Afr J Infect Dis **2024**; 39:618.

234. Sánchez Navarro JP, Barriga Angulo G, Mata Marín JA, Rodríguez Evaristo M, Padilla Noguera PE, Gaytán Martínez JE. High Prevalence of Asymptomatic STIs in MSM PWH in a Male HIV Clinic in Mexico City. J Int Assoc Provid AIDS Care **2025**; 24:23259582251321039.
